# Scalable-Designed Photonic Metamaterial for Color-Regulating Passive Daytime Radiative Cooling

**DOI:** 10.1007/s40820-025-01975-y

**Published:** 2026-01-12

**Authors:** Xiao-Qing Yu, Fucheng Li, Jiawei Wang, Nianxiang Zhang, Guo-Xing Li, Yan Song, Qing Li, Su Chen

**Affiliations:** State Key Laboratory of Materials-Oriented Chemical Engineering, College of Chemical Engineering, Nanjing Tech University, Nanjing, 210009 People’s Republic of China

**Keywords:** Photonic crystal, Monodispersed latexes, Passive daytime radiative cooling, Assembly regulation, Sub-ambient cooling

## Abstract

Methods allowing passive daytime radiative cooling (PDRC) to be carried out in an energy-efficient and scalable way are potentially important for various disciplines. Here, we report a sustainable strategy for scalable-designed and color-regulating PDRC coating based on high-crystallinity photonic metamaterial (crystallinity: 71.5%; enhanced assembly efficiency: 72%), that is derived from the as-prepared 55 wt% solid content poly(methyl methacrylate-butyl acrylate-methacrylic acid) P(MMA-BA-MAA) monodispersed latexes (approaching theoretical limit: 59 wt%). Robust meter-scale PDRC coatings are constructed by various industrial modes onto diverse surfaces, addressing bottlenecks like dull appearance, high cost, low efficiency, and hard construction. Notably, the solar reflectance, long-wave infrared emittance, and calculated theoretical cooling power of the designed PDRC coating, respectively, reach ~ 0.94, ~ 0.97, and ~ 95.5 W m^−2^ under solar radiation, which can achieve an average 5.3 °C sub-ambient daytime temperature drop in the summer in Nanjing. The cooling performance, scale preparation, and cost-effectiveness of the PDRC coating have extended into leading position compared with those of state-of-the-art designs. This work provides promising route to reduce carbon emissions and energy consumption for global sustainability.

**Supplementary Information:**

The online version contains supplementary material available at 10.1007/s40820-025-01975-y.

## Highlights

- The 55 wt% solid content monodispersed latexes were synthesized under the synergistic action of ionic and nonionic surfactants.
- The 55 wt% solid content monodispersed latexes open a homogeneous assembly avenue, establishing high-crystallinity photonic metamaterial (crystallinity:71.5%).
- We developed scalable-designed and color-regulating passive daytime radiative cooling coating based on the high-crystallinity photonic metamaterial, showing high solar reflectance (~ 0.94), high infrared emittance (~ 0.97), large sub-ambient cooling temperature (average 5.3 °C), and great cooling power (~ 95.5 W m^−2^).

**Supplementary Information:**

The online version contains supplementary material available at 10.1007/s40820-025-01975-y.

## Graphical abstract

**Figure:**
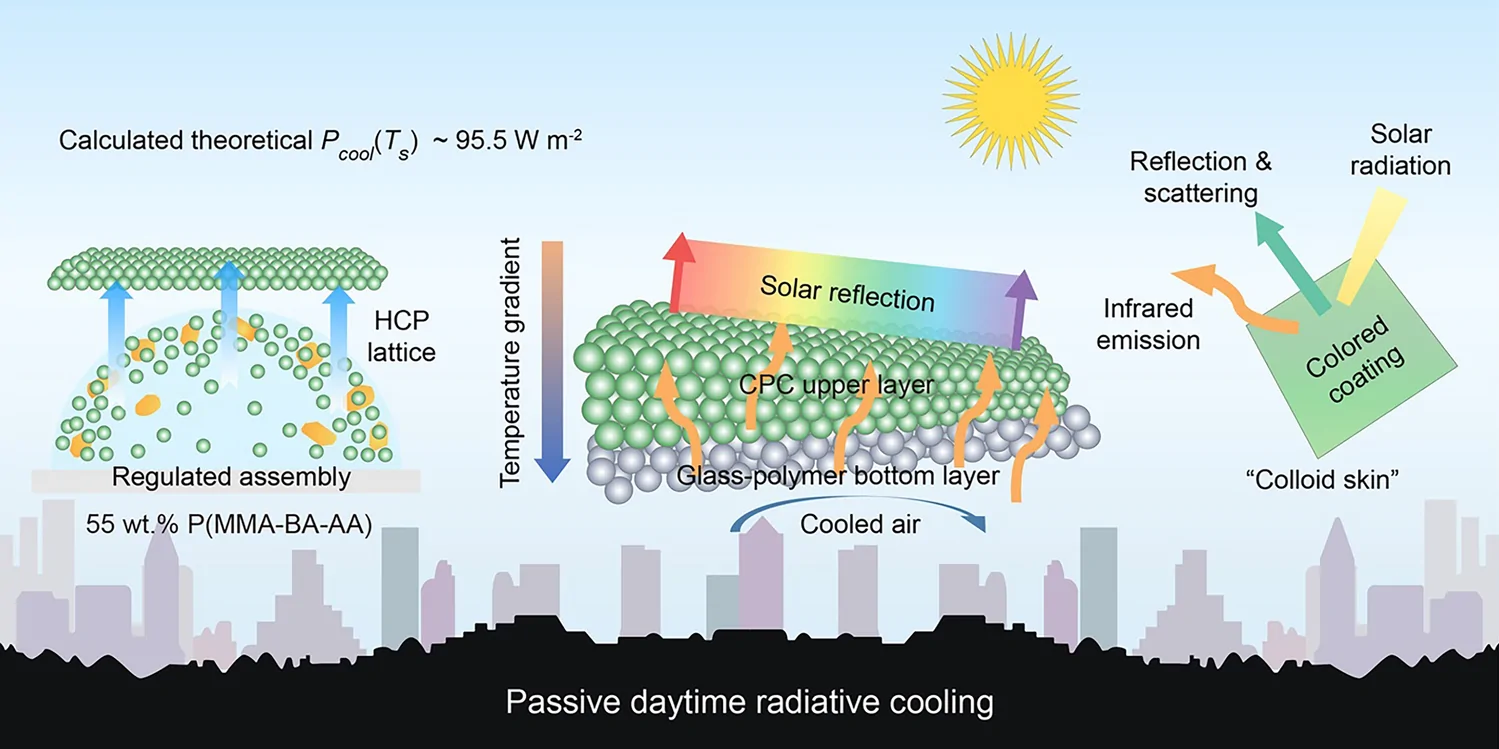

## Introduction

From the perspectives of global total energy consumption, ~ 51% of residential housing energy in the United States and above 50% in China are consumed for maintaining the desired indoor temperature (~ 22 °C) [1, 2]. Especially in hot summer, conventional cooling systems that have been prevalently used require substantial amounts of power input and coolant consumption, which pose global threats of greenhouse gas emissions and urban heat island effect [3]. The escalating energy crisis and climate challenges have intensified the urgency for sustainable cooling. Radiative cooling (RC) emerges as a transformative solution enabling sub-ambient cooling, which cools terrestrial objects by dissipating thermal radiation to the ultracold outer space (~ 3 K) through the atmospheric transparent spectral window (ATSW: 8 to 13 µm) [4, 5]. As a spontaneous, energy-free, and environment-friendly cooling technique, RC addresses critical challenges, including building energy saving, human thermal management, and synergistic cooling applications [6–8]. In this aspect, various promising RC designs have been extensively explored, such as hierarchical porous polymer [9], dielectric particle-embedded coating [10], fiber-structured film [11], and even metamaterials [12]. These designs hold significant influence and support for more sustainable and carbon–neutral development.

In the contemporary era, photonic engineering, which enables synergistic sunlight reflection and thermal emission, has gained considerable attention for efficient daytime cooling [13, 14]. Photonic crystal (PC), a distinctive class of photonic metamaterial, have been developed for passive daytime radiative cooling (PDRC) based on manipulating light-matter interactions at subwavelength scales [15–17], e.g., photonic-structure colored radiative coolers (measured cooling power: ~ 51.6 W m^−2^) [18], flexible hybrid photonic films (theoretical cooling power: ~ 99.8 W m^−2^) [19], and mesoporous photonic coating (theoretical cooling power: ~ 72 W m^−2^) [20]. However, these studies are largely grounded in theoretical perspectives, and the associated materials are high cost, low yield, along with hardly scale-up generation, which limits their manufacturability. Consequently, it remains a challenge to develop PDRC design with photonic structures for desired real-world applications.

**Fig. 1:**
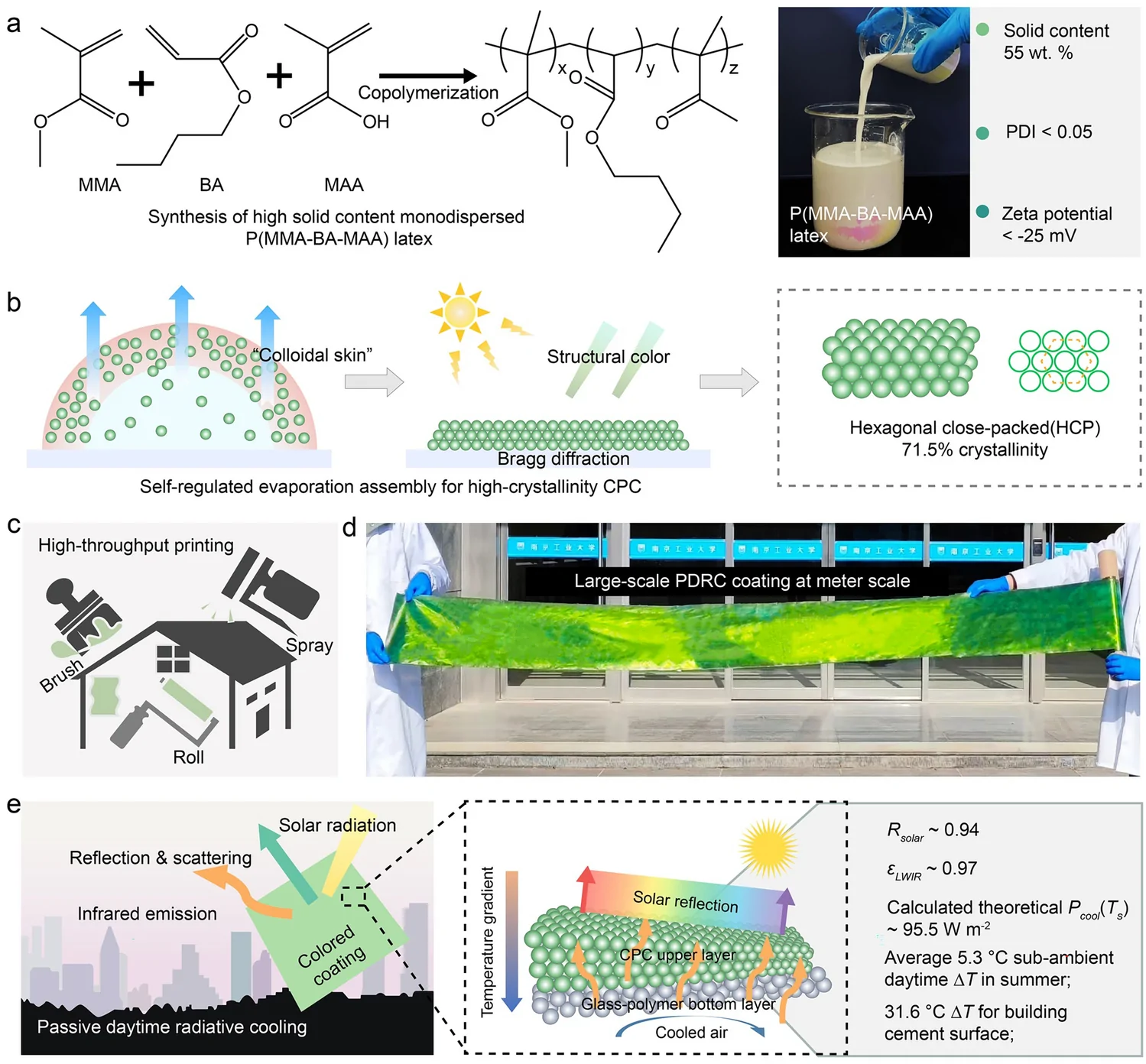
High SC monodispersed latexes for color-regulating PDRC coating. **a** Emulsion polymerization protocol for P(MMA-BA-MAA) monodispersed latex with 55 wt% SC. The milk-like latex with high monodispersity (PDI < 0.05) and stability (Zeta potential < − 25 mV) appears iridescence. **b** Scheme of the self-regulated evaporation assembly of 55 wt% SC monodispersed latexes. “Colloid skin” ensures the achievement of high-crystallinity (crystallinity: 71.5%) photonic metamaterial that exhibits brilliant structural colors. **c** Large-scale color-regulating PDRC coating can be high-throughput painting on substrates by virtue of industrial coating modes, including brush, roll, and spray. **d** Photograph (incident light angle: normal direction; illumination types: sunlight; shooting angle: normal direction) showing the color-regulating PDRC coating at meter scale (40 cm by 240 cm) on cloths produced via a brush coating. **e** Schematic of the hierarchical coating for color-regulating PDRC. The hierarchical structure (photonic metamaterial upper layer and glass-polymer bottom layer) responds to solar and infrared radiation

We present here an easy-to-perform and scalable approach to enable a versatile color-regulating PDRC coating based on the high-crystallinity photonic metamaterial, colloidal photonic crystal (CPC), self-assembled from as-prepared monodispersed colloidal latexes with solid content (SC) up to 55 wt%. The structural color of CPC could extend the palette of available colors of PDRC designs, providing aesthetically desirable colors and patterns in real-world applications [21, 22]. This work shows the following distinct advantages: (1) As a revolutionary material in optoelectronics, monodispersed particles play a driving role in economic development and human civilization [23, 24]. However, the existing problems remain in the synthetic methodology and high-efficiency preparation on a large scale. Generally, the commonly used monodispersed colloidal latexes have rather low SC of only about 5–10 wt%, which has been prevalent for 65 years [25, 26]. In the example illustrated in Fig. 1a, we developed an economical and feasible emulsion polymerization method based on the synergistic action of ionic and nonionic surfactants to achieve the synthesis of poly(methyl methacrylate-butyl acrylate-methacrylic acid) (P(MMA-BA-MAA)) monodispersed latex with desired high SC (55 wt%), and show promise as economical and environmentally friendly PDRC candidate materials. (2) Within the field of nanotechnology, monodispersed particles have been considered as one of the most prominent and promising alternatives for CPC, where monodispersed particles spontaneously organize into ordered microstructures to manipulate photon transfer in their photonic band gap (PBG) [27, 28]. The achievement of near-periodic photonic metamaterial relies on the self-assembly of monodispersed particles, which is highly desired to break the obstacles of tedious preparation procedures and strict technical requirements in the physical pathway [29]. Meanwhile, the interfacial growth model of the 55 wt% SC monodispersed colloidal latex reveals the “colloidal skin”-regulated assembly mode. Here, the “colloidal skin” opens a homogeneous assembly avenue for photonic metamaterial, hence establishing high-crystallinity (experimental crystallinity: 71.5%) hexagonal close-packed (HCP) lattice structures (Fig. 1b). The temperature drop (Δ*T*) of the high-crystallinity photonic metamaterial on building cement reaches 25.4 °C, indicating the advantages of passive cooling capacity.

The product, color-regulating PDRC coating, shows high flexibility for versatile surface adoptions and enhanced assembly efficiency (increased by 72%), allowing it to be competent for various painting modes, such as spraying, rolling, and brushing (Fig. 1c). Especially, the practicability, similar to commercial waterborne coating, enables the PDRC coating to be firmly coated on meter-scale (40 cm by 240 cm) substrates, exhibiting brilliant and robust structural color (Fig. 1d). A 200-μm-thick photonic metamaterial coating shows an averaged infrared emissivity of ~ 0.97 and solar reflectivity of ~ 94% when coupled with a glass-polymer bottom layer (350-μm-thick) (Fig. 1e). Interestingly, these optical performances of the PDRC coating render it with excellent capacity for daytime cooling, exemplified by a calculated theoretical cooling power of ~ 95.5 W m^−2^ and an average 5.3 °C sub-ambient daytime Δ*T* in the summer in Nanjing, which reaches a high level. The effective cooling for existing building cement shows the potential to save ~ 20% of electric energy consumption. The large-scale construction, regulated color profile, desired cooling capacity, robust scrubbing resistance, and feasible industrialization of the PDRC coating ensure the sustainable development of cooling designs.

## Experimental Section

### Materials

Three monomers of methyl methacrylate (MMA), butyl acrylate (BA), and methacrylic acid (MAA) purified by multiple alkaline washing, nonionic surfactant CO897, anionic surfactant sodium dodecyl sulfate (SDS), sodium bicarbonate (NaHCO_3_), potassium persulfate (KPS), and polyvinylpyrrolidone (PVP) were used for the synthesis of P(MMA-BA-MAA) monodispersed latex with high SC of 55 wt%. Extra reagents of tetraethyl orthosilicate (TEOS), polyurethane (PU), methyl silicone oil, ethanol (EtOH), and ammonium hydroxide (NH_3_·H_2_O) were also employed in this work. All regents were purchased from formal suppliers. Ultrapure water with a resistivity greater than 18 MΩ cm was utilized in the whole experiment.

### Synthesis of High SC P(MMA-BA-MAA) Monodispersed Colloidal Latex

The high SC P(MMA-BA-MAA) monodispersed latex was synthesized by developed emulsion polymerization method. Typically, to synthesize a 55 wt% SC latex with particle size of 324 nm and PDI of 0.047, 0.3 g CO897, 0.05 g SDS, 0.03 g PVP, and 0.5 g NaHCO_3_ were completely dissolved in 100 g ultrapure water as a base solution, and then transformed into a nitrogen-protected four-necked flask equipped with fixed stirring speed. Then the system was heated up to 85 °C for 30 min. Subsequently, the pre-emulsion solution (33 g BA, 131 g MMA, 16 g MAA, 1.05 g CO897, 0.4 g SDS, 0.04 g PVP, and 15 g H_2_O) was added dropwise into the four-necked flask, followed by a slow injection of KPS solution (0.5 g KPS dissolved in 15 g deionized water) at 5 mL h^−1^ to initiate polymerization. And the reaction was continued for another 7 h. The resultant high SC P(MMA-BA-MAA) monodispersed colloidal latex was filtered and dialyzed to remove impurities.

### Fabrication of Large-Scale Photonic Metamaterial Coatings and Patterns

The large-scale fabrication of CPC coatings was successfully achieved through industrial-scale coating techniques, including high-throughput spray coating, brush coating, and roller coating, in collaboration with Jiangsu Fengcai Building Materials (Group) Co., Ltd.. The resultant 55 wt% SC P(MMA-BA-MAA) monodispersed latex was blended with 2 wt% PU, and then it was coated on the substrate. Following controlled evaporation and self-assembly, a mechanically robust and large-scale CPC coating with uniform and bright structural color was obtained. Additionally, large-scale CPC pattern customizations can be realized by using masks.

### Construction of Levitating Spherical Colloidal Droplet

The colloidal droplets were constructed by a continuously controllable droplet microfluidic chip. The chip was composed of two round capillaries with port sizes 150 and 200 μm, respectively, and a square capillary (side length, 3 mm). And these tubes were arranged and bound with epoxy resin. The P(MMA-BA-MAA) monodispersed colloidal latex as inner fluid and methyl silicone oil as outer fluid were pumped into the chip. Then, the levitated colloidal droplets were collected into a petri dish containing methylsilicone oil.

### Design of the Hierarchical PDRC Coating

The 43 wt% SiO_2_ particles (covering the particle size range from 200 to 1200 nm) were incorporated into PU to prepare the bottom layer of the PDRC coating. The thickness of the bottom layer was kept around 350 μm using an adjustable coater. After pre-curing at ambient temperature for 30 min, a ~ 200 μm-thick photonic metamaterial coating was deposited as the upper layer. A colored PDRC coating would be achieved through curing again.

### Characterization

The morphology and assembled structure of particles were observed by scanning electron microscope (SEM, HITACHI S-4800). Atomic force microscopy (AFM) images were obtained using Bruker Dimension Icon scanning probe microscope. Fourier transform infrared spectrometer (FT-IR) spectrometer (Nicolet 6700) was utilized to collect FT-IR spectra. Particle size, PDI value, and Zeta potential of latex were measured by dynamic light scattering (DLS) (Malvern, ZEN3690). Stereoscopic optical microscope (SHUNYU SZM45) equipped with a color CCD camera was used to take the optical images. The lattice structure of the assembled CPCs was characterized using small angle X-ray scattering (SAXS) diffractometer (Xeuss 3.0) equipped with Eiger2R 1M detector. IR thermal imager (FLIR E8) and multi-channel temperature recorder (ST 1004) were employed to trace the temperature profiles. Ambient parameters (ambient temperature, wind speed, humidity, and solar intensity) were tracked by a pint-sized automatic weather station (misol WH2310CA). A Xenon light source system (CEL-PE300L-3A) was employed to simulate solar radiation, and its radiation intensity and spectral distribution were calibrated according to ASTM G155 standards. An optical power meter (CEL-NP2000) was used to ensure the radiation intensity control. And reflection spectra in visible range were recorded using an optical microscope equipped with a fiber optic spectrometer (Ocean Optics, USB4000). Reflectivity was measured in the 0.3–2.5 μm range using an ultraviolet–visible-near-infrared (UV–Vis-NIR) spectrophotometer with integrating sphere (Shimadzu UV-3600i Plus). A FT-IR spectrometer (Nicolet iS50) with integrating sphere was used to measure the emissivity in the 5–16 μm range. Pore size distribution analyzer (PSDA-20) was employed to measure the air permeability performance of the cooling clothes.

## Results and Discussion

### Construction and Characterization for the High-Crystallinity Photonic Metamaterial

**Fig. 2:**
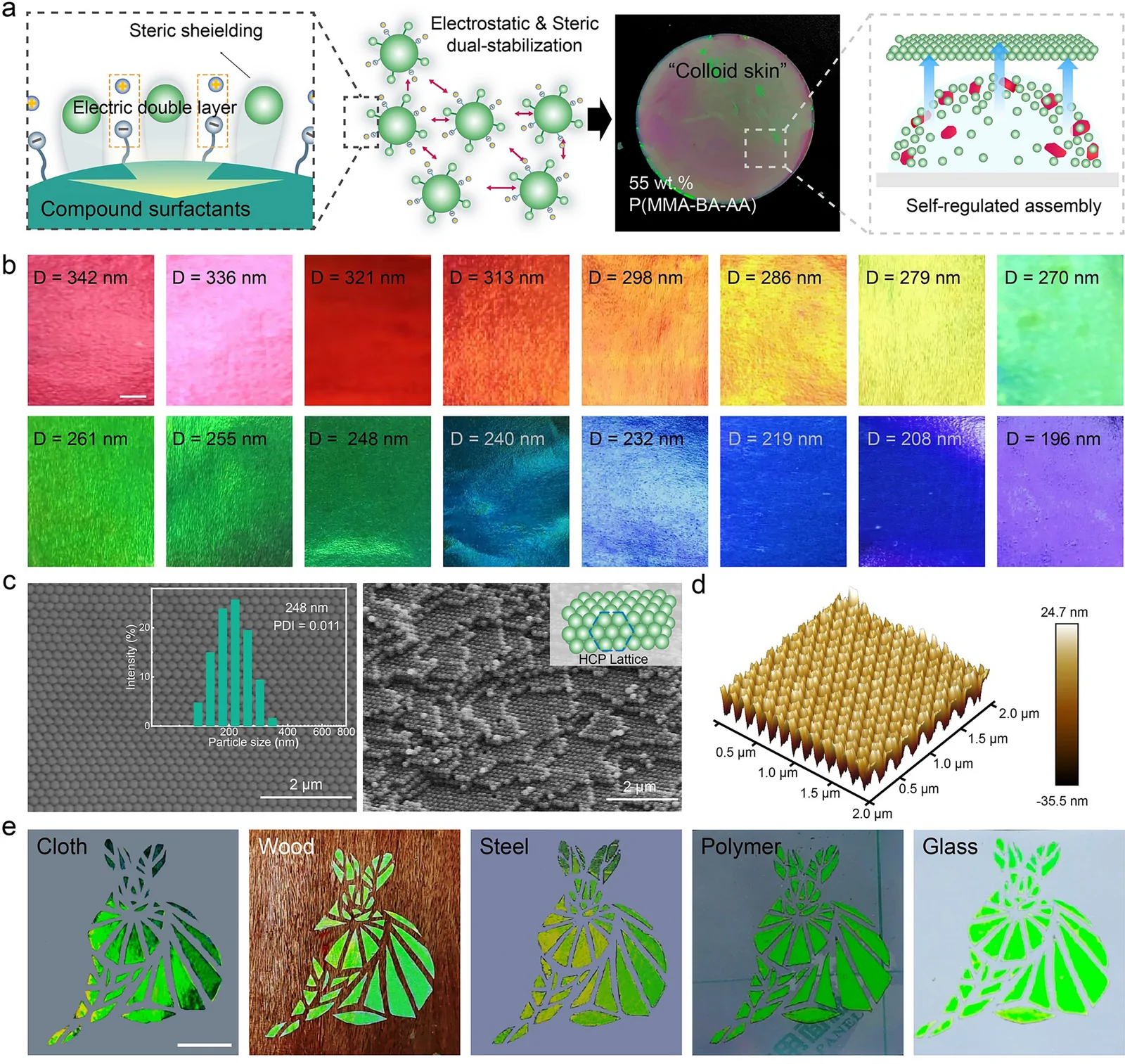
Synthesis and self-assembly of high SC P(MMA-BA-MAA) monodispersed latex. **a** Synergistic action of ionic and nonionic surfactants ensures the formation of monodispersed high SC latexes (55 wt% SC). The as-synthesized high SC latexes tended to form “colloidal skin” for regulating assembly. **b** Photonic metamaterial films with bright structural colors (incident light angle: normal direction; illumination types: fluorescent lamp; shooting angle: normal direction) covering the entire visible spectrum, constructed by high SC P(MMA-BA-MAA) monodispersed latexes with different particle sizes varying from 196 to 342 nm. Scale bar: 1 cm. **c** SEM image showing top view of the assembled photonic metamaterial micrograph. Colloidal particles with spherical morphology and homogeneous size packed into compact hexagonal-shaped arrays. Inset shows the particle size distribution of photonic metamaterial. And SEM image of the cross-section view of assembled photonic metamaterial. High-crystallization HCP lattice throughout the structure. **d** AFM image of the photonic metamaterial. **e** Patterns (incident light angle: normal direction; illumination types: fluorescent lamp; shooting angle: normal direction) formed by directly painting high SC P(MMA-BA-MAA) monodispersed latex onto cloth, wood, steel, polymer, and glass, to appear robust and bright structural color. Scale bar: 5 cm

Giving by Harkins-Smith-Ewart emulsion polymerization model and Derjaguin-Landau-Vervey-Overbeek (DLVO) rule, the situation of monodispersed colloid, especially for the case of its stability and crystallization was concerned (Tables S1 and S2) [30, 31]. Model analysis indicates that the hydration layer thickness of colloidal particles fundamentally governs their maximum theoretical solid content in monodispersed latex systems. The electrostatic layer plays a pivotal role in reducing hydration layer thickness, thereby enabling higher maximum theoretical solid content. Obviously, the method is based on the synergistic action of ionic and nonionic surfactants, ensuring a 15.5 nm-thick hydration layer for stabilizing the 55 wt% SC P(MMA-BA-MAA) monodispersed latexes (Figs. S1 and S2). On a more fundamental level, the maximum theoretical SC was calculated as 59 wt% for a typical P(MMA-BA-MAA) monodispersed latex with 250 nm hydrodynamic diameter (Fig. S3). Apparently, we achieved 55 wt% SC approaches this theoretical limit, which is a fivefold improvement over the current benchmarks. High monodispersity (polydispersion index (PDI) < 0.05) and stability (Zeta potential ~ − 25 mV) of our milk-like 55 wt% SC P(MMA-BA-MAA) latex successfully satisfies the prerequisite to construct cost-effectiveness and high-quality photonic metamaterial (Figs. S4 and S5) [32, 33]. Special fascinating is that iridescence “colloidal skin”, just like the common “milk skin” of hot milky liquid, can be spontaneously formed on the 55 wt% SC monodispersed latex during the evaporation-induced self-assembly, leading to self-regulated assembly for high-crystallinity photonic metamaterial (Figs. 2a and S6, S7, Video S1) [34–36]. Besides, a series of high SC P(MMA-BA-MAA) monodispersed latexes with size gradient were obtained by effectively regulating the reaction parameters (monomer and/or surfactant dosage) (Tables S3 and S4). This experiment proves that bright structural color films could be constructed, covering the whole visible spectra (Figs. 2b and S8).

The present approach is quite general, we were able to observe these photonic metamaterials with high-crystallinity lattice structure through SEM and particle distribution images (Fig. 2c). It is ideal to notice that homogeneous and compact hexagonal-shaped particle arrangements are dominant in the sample constructed by the 55 wt% SC P(MMA-BA-MAA) monodispersed latex. The (100) and the (110) crystal facets of a typical HCP stacking model can be observed in the cross-sectional SEM image [37, 38]. Surface morphology characterization at nanoscale level further confirms the high-crystallinity lattice structure (Figs. 2d and S9). Additionally, the high-crystallinity photonic metamaterials could be constructed onto diverse substrates like cloth, wood, steel, polymer, and glass, forming bright structural color patterns (Fig. 2e).

### Assembly Principle for the High-Crystallinity Photonic Metamaterial

**Fig. 3:**
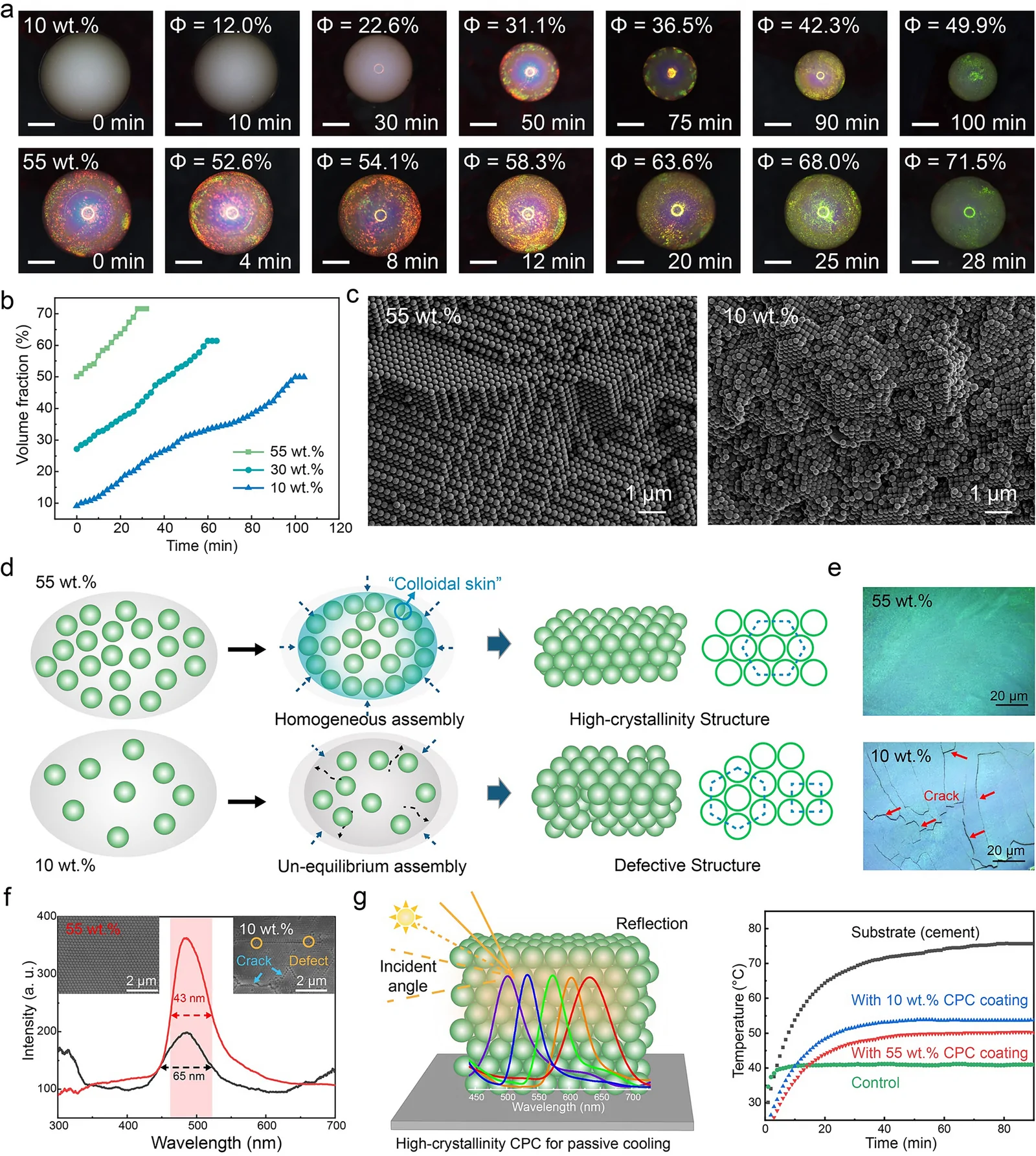
Formation of high-crystallinity photonic metamaterial and their capacity for PDRC. **a** Temporal evolution of individual levitating droplets with different initial particle concentrations 55 wt% (top row) and 10 wt% (bottom row) upon solvent evaporation. The 55 wt% monodispersed latex outperforms that of 10 wt% monodispersed latex in assembly efficiency and crystallinity, resulting in high-quality photonic metamaterial. Scale bar: 200 µm. **b** Temporal changes of *ɸ* for levitating droplets with initial SC of 55 wt%, 30 wt%, and 10 wt% during the evaporation-induced self-assembly. **c** SEM images of photonic metamaterial self-assembled from colloidal droplets with 55 wt% and 10 wt% initial particle concentrations, respectively. **d** Schematics of self-assembly for 55 wt% and 10 wt% monodispersed latexes. High SC monodispersed latex (55 wt%) tends to arrange into HCP lattices. Defective structure is preferred by low SC monodispersed latex (10 wt%). **e** Microscopic optical photographs of photonic metamaterial coating constructed by our high SC (55 wt%) and traditional low SC (10 wt%) monodispersed P(MMA-BA-MAA) latex. **f** Reflection spectra and corresponding SEM images of photonic metamaterial coating originated from our 55 wt% (red curve) and traditional 10 wt% SC latex (black curve). **g** Schematic of high-crystallinity photonic metamaterial for passive cooling. And temperature tracking for the building cement covered by CPC coating assembled from 10 and 55 wt% SC monodispersed latexes under 1000 W m^−2^ simulated solar radiation. Green dotted line indicates the temperature of the control sample (41 °C)

Figure 3a shows the example of single levitating colloidal droplet as a real-time model for monitoring the assembly of photonic metamaterials (Fig. S10) [39, 40]. The droplet with 55 wt% initial SC demonstrates an assembly time as short as 28 min in a constant environment (temperature: 50 °C, humidity: 60%), which is only about 28% of the assembly time for a droplet with 10 wt% initial SC (100 min). Moreover, the 55 wt% SC latex appears structural color in 0 min, while it takes 50 min for the 10 wt% SC latex to show structural color and its volume fraction (*ɸ*) reaches 31.1%. It is calculated that the final *ɸ* of the droplet with 55 wt% initial SC settles at 71.5%, representing a 1.43-fold improvement compared to the droplet with 10 wt% initial SC (49.9%) (Fig. S11). As shown in Fig. 3b, the 55 wt% SC colloidal droplet exhibits a near-linear increase in *ɸ* throughout the assembly process. While for low SC colloidal droplets (30 wt% and 10 wt%), the increase in *ɸ* indicates two different stages. In the early stage, it is slow linear growth, and a sharp rise shows in the late stage. After the evaporation-induced self-assembly, the stacking mode of the 10 wt% SC latex is dominated by defects as indicated in SEM measurements. Whereas the photonic metamaterial with 55 wt% initial SC tends to form the high-crystallinity HCP lattices, which achieves 71.5% calculated crystallinity, greatly higher than the 49.9% value in the 10 wt% SC system (Fig. 3c). These findings demonstrate the superiority of the 55 wt% SC monodispersed latex, particularly in terms of colloidal assembly kinetics and the resulting photonic metamaterial crystallinity.

For the colloidal droplet with high SC of 55 wt%, the particles tend to arrange into a crystalline state, forming colloidal crystals. Furthermore, the interfacial growth model of the droplet reveals an evaporation-driven convection that is much faster than particle diffusion during the self-assembly. Consequently, the 55 wt% SC colloidal particles coalesce at the descending interface and crystallize to form an “iridescent skin”, spreading uniformly across the surface of the droplet [34, 41]. Here, the evaporation flux at the air–liquid interface would be alternated by the spontaneously forming “colloidal skin”, opening a homogeneous assembly avenue for photonic metamaterials. The “colloidal skin”-regulated homogeneous assembly mode of the 55 wt% SC colloidal latex allows crystals to grow radially, hence promoting the uniform and compact distribution of particles, and establishing a high-crystallinity (crystallinity: 71.5%) HCP arrangement throughout the whole assembled structure [37, 42]. While 10 wt% SC colloidal droplet is compelled to arrange into defective structures due to the outward capillary disturbance and prolonged particle diffusion distance [43, 44] (Fig. 3d). The crystallinity of the 55 wt% SC monodispersed colloidal latexes are then enhanced by 43%. Such methods would benefit numerous development efforts by providing preparation strategies for the high-crystallinity photonic metamaterial.

### High-Crystallinity Photonic Metamaterials for Passive Cooling

Taking the film-forming capacity into consideration, dissimilarly, the CPC film derived from the 10 wt% SC latex suffers from dimness and defects. Whereas, the photonic metamaterial assembled from 55 wt% SC latex flashes vivid structural color and appears crack-free morphology (Fig. 3e). Furthermore, the highly regular HCP lattice of the CPC film (from 55 wt% SC latex) was verified in the SEM images, and achieved the construction of high-crystallinity photonic metamaterials. Moreover, SAXS analysis was performed to further demonstrate the lattice structures. 1D SAXS intensity profiles revealed Miller indexes of (1 1 1), (2 0 0), (2 2 0), (3 1 1), (2 2 2), and (4 0 0) crystal facets for the CPCs assembled with 55 wt% initial particle concentrations, confirming the establishment of HCP lattice structures. Meanwhile, the 2D SAXS pattern distinctly presented multiple scattering peaks, indicating the high crystallization and the long-range structural ordering of the resultant photonic metamaterials. In contrast, the sample prepared with 10 wt% SC colloidal particles contributes a single peak and disorder state (Fig. S12) [45, 46]. The corresponding reflection spectrum shows a higher intensity and narrower full width at half maximum (FWHM) of the photonic metamaterials derived form 55 wt% SC latex than that of 10 wt% SC, which is also one of the favorable supports for the high-crystallinity lattice structure (Fig. 3f). Furthermore, angle-dependent diffraction peak modulation of the high-crystallinity CPC demonstrated the long-range ordered lattices (Fig. S13) [47]. Obviously, the high-crystallinity photonic metamaterials originated from the 55 wt% SC P(MMA-BA-MAA) monodispersed latexes effectively combine solar radiation shielding with non-fading structural color appearance, and are particularly suitable for developing colored PDRC designs (Figs. S14 and S15). Through comprehensive assessment of mechanical stabilities, optical properties, and cooling performances, we determined 200 μm to be the optimal CPC coating thickness, which achieves the best balance between these critical parameters. The cooling performance of the high-crystallinity photonic metamaterials on different substrates was explored under 1000 W m^−2^ simulated solar radiation (Fig. S16). Typically, the photonic metamaterial coating (from 55 wt% SC latex) reduced the temperature of building cement from 75.6 to 50.2 °C, while the corresponding photonic coating (from 10 wt% SC latex) kept the temperature at 53.7 °C. The temperature difference of 3.5 °C between the two coatings reveals the excellent passive cooling capacity of the high-crystallinity photonic metamaterial (Fig. 3g).

We further investigated the versatility of our high-crystallinity photonic metamaterial. Interestingly, the unique optical properties of the high-crystallinity photonic metamaterial are complemented with paint-like applicability, which is crucial for practical applications in PDRC designs. The hydrogen bond-mediated enhancement mechanism enabled by 2 wt% PU incorporation ensures excellent mechanical stability of the CPC coatings (Fig. S17) [48]. Remarkably, our 55 wt% SC P(MMA-BA-MAA) monodispersed latex can be readily applied via spraying, rolling, or brushing onto various substrates, forming large-area (> 1 m^2^) CPC coating with extraordinary hiding power and uniform optical property (Figs. S18 and S19, Video S2). Such scalable processing represents a significant advancement over conventional 10 wt% SC monodispersed colloidal latex systems, which typically cannot achieve comparable coating quality or scale. Especially, the photonic metamaterial coating was constructed on building cement. The coating performs an area scale of 0.72 m^2^ and a FWHM of 43 nm, which are dramatically superior to other developed large-scale photonic coatings (Table S5). Taking the possibility of cost-effective fabrication into account, we achieved 1-L-volume monodispersed latex with 55 wt% SC at a time in the laboratory through amplification reaction equipment (Fig. S20). Therefore, we firmly believe further industrial magnification would make the photonic metamaterials comparable in cost to commercial coatings.

### Scalable Design of the Color-Regulating PDRC Coating

**Fig. 4:**
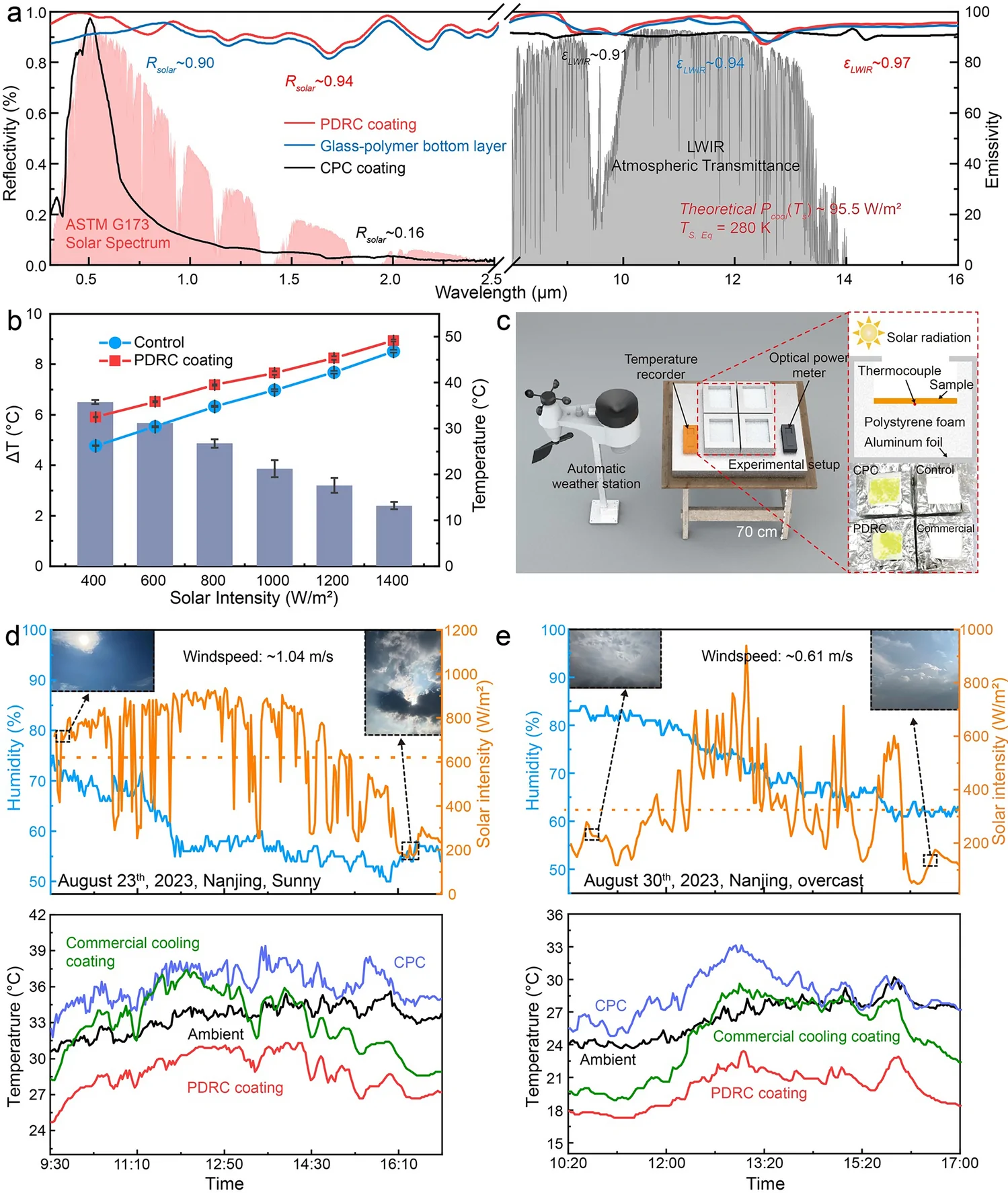
PDRC performance of the designed hierarchical coating. **a** Reflectivity and emissivity spectra of CPC coating (black curve), glass-polymer bottom layer (blue curve), and hierarchical PDRC coating (red curve) from 300 nm to 16 µm. Normalized ASTM G173 solar spectrum (light red shaded area) and empirical LWIR atmospheric transmittance spectrum (gray shaded area) are plotted for reference. **b** Evaluation of cooling performance of the PDRC coating under simulated solar radiation with intensity from 400 to 1400 W m^−2^.** c** Schematic of the setup for the outdoor measurements of PDRC performance in Nanjing, China (32° 6′ N, 118° 40′ E). Temporal temperature data of the ambient, commercial cooling coating, CPC coating, and the hierarchical PDRC coating under direct sunlight** d** in sunny (August 23th, 2023) and** e** in overcast (August 30th, 2023). Detailed *I*_solar_ and humidity data were shown in the upper part, and orange dotted line indicates the average *I*_solar_

Another indication is that most of the PDRC designs are highly dependent on following the spectrum selection requirements. For instance, the object may highly reflect solar radiation (the light red spectrum in Fig. 4a) and greatly emit thermal radiation through ATSW (the gray spectrum in Fig. 4a) [49, 50]. To this end, we designed a hierarchical PDRC coating that offers a broad spectral response across two orders of wavelengths (0.3 to 16 μm) and ensures that the coating synchronously rejects solar radiation and emits thermal radiation in the mid-infrared (MIR) band. The PDRC coating with bright structural color consists of a high-crystallinity photonic metamaterial coating, and a glass-polymer layer is coupled on its back (Figs. S21 and S22). The photonic metamaterials layer is designed as 200-μm thick, which is desirable in its structural color appearance derived from Bragg diffraction of PBG. The bottom layer is a glass-polymer layer, containing SiO_2_ nanoparticles of 200 to 1200 nm (43 wt%) in size and a thickness of ~ 350 μm. The glass-polymer bottom layer could achieve extended emissivity in the ATSW by reaching the high-order Frohlich resonances, and produce high-efficiency scattering covering the visible-near-infrared (Vis–NIR) wavelength based on the joint effect of multiple Mie scatterings (Fig. S23) [12, 51]. However, the broad-spectrum diffusion from the SiO_2_/PU composite bottom layer interferes with the Bragg diffraction of the CPCs, resulting in less vivid coloration compared to pure CPC coatings (Fig. S24) [52, 53]. We then characterized the spectral property of the colored PDRC coating in both the solar band (0.3 to 2.5 μm) and infrared band (5 to 16 μm) by a UV–Vis–NIR and FT-IR spectrophotometer, respectively. The measured spectral reflectivity and emissivity of the samples indicate the thickness-dependent optical properties. The 550-μm-thick PDRC coating reflects ~ 94% solar radiation while keeping nearly statured emissivity of ~ 0.97 in the ATSW, which is distinctly outperforming the 300-μm-thick one (reflectivity: ~ 0.64, emissivity: ~ 92.7%). Comprehensively, the optimal theoretical thickness of the hierarchical PDRC coating is determined to be 550 µm. These experiments not only confirm that the hierarchical PDRC coatings are superior to pure CPC (reflectivity: ~ 0.16, emissivity: ~ 91%) and the glass-polymer bottom layer (reflectivity: ~ 0.90, emissivity: ~ 94%) in the optical aspect, but also render visual artistry for daytime cooling application (Fig. 4a).

The average solar reflectance ${(R}_{\mathrm{solar}})$ and the average long wavelength infrared (LWIR) emissivity $({\varepsilon }_{\mathrm{LWIR}})$ are important parameters to evaluate the cooling performance of PDRC designs. Here, according to the measured spectral reflectivity and emissivity in Fig. 4a, the ${R}_{\mathrm{solar}}$ and ${\upvarepsilon }_{\mathrm{LWIR}}$ of the hierarchical PDRC coating could be determined as ~ 0.94 and ~ 0.97, respectively [54, 55]. Furthermore, the theoretical cooling power (${P}_{\mathrm{cool}}\left({T}_{s}\right)$) of the PDRC coating can be calculated by the following formula [56–58]:

$${P}_{\mathrm{cool}}\left({T}_{s}\right)={P}_{\mathrm{rad}}\left({T}_{S}\right)-{P}_{\mathrm{sun}}-{P}_{\mathrm{atm}}\left({T}_{\mathrm{atm}}\right)-{P}_{\mathrm{cond}+\mathrm{conv}}$$

The cooling performance of the PDRC coating can be quantitatively analyzed through the power balance equation, where higher thermal radiation power (${P}_{\mathrm{rad}}\left({T}_{s}\right)$) and lower solar absorption power (${P}_{\mathrm{sun}}$) collectively enhance the ${P}_{\mathrm{cool}}\left({T}_{s}\right)$. Under idealized conditions (*T*_atm_ = *T*_*s*_ (298 K), atmospheric emissivity = 0.76, and non-radiative heat coefficient (*q*) = 0 W m^−2^ K^−1^), the theoretical ${P}_{\mathrm{cool}}\left({T}_{s}\right)$ of the colored PDRC coating was calculated to be 95.5 W m^−2^. And the equilibrium temperature (*T*_*s, *Eq_) of the PDRC coating was obtained by setting ${P}_{\mathrm{cool}}\left({T}_{s}\right)=0$, resulting in *T*_*s*_, _Eq_ = 280 K. Thus, the designed colored PDRC coating can achieve a maximum sub-ambient daytime Δ*T* of 18 °C. Taking the ${P}_{\mathrm{cond}+\mathrm{conv}}$ into consideration in practical application, when *q* is only 6.9 W m^−2^ K^−1^, the achievable maximum sub-ambient Δ*T* drops to 8 °C, approximately 40% of the idealized value. Additionally, atmospheric emissivity is an important factor affecting PDRC performance. High humidity or heavy cloud will lead to the reduction or even closure of the atmospheric transparent window, so that the ${P}_{\mathrm{atm}}$ increase significantly due to the increase in atmospheric emissivity, and the ${P}_{\mathrm{cool}}\left({T}_{s}\right)$ correspondingly reduce [58]. Thus, the ${P}_{\mathrm{cool}}\left({T}_{s}\right)$ of the colored hybrid coating decreased dramatically from 95.5 to 9 W m^−2^ as the atmospheric emissivity increased from 0.76 (clear sky) to 0.96 (cloudy) (Fig. S25). Although the atmospheric emissivity is < 100% everywhere on Earth, theoretical analysis confirms that lower atmospheric emissivity consistently improves the PDRC performance. The above results collectively demonstrate that the colored PDRC coating maintains efficient cooling performance across diverse operating conditions, highlighting its robust versatility for practical applications.

The high ${R}_{\mathrm{solar}}$ and ${\varepsilon }_{\mathrm{LWIR}}$ allow the hierarchical PDRC coating to achieve exceptional daytime cooling. To validate the theoretical calculations, the next set of experiments was focused on actual cooling performance. Figure 4b quantitatively evaluates the cooling performance of the PDRC coating under simulated solar radiation (indoor) [18, 59, 60]. Corresponding to the control sample, the desired Δ*T* of the PDRC coating demonstrates both robust cooling performance and superior environmental adaptation across a broad radiation intensity ranging from 400 to 1400 W m^−2^ (Figs. S26 to S30). Furthermore, outdoor experiments were performed to demonstrate sub-ambient daytime cooling of the PDRC coating in Nanjing, China (32^o^6’N, 118^o^40’E) using the self-built setup (Figs. 4c and S31). Promisingly, under an average solar intensity (*I*_solar_) of ~ 622 W m^−2^ in sunny (from 9:30 a.m. to 16:45 p.m.), the PDRC coating achieved an average sub-ambient Δ*T* of 4.6 °C. And an average sub-ambient Δ*T* of 6.6 °C was obtained in overcast (*I*_solar_ ~ 320 W m^−2^, from 10:20 a.m. to 17:00 p.m.) (Fig. 4d, e), where the maximum sub-ambient Δ*T* was up to 9.1 °C. The cooling performance of the PDRC coating is significantly better than that of CPC coating (higher than ambient temperature), which is also higher than 3.7–11.5 times that of commercial cooling coating (0.4 °C average sub-ambient Δ*T* under 622 W m^−2^ *I*_solar_; 1.8 °C average sub-ambient Δ*T* under 320 W m^−2^ *I*_solar_) (Figs. S32–S34). Additionally, the outdoor experiments were further performed under typical summer weather in Nanjing for 6 days (Figs. S35 and S36). Consequently, an outstanding sub-ambient daytime cooling of average ~ 5.3 °C under different weather conditions is achieved, which reaches a high level and reveals the all-weather applicability of the PDRC coating (Table S6).

### Experiment for PDRC Applications

**Fig. 5:**
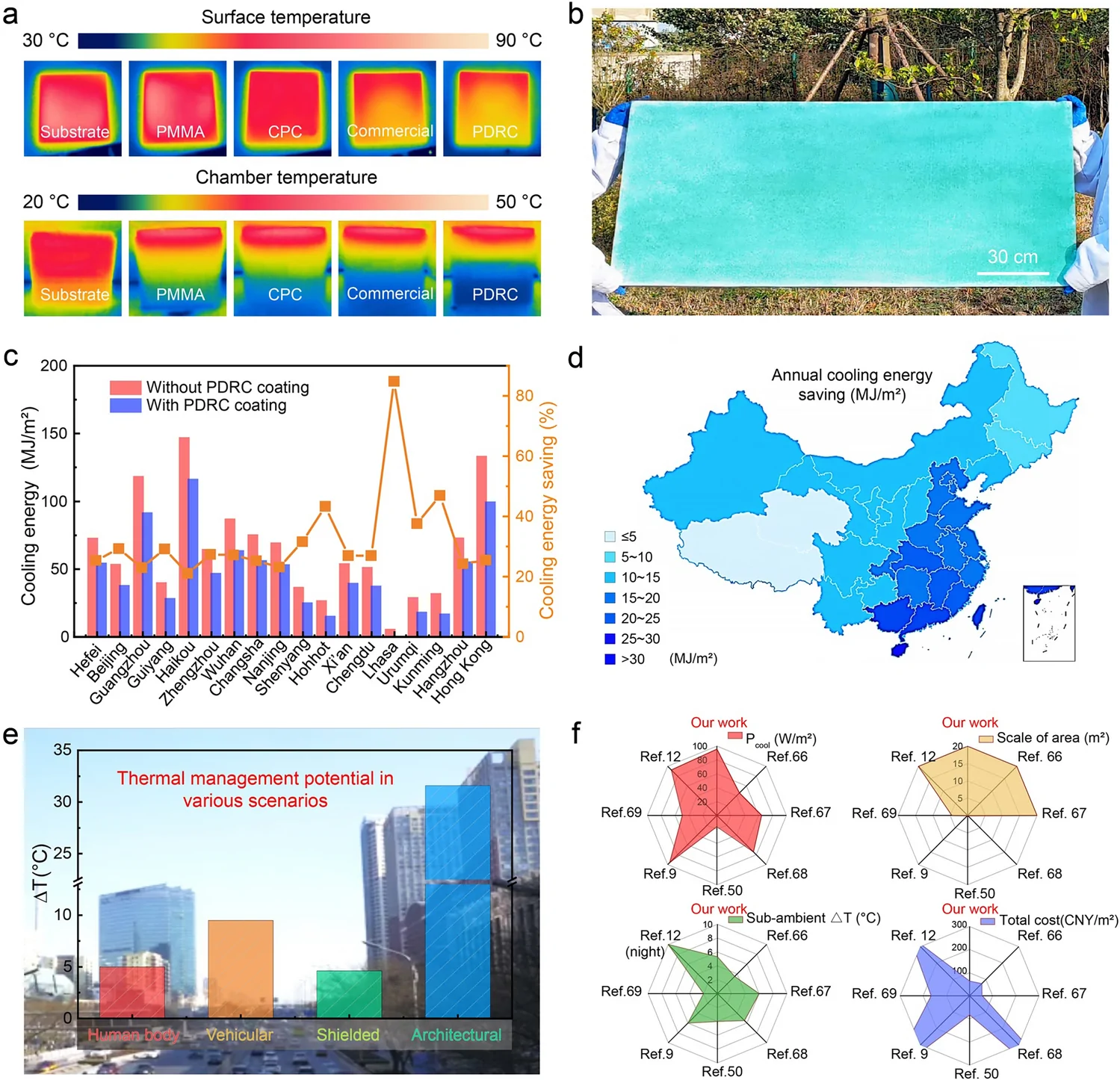
Exploration for cooling application of the designed PDRC coating. **a** Infrared thermal images for the surface and chamber of building cement samples covered by different coatings under 1000 W m^−2^ simulated solar radiation. **b** Photograph (incident light angle: normal direction; illumination types: sunlight; shooting angle: normal direction) showing the colored PDRC coating (60 cm by 120 cm) that was produced on building cement via a roll coating at a speed of ~ 0.2 m^2^ min^−1^. **c** Simulation of cooling energy consumption in building models among 18 representative cities in China. **d** Prediction of annual cooling energy saving for midrise apartment buildings in different regions in China, where the roof and external walls are coated with the PDRC coating. **e** Thermal management potential in various application fields extended for Nanjing based on the cooling capacity of the hierarchical PDRC coating. **f** Comparison of the cooling performance, scale preparation, and cost-effectiveness of the hierarchical PDRC coating with related impactful publications (details in Table S8)

Practically, we have demonstrated the potential application of the hierarchical PDRC coating as building envelopes under 1000 W m^−2^ simulated solar radiation (Fig. S37). The surface (*T*_s_) and chamber (*T*_c_) temperature variations of the samples exposed to the simulated solar radiation were monitored by a thermal camera in the 110-min measurement period, and the corresponding infrared thermal images are shown in Fig. 5a [61, 62]. The *T*_s_ of the bare cement substrate without any shields climbs to 78.2 °C. Whereas, the Δ*T* of 23.7 °C can be attained by CPC coating. This value is obviously superior to the Δ*T* of the transparent PMMA covering without thermoregulatory effect (Δ*T* = 2.8 °C). Promisingly, 31.6 °C Δ*T* was observed on the surface of the cement substrate with the hierarchical PDRC coating, which is even superior to the commercial cooling coating (Δ*T* = 28 °C). While for the *T*_c_, the obvious cooling effect of the independent CPC coating (32.9 °C) can be observed compared with the cement sample (35 °C). Moreover, a lower *T*_c_ (30.4 °C) was obtained by the hierarchical PDRC coating covered cement substrate, further confirming its distinct cooling capability for building thermal management. Obviously, the cooling performance of the samples is highly correlated with their spectral reflectance (Fig. S38). And thus, it can be inferred that effective solar rejection is essential for achieving daytime cooling. Notably, the PDRC coating exhibits strong resistance to weathering and wear. After exposure to rubbing, sunlight, UV radiation, as well as water flushing, the PDRC coating maintains mechanical stability, optical stability, and durability (Figs. S39 and S40, Videos S3 and S4). These preliminary results suggest that the hierarchical PDRC coating possesses stable structural and optical properties, demonstrating its practicability for building thermal management (Fig. 5b). To directly assess the energy-saving potential of the PDRC coating, we performed comprehensive energy consumption simulations depending on a simplified full-scale model of a typical midrise apartment building. The PDRC coating was applied as external envelope material on the walls and roof to quantify the cooling energy savings on a nationwide scale (Fig. S41) [63–65]. Figure 5c depicts the annual energy consumption and savings of the building model in 18 representative Chinese cities spanning diverse climate zones. Considering the long-term climate data in these selected cities, the PDRC coating demonstrates an average annual cooling energy saving of ~ 18.2 MJ m^−2^ compared to the baseline building consumption. Further regional analysis provides valuable insights into climate-zone dependent cooling energy savings (Fig. 5d). The energy-saving potential map highlights the greater potential of the PDRC coating in southern cities compared to their northern counterparts. Meanwhile, it is roughly estimated that the PDRC coating could save ~ 20% (712.5 kWh) of electric energy consumption per year for the typical single-family housing in Nanjing. These combined experimental and theoretical simulation results collectively affirm the exceptional cooling capabilities of our colored PDRC coating, positioning it as a promising solution for energy-efficient and sustainable architectural design.

We envision that the PDRC coating could be further applied to skin-contacting wearable clothes to relieve the heating effects for skin (Fig. S42). However, the air permeability performance of the cooling clothes poses a constraint on its practical application. Besides, the prominent passive cooling capacity of the PDRC coating was adopted for vehicles, where two black car models were used (one served as a control sample and the other was covered by the PDRC coating). And a white car model was employed to exclude the effect of solar heating for black objects (Fig. S43). As expected, the PDRC coating, respectively, achieved average Δ*T* of ~ 9.5 and ~ 4.1 °C compared with the black model and white model during a 4-h outdoor experiment under average 813 W m^−2^ solar radiation. And the PDRC coating could achieve a maximum Δ*T* ~ 15 °C for the black car. In another example, the shielded application of the PDRC coating was explored under the sky. The under-coating temperature was recorded and compared to the corresponding ambient temperature, an average ~ 4.6 °C sub-ambient Δ*T* of the PDRC coating was observed. More importantly, the cooling performance is higher than that of the commercial light-blocking fabric (average ~ 1.4 °C sub-ambient Δ*T*) (Fig. S44). The 3.2 °C temperature difference emphasizes the superiority of our PDRC coating in thermal management. These results demonstrate the potential of the hierarchical PDRC coating for commercial applications in various complex scenarios. Totally, the PDRC capacity of the hierarchical coating appears a significantly value to saving energy consumption associated with cooling (Fig. 5e). Compared with representative large-scale PDRC designs, waterborne coatings [66], cooling coatings [67], structural polymer [68], cooling wood [50], hierarchically porous polymer [9], hierarchical-morphology metafabric [69], and glass-polymer hybrid metamaterial [12], our hierarchical PDRC coating (calculated theoretical ${P}_{\mathrm{cool}}\left({T}_{s}\right)$: ~ 95.5 W m^−2^; scale of area: meter scale; average sub-ambient Δ*T*: 5.3 °C; total cost: 64 CNY m^−2^) has reached the advanced level in cooling performance, scale preparation, and cost-effectiveness (Fig. 5f, Tables S7 and S8). Compared with color PDRC designs that have been published, our hierarchical PDRC coating also demonstrates merits in both scalability and cooling performance (Table S9). Promisingly, our hierarchical coating successfully combines PDRC and coloring without compromising compatibility, which not only constitutes an important advancement in the PDRC designs but also opens up new possibilities for cooling applications.

## Conclusions

In summary, we demonstrated an easy-to-perform and scalable strategy for color-regulating PDRC coating, which was derived from the high-crystallinity photonic metamaterial assembled from the as-developed 55 wt% SC P(MMA-BA-MAA) monodispersed colloidal latex. The 55 wt% SC monodispersed colloidal latex is available, allowing high-efficient assembly for photonic metamaterial coatings (assembly efficiency improved by 72%). Their high-crystallinity structures present HCP stacking mode, along with 71.5% crystallinity, ensuring the appearance of colored dye-free PDRC design. Especially, meter-scale PDRC coating could be constructed by various painting modes, such as spraying, rolling, and brushing onto diverse substrates, which completes the effective practice of industrial waterborne coatings with large-scale construction, regulated color profile, and robust scrubbing resistance. Our results suggest that solar reflectance, long-wave infrared emittances, average sub-ambient daytime Δ*T* in summer, and calculated theoretical cooling power of the designed PDRC coating reach ~ 0.94, ~ 0.97, 5.3 °C, and ~ 95.5 W m^−2^ under solar radiation, respectively. This superior performance could stand up to other advanced PDRC designs. We also verified the PDRC capacity on building envelopes by real-time temperature tracking of samples, in which the 31.6 °C Δ*T* of our hierarchical PDRC coating on existing building cement shows the advantages in thermal management. Therefore, the widespread application of the PDRC coatings in building presents the potential to achieve an annual cooling energy saving of ~ 18.2 MJ m^−2^. This powerful, scalable PDRC coating promises energy-efficient and sustainable applications in the future, significantly reducing carbon emissions and energy consumption.

## Supplementary Information

Below is the link to the electronic supplementary material.

Supplementary file1 (MP4 23492 kb)

Supplementary file2 (MP4 1311 kb)

Supplementary file3 (MP4 2008 kb)

Supplementary file4 (MP4 4510 kb)

Supplementary file5 (DOCX 22871 kb)
