# Supplementary material for: Scalable-Designed Photonic Metamaterial for Color-Regulating Passive Daytime Radiative Cooling
Source: Nanomicro Lett. 2026 Jan 12;18:153. doi: 10.1007/s40820-025-01975-y (PMC12791094; doi:10.1007/s40820-025-01975-y)
Supplement: Supplementary file 5 — Supplementary file5 (DOCX 22871 kb) [file 40820_2025_1975_MOESM5_ESM.docx]

Supporting Information for

**Scalable-Designed Photonic Metamaterial for Color-Regulating Passive Daytime Radiative Cooling**

Xiao-Qing Yu^1#^, Fucheng Li^1#^, Jiawei Wang^1^, Nianxiang Zhang^1^, Guo-Xing Li^1^, Yan Song^1^, Qing Li^1^*, Su Chen^1^*

^1^State Key Laboratory of Materials-Oriented Chemical Engineering, College of Chemical Engineering, Nanjing Tech University, Nanjing 210009, P. R. China

*^#^*Xiao-Qing Yu and Fucheng Li contributed equally to this work.

*Corresponding authors. E-mail: chensu@njtech.edu.cn (Su Chen); liqing1128@njtech.edu.cn (Qing Li)

**Note S1 Chemical Structure Characterization of the High Solid Content (SC) Monodispersed P(MMA-BA-MAA)** **Latex**

To confirm the chemical structure of the synthesized P(MMA-BA-MAA) particles, we analyzed the micro-IR spectra of the particles (Fig. S1). Obviously, the characteristic peaks of O-H (3435 cm^-1^), -CH_2_- (2955 cm^-1^), and C=O (1723 cm^-1^) are presented on the IR spectrum and corresponding micro-IR images of the resultant P(MMA-BA-MAA) latex, respectively. What’s more, there is no C=C absorption in the entire curve. The result proved the successful synthesis of the P(MMA-BA-MAA) latex through the copolymerization of MMA, BA, and MAA monomers.

**Note S2 Realization of 55 wt% SC Monodispersed P(MMA-BA-MAA) Colloidal Latex**

Latex polymerization is regarded as the most cost-effective and scalable strategy for the synthesis of monodispersed colloidal particles [S1]. Typically, ionic surfactant balances the electrostatic force and diffusion force by establishing an electric double layer, thus ensuring the monodispersity of colloidal particles (Fig. S2a). Non-ionic surfactant, by contrast, mainly prevents the aggregation of colloidal particles by steric shielding protection of the formed hydration layer and makes latex monodispersed (Fig. S2b) [S2, S3]. However, for the synthesis of monodispersed latex with high SC (exceeding 30 wt.%), mono-component surfactant is obviously powerless. The mono-component surfactant is difficult to meet the dual requirements of SC and monodispersity in latex polymerization. The maximum SC of the monodispersed P(MMA-BA-MAA) colloidal latex can reach 30.5 wt.% using the mono-component surfactant (SDS). When the reaction monomer dosage continuously increases to 150 g, the P(MMA-BA-MAA) colloidal latex would no longer maintain monodispersity (polydispersion index (PDI) far beyond 0.05) even if the amount of SDS is increased to 3.6 g (Table S1). Herein, we took an anionic and nonionic compound surfactant system to assist the synthesis of high SC monodispersed P(MMA-BA-MAA) latex (Fig. S2c). The fitting curve of monomer conversion rate and reaction time obtained from the experiment is shown in Fig. S2d. We may reasonably conclude that the method is full accordance with the kinetic characteristics of latex polymerization [S1]. We employed SEM and particle size distribution images to show that the P(MMA-BA-MAA) particles maintained regular spherical morphology and excellent monodispersity during the whole synthesis process (Fig. S4). Dual-component surfactants of SDS (anionic) and CO-897 (nonionic) are simultaneously employed to ensure the monodispersity and high SC of resultant P(MMA-BA-MAA) latex (Table S2). The SC of the P(MMA-BA-MAA) colloidal latex can reach 55 wt.% (approaching the theoretical maximum SC of monodispersed colloidal latex, 59 wt.%) while keeping a PDI value of less than 0.05. Therefore, the synergistic action of ionic and nonionic surfactants ensures the monodispersity (PDI < 0.05) and stability (Zeta potential ~-25 mV) of the resultant high SC P(MMA-BA-MAA) latex by enhancing both electrostatic and steric interaction of colloidal particles (Fig. S5).

**Note S3 Calculation of Maximum Theoretical SC of Monodispersed P(MMA-BA-MAA) Latex**

According to previous research, the thickness of the hydration layer of colloidal nanoparticles can be estimated to be 18 nm [S4]. However, the electric double layer on the particle surface should be taken into account in our work. The thickness (1/*κ*) of the electric double layer can be calculated with the following equation [S5]:

$1/\kappa=\sqrt{\frac{\varepsilon k_{B}T}{{8\pi e}^{2}N_{A}I}}$ (S1)

where *ε* denotes the permittivity of water (78.36 F/m), *k_B_* is the Boltzmann constant (1.38×10^-23^ J/K), *T* is the absolute temperature, *e* is the electron charge, and *N_A_* is Avogadro’s number. *I* presents the ionic strength, which can be calculated by the following equation:

$I=\frac{1}{2}\sum_{i=1}^{n} c_{i}z_{i}^{2}$ (S2)

where *c_i_* is the molality of the ion and *z_i_* is the charge of the ion. In the latex polymerization process, 0.5 g NaHCO_3_, 0.5 g KPS, and 0.45 g SDS were used for the synthesis of 250 nm latex. Thus, *I* = 1.5×10^-2^ mol/L according to the theoretical calculation. In practice, when the *I* of a latex located at 10^-2^~10^-1^ mol/L, the value of 1/κ is in 1.2~3.5 nm. Thus, the thickness of the electric double layer 1/κ could be estimated at 2.5 nm, and the thickness of the hydration layer of colloidal nanoparticles could decrease from 18 nm to 15.5 nm. Thus, the maximum SC of colloidal latex could be calculated following the equation:

$Maximum SC=\frac{{(R-15.5)}^{3}\times74}{R^{3}}\times1.18\times100\%$ (S3)

where R is the particle size in latex obtained from dynamic light scattering (DLS) measurement, the density of the P(MMA-BA-MAA) colloidal particles was regarded as 1.18 g/cm^3^. Theoretically, the maximum SC for a typical P(MMA-BA-MAA) latex with a particle size of 250 nm is 59 wt.%, taking into account the fluidity of latex and the 2.5-nm-thick double electric layer. Therefore, the 55 wt.% SC of the resultant monodispersed P(MMA-BA-MAA) latex could be considered to have reached the highest experimental value, and has gone far beyond current research.

**Note S4 Comparison Between 55 wt.%** **Monodispersed Latex and Traditional 10 wt.% Latex**

Experiments for the copolymerization of MMA, BA, and MAA monomers were carried out via traditional latex polymerization and our polymerization strategy to prove the merits of the high SC latex. For the resultant 55 wt.% SC P(MMA-BA-MAA) monodisperse latex, the initial *ɸ* of particles has far exceeded the threshold value for crystallization (*ɸ* = 30%) [S6]. Particles can spontaneously assemble to form colloidal crystal under the combined action of electrostatic repulsions and van der Waals forces, and thus the latex appeared iridescent. “Colloidal skin”, just as the commonly seen “milk skin’’ of hot milk-containing liquids, can be spontaneously formed on the high SC latex surface during the evaporation process. The “colloidal skin” quickly seals the surface of latex, and greatly regulates the evaporative flux across the air-liquid surface (Fig. S6). Hence, highly uniform and bright photonic metamaterial, colloidal photonic crystal (CPC), can be constructed. The CPC flash vivid structural color with crack-free and ordered structure, which is what numerous application fields are looking for. While Brownian motional particles in milk-white low SC latex were hard to resist the outward capillary flow [S7, S8]. Dissimilarly, the CPC film derived from the 10 wt.% SC latex will suffer from dimness and irregularity, showing whitish color, and obvious cracks and defects (Fig. S7).

**Note S5 Adjustment of Particle Size of the High SC Monodispersed P(MMA-BA-MAA) Latex**

The particle size of the high SC monodispersed P(MMA-BA-MAA) latex could be regulated by adjusting reaction parameters (monomer and/or surfactant dosage). As shown in Tables S3 and S4, raising the amounts of monomers will increase the particle size, but will cause a negative effect on the monodispersity. On the contrary, the addition of excessive surfactants would result in a decrease in particle size. Thus, the monodispersed latexes (PDI < 0.05) with particle size from 196 nm to 342 nm, in order to achieve full visible spectrum structure color, can be obtained by effectively optimizing the dosage of surfactants and monomers.

**Note S6 Observation of Self-Assembly Behavior of Colloidal Particles**

The assembly behavior and crystallization mode of monodispersed colloidal particles are closely correlated with the optical and structural properties of the constructed CPCs. Exactly, the assembly behavior of colloidal particles depends on many factors such as substrate, environment, and initial concentration of colloidal particles [S9, S10]. To further gain the merits of the 55 wt.% P(MMA-BA-MAA) CPCs, we focus on investigating the crystallization behavior of colloidal particles under different initial particle concentrations in this work. Herein, a levitating spherical colloidal droplet served as an independent model to real-time probe assembly of particles [S11]. Colloidal droplets were placed into completely incompatible methyl silicone oil via highly controlled droplet microfluidic technology [S12]. Under the action of interfacial tension, the colloidal droplets present regular spheres suspended in the continuous phase of methyl silicone oil (Fig. S10) [S13]. The levitating spherical colloidal droplet provides isotropic environment for evaporation assembly of colloidal particles, which is a reliable tool for in-situ investigation of colloidal crystallization behavior. Meanwhile, the volume-fraction-mediated colloidal crystallization is a facile template for a levitating spherical colloidal droplet, which enables the monitoring of the assembly behavior by simple optical microscope equipment.

Here, the average *ɸ* (*ɸ*_(t)_) of P(MMA-BA-MAA) colloidal particles in the droplet is calculated from the following formula [S11, S14]:

$\Phi_{(t)}=\Phi_{i}{(\frac{R_{i}}{R_{\left( t \right)}})}^{3}$ (S4)

Where, R_i_ and R_(t)_ represent the initial and current radius of the droplet measured by the optical microscope, respectively. The initial volume fraction ($\Phi_{i}$) of colloidal particles was calculated from the known mass fraction and system density (particle density: 1.18 g/cm^3^; water density: 1 g/cm^3^). Totally, colloidal droplet renders three different states in a typical colloidal crystallization assembly process. Colloidal droplet appears white because of the strong scattering and lacking of ordering in low SC latex. As the *ɸ* of particles progresses to about 20%, the droplet displays weak reddish colour, suggesting the onset of short-range correlations. At around *ɸ* = 30%, the droplet shows pronounced iridescence, indicating the generation of long-range ordered colloidal crystals [S6]. Obviously, the assembly behavior is greatly affected by initial *ɸ* of colloidal particles.

The 55 wt.% colloidal droplet begins with bright orange-red structure color. Upon water evaporation, the droplet is gradually contracted and eventually stabilized at *ɸ* = 71.5%. Meanwhile, the color of the droplet turns to orange, yellow, and finally green. Whereas, for the colloidal droplet with an initial concentration of 30 wt.%, its body initially presents a consistent reddish color. As the *ɸ* of the droplet increases to 29.7%, the bright structural color just starts to come out, and eventually forms a green CPC supraball with a *ɸ* of 61.4%. For colloidal droplet with only 10 wt.% initial concentration, it is milky white. When *ɸ* increases to 22.6%, it becomes reddish. Then the droplet flashes structural color and its *ɸ* increases to 31.1%. Finally, the *ɸ* of the droplet remains at 49.9%, presenting a green structural color (Figs. 3a and S11). Shown in Fig. 3b, for the high SC colloidal droplet of 55 wt.%, there was a near-linear increase in *ɸ* during the whole drying process. While for low SC colloidal droplets (30 wt.% and 10 wt.%), the increase in *ɸ* indicated two different stages. In the early stage, it is a slow linear growth, and a sharp rise shows in the late stage. This phenomenon can be explained as the formation of “colloidal skin” for high SC colloidal droplets, in which “skin”-regulated homogeneous assembly mode was opened.

**Note S7 Optical Properties of the High-Crystallinity Photonic Metamaterial**

The high-crystallinity photonic metamaterial derived from the self-assembly of the as-prepared monodispersed colloidal latexes with SC of 55 wt.% could generate well-defined photonic band gaps (PBGs) in the visible spectrum. The interaction between visible light and periodic lattice structures results in bright structural color corresponding to the PBGs. A Bragg-Snell law was proposed to validate the optical properties of the high-crystallinity photonic metamaterial [S15, S16]:

$\lambda_{max}=2\sqrt{\frac{2}{3}}D\sqrt{n_{eff}^{2}-{sin}^{2}\theta_{i}}$ (S5)

Where *λ_max_* is the wavelength of the Bragg diffraction peak of the high-crystallinity CPC, D is the size of monodispersed particles, *θ*_i_ is the angle of the incident light with respect to the normal (Fig. 13a). And the *n_eff_* is the effective refractive index of the high-crystallinity CPCs, which could be calculated according to the following formula:

$n_{eff}=n_{c}\times f_{c}+n_{m}\times(1-f_{c})$ (S6)

Here, *f_c_* is the stacking density of colloidal particles in the high-crystallinity CPCs, *n_c_* and *n_m_* are the refractive indices of colloidal particles (*n_c_* ~1.48) and air matrix (*n_m_* = 1), respectively. As predicted by Equation S5 and confirmed experimentally (Fig. 13b), the diffraction peak of the high-crystallinity CPCs exhibits a characteristic blue shift with increasing incident angle. Importantly, the diffraction peak of the high-crystallinity CPC is also associated with the size of monodispersed particles. Monodispersed colloidal particles with sizes from 196 nm to 342 nm give bright structural color covering the entire visible spectrum at low angles (Figs. 2b and S8).

**Note S8 Cooling Capacity of the** **High-Crystallinity Photonic Metamaterial**

Recently, passive cooling has aroused hot discussion and wide attention. However, the incorporation of color into the cooling designs remains a challenge. CPCs with periodic dielectric structures exhibit PBGs that enable selective solar reflection while producing vivid structural colors, making them ideal candidates for colored PDRC applications [S17, S18]. As demonstrated in Fig. 14a, the CPC coating with ~400 µm thickness shows narrowband selective reflection with lower reflection peak intensity. In contrast, the reflection peak exhibits a more pronounced improvement when the thickness is reduced to 200 µm due to the enhanced coherent light diffraction in the thinner, non-absorptive CPC layer. Moreover, this thickness reduction also addresses the mechanical stability limitations observed in thicker coatings (Fig. S14b). Meanwhile, the CPC coating presents multiple extinction peaks in the atmospheric transparent spectral window (ATSW), which arise from distinct vibrational modes of the molecular structure of P(MMA-BA-MAA) particles. These features provide thermal radiation capacity for the passive cooling (Fig. S14c). The CPC coating combines thermal radiation capacity, selective solar reflection (λ = 0.3-0.8 μm), and vibrant structural color. Consequently, the high-crystallinity CPC highlights the potential for color-tunable PDRC design (Fig. S14d). Although thicker CPC coating exhibited superior cooling performance, comprehensive evaluation of optical properties, mechanical stability, and cooling performance established 200 μm as the optimal CPC coating thickness for subsequent experiments. In this regard, the reflectance spectra of CPC layers with green, orange, and red structural color exhibit stronger dominance within the high-intensity solar radiation range compared to the blue CPC layer, resulting in more desirable PDRC performance (Fig. S15). The cooling capacity of the high-crystallinity CPC for diverse substrates was investigated under 1000 W/m^2^ simulated solar radiation. Interestingly, it could effectively cool these substrates, and ~25.4, 13.5, 9.5, 3.2, 6.1, and 0.8 ℃ temperature drops (Δ*T)* can be attained for cement, steel, wood, cloth, glass, and PMMA substrates, respectively. This could be attributed to the selective reflection of the high-crystallinity CPC coating for solar, thus preventing the solar heating of substrates. And the temperature of the empty setup was recorded as the control group (41 ℃). By contrast, the CPC coating is unable to cool these substrates below the temperature of the control group, which could be attributed to its insufficient thermal reflection and emission performance (Fig. S16).

**Note S9 Development of Robust Photonic Metamaterial Coating**

Based on the above merits, the resultant 55 wt.% SC monodispersed P(MMA-BA-MAA) colloidal latex demonstrates strong potential to be extended to PDRC coatings. Inevitably, conventional CPC coatings suffer from poor mechanical stability and are easy to peel from the substrate due to insufficient interfacial interactions. To overcome this limitation, we introduced PU as an additive to enhance the mechanical stability of CPC coating. The abundant ester bonds (-COO-) and hydroxy groups (-COOH) in PU chains facilitate robust particle-to-particle and particle-to-substrate interactions through hydrogen bonding (Fig. S17a) [S19]. Fig. S17b presents comparative FT-IR analysis of pure CPCs and PU-enhanced CPCs. Notably, the characteristic peak of O-H stretching vibration (1383 cm^-1^) and hydrogen bonding modes (3283 cm^-1^) exhibit significant spectral intensity enhancement following the PU incorporation. Moreover, micro-IR images further visualize the hydrogen-bond interactions through color-coded intensity distributions. However, the massive PU in colloidal latex tended to form cross-linked polymer network and definitely disrupt CPC ordering [S20, S21]. Meanwhile, the amorphous photonic structure potentially influences the color saturation of CPC via Mie scattering (Fig. S17c) [S22]. Whereas, the adhesion grade already reaches 2 when the incorporation of PU exceeds 2 wt.% according to the ISO 2409 standard via a cross-cut test, meeting industrial coating requirements (Fig. S17d) [S23]. Significantly, the effect of the addition of 2 wt.% PU on the optical properties of CPC is negligible. Thus, robust CPC patterns with bright and saturated structural colors can be firmly and facilely printed on various substrates, including paper, wood, steel, cement, polymer, and glass, with the 2 wt.% PU incorporation. Furthermore, we firstly attempt to use the PU/monodispersed P(MMA-BA-MAA) colloidal latex as coatings, and large-scale CPC coatings were successfully realized. The CPC coatings possess extraordinary hiding power and present uniform and stable optical properties.

**Note S10 Design of the Hierarchical PDRC C****oating**

Inspired by previous research, we proposed a hierarchical PDRC coating with two thermoregulatory effects capable of achieving colored PDRC. The hierarchical coating is constructed via a facile two-step painting process, which is promising for scale-up production (Figs. S21 and S22). The bottom is a glass-polymer metamaterial layer, in which spherical SiO_2_ micro-particles (particle size: ranging from 200 to 1200 nm) were embedded into the polymer matrix for enhancing Mie scattering and IR emissivity. The existence of strong phonon-polariton resonances of the encapsulated SiO_2_ particles would lead to an improvement in emissivity [S24-S26]. Meanwhile, for the optimization of SiO_2_ particle concentration in the glass-polymer bottom layer, we designed samples with different SiO_2_ particle concentrations from 20 wt.% to 50 wt.%. Given the experimental results, one could derive that SiO_2_ particle concentrations above 40 wt.% would highly contribute to the IR emissivity. The cooling capacity of the samples was also explored under 1000 W m^−2^ simulated solar radiation. The 43 wt.% doping amounts of SiO_2_ in the PU matrix can effectively reduce the surface temperature of cement to 54.9 ℃. Based on the trade-off of the cooling and economic performances, ~43 wt.% doping amount of SiO_2_ was adopted in the experiment design and radiative cooling application (Fig. S23).

**Note S11** **Definition of** $\boldsymbol{R}_{\boldsymbol{solar}}$**,** $\boldsymbol{\varepsilon}_{\boldsymbol{LWIR}}$**, and Theoretical Cooling Power of the Hierarchical PDRC Coating**

The optical properties of the hierarchical PDRC coating were characterized by UV-Vis-NIR spectrophotometer and FT-IR spectrometer [S27]. From the measured spectrum, the PDRC coating shows both high reflectivity in the region of incident solar wavelength (0.3-2.5 μm) and thermal emissivity in ATSW (8 to 13 µm), which both are over 90% (Fig. 4a). To achieve efficient PDRC, materials must minimally absorb heat from the sun and maximally radiate heat to the outer cold space [S28, S29]. The $R_{solar}$ and $\varepsilon_{LWIR}$ are important parameters to evaluate the cooling performance of PDRC deisgns. According to Planck's law, the $R_{solar}$ and $\varepsilon_{LWIR}$ of the hierarchical PDRC coating can be calculated [S30, S31].

$R_{solar}=\frac{\int_{0.3}^{2.5} R(\lambda)I_{AM1.5}\left( \lambda\right)d\lambda}{\int_{0.3}^{2.5} I_{AM1.5}\left( \lambda\right)d\lambda}$ (S7)

$\varepsilon_{LWIR}=\frac{\int_{8}^{13} I_{BB}(\lambda)\varepsilon(\lambda)d\lambda}{\int_{8}^{13} I_{BB}(\lambda)d\lambda}$ (S8)

where the *I*_AM1.5_ is the ASTM G173 global solar intensity spectrum, *I_BB_* is the spectral radiance of a blackbody defined by Planck’s law in ATSW. Therefore, according to the measured spectral reflectivity and emissivity of the hierarchical PDRC coating (Fig. 4a), the $R_{solar}$ and $\varepsilon_{LWIR}$ of the PDRC coating can be calculated as ~0.94 and ~0.97, respectively.

Considering entire heat transfer process of the hierarchical PDRC coating with a temperature of *T*_s_, the theoretical cooling power ($P_{cool}\left( T_{s} \right)$) can be calculated by the following formula [S32-S34].

$P_{cool}\left( T_{s} \right)=P_{rad}\left( T_{S} \right)-P_{sun}-P_{atm}\left( T_{atm} \right)-P_{cond+conv}$ (S9)

$P_{rad}\left( T_{s} \right)=\varepsilon_{LWIR}\sigma T_{s}^{4}$ (S10)

$P_{sun}=\int_{0.3}^{2.5} I_{AM1.5}\left( \lambda\right)A\left( \lambda\right)d\lambda$ (S11)

$P_{atm}(T_{atm})=\varepsilon_{LWIR}\varepsilon_{atm}\sigma T_{atm}^{4}$ (S12)

$P_{cond+conv}=q(T_{atm}-T_{s})$ (S13)

Where $P_{rad}\left( T_{s} \right)$ represents the thermal radiation power of the PDRC coating, $P_{sun}$ is the solar radiation power absorbed by the PDRC coating, $P_{atm}$ is the atmospheric long wavelength radiation power absorbed by the PDRC coating, and $P_{cond+conv}$ is the non-radiative power lost due to thermal convection and conduction. Meanwhile, the σ is a Stefan-Boltzmann constant, equals to $5.67\times{10}^{-8} W\cdot m^{-2}K^{-4}$, the atmospheric emissivity (0.76) obtained from standard spectral of the LWIR atmospheric transmittance in ATSW. Figure S25 demonstrated the calculated theoretical cooling power ($P_{cool}\left( T_{s} \right)$) and achievable cooling temperatures (Δ*T*, referring to the temperature difference between the ambient (*T_atm_*) and sample ($T_{s})$) of the PDRC coating under different non-radiative heat coefficient (*q*). To clarify, the theoretical$P_{cool}\left( T_{s} \right)$ of the PDRC coating is calculated as 95.5 W/m^2^ at *ΔT* = 0 ($T_{atm}=298 K)$, where the interference of thermal convection and conduction ($P_{cond+conv}$) could be ignored. Besides, the equilibrium temperature (*T*_s, Eq_) of the PDRC coating is obtained by setting *P_cool_*(*T*_s_) = 0, resulting in *T*_s,_ *_Eq_* =280 K. And thus, the hierarchical PDRC coating can achieve a maximum Δ*T* of 18 ℃ when the *q* = 0. Even with a strong non-radiative heat transfer (*q* = 6.9 W m^−2^ K^−1^), the maximum *ΔT* of the hierarchical PDRC coating can still reach ∼8 °C.

**Note S12** **Experimental Characterization of Cooling Performance under Simulated Solar Radiation (Indoor)**

Building of experimental platform

Self-built experimental setup was used to characterize the cooling performance of samples under simulated solar radiation. The self-built experimental setup mainly consists of aluminum foil (0.15 mm thick)-wrapped polystyrene foam (15 cm×15 cm×10 cm) with a cubic groove (10 cm×10 cm×5 cm) on the top. The polystyrene foam with low thermal conductivity was employed to enhance the thermal isolation, which could reduce the thermal effect of the environment on experiments. And the wrapped aluminum foil can efficiently reflect the sunlight. Meanwhile, a polyethylene (PE) film was applied on the top of the setup as shielding to reduce thermal convection and conduction between samples and the environment (Fig. S26) [S35, S36].

The indoor characterization of cooling performance

In the indoor characterization, the self-built experimental setup was positioned on a 5-cm-thick foam insulation mat beneath a Xenon lamp solar simulator, and the samples were completely exposed to simulated solar radiation. During the experiments, the optical power meter was used to calibrate the solar radiation intensity. Meanwhile, a multi-channel temperature recorder equipped with thermocouples was conducted to collect the ambient and sample temperatures. The tip of the thermocouples is covered with 0.25 mm thick bleached pulp (thermal conductivity, 0.10 W m^−1^ K^−1^; thermal resistance, 0.0025 m^2^ K W^−1^) to prevent interference from solar heating (Fig. S26) [S37]. Here, 194 K drikold was used as a cold sink for thermal radiation, and ambient temperature was kept at ~25 ℃ in all indoor experiments [S38, S39].

**Note S13 Evaluation of Cooling Performance for PDRC Coating under Simulated Solar Radiation**

Practically, a ~550 μm thick PDRC coating was directly constructed into the self-built experimental setup for cooling performance evaluation. Meanwhile, an empty experimental setup without any samples was used as the control group. We compared the temperature difference between the PDRC coating and the control group under simulated solar radiation for 80 minutes. Comparative analysis revealed a sustained temperature differential (*ΔT*) of 3.9 °C between the PDRC coating and control group during 80-minute exposure to simulated solar radiation at 1000 W/m², confirming its advantage in passive cooling. Furthermore, three sets of parallel experiments were carried out to further demonstrate the solar radiation intensity-dependent cooling behavior of the PDRC coating. The near-linear decline in *ΔT* from 6.5 to 2.4 °C occurred as under radiation intensity increased from 400 to 1400 W/m^2^ (Figs. 4b and Fig. S27 to S29). This trend aligns with established PDRC performance models where higher solar fluxes gradually diminish net radiative cooling potential. Notably, the *ΔT* of the PDRC coating could be maintained at 2.4 ℃, even when the solar radiation was raised to 1400 W/m^2^. It is worth mentioning that the cold universe (3 K) endows greater thermal emission space for cooling, and thus, the PDRC coating would show more effective cooling performance for outdoor applications. Taking the angle-dependent reflection into account, we have tracked the cooling performance of the PDRC coating under different solar illumination angles (from 0^o^ to 45^o^) via rotating the setup vertically (Fig. S30). It is found that the temperature of the PDRC coating is gradually reduced, which could be attributed to the angle-dependent reflections and changes in solar reflectivity.

**Note S14 Demonstration of Sub-Ambient Daytime Cooling of PDRC Coating (Outdoor)**

Similarly, the experimental setup with PDRC coating was exposed to sky and direct sunlight for the outdoor experiment, which was performed on the roof of a six-story building in Nanjing, China (32^o^6’N, 118^o^40’E) in late August. The ambient parameters, including ambient temperature, wind speed, humidity, and solar intensity were tracked by a pint-sized automatic weather station. And the temperatures of samples were monitored by the multi-channel temperature recorder. Meanwhile, the whole setup was placed on a platform ~70 cm above the ground (Fig. 4c and Figs. S31-S33). The 6.5 ℃ sub-ambient *ΔT* was achieved with the PDRC coating in cloudy conditions under 632 W/m^2^ solar radiation (1.21 m/s wind speed, 63% humidity). Synchronously, a control experiment without PE shielding was designed to evaluate the utility performance of the PDRC coating in application. A stable sub-ambient cooling of ~5.2 ℃ was still achieved, which is prominent in most scenarios. Additionally, to further verify the robustness of the cooling performance of the PDRC coating, the outdoor experiments were carried out under diverse weather conditions in summer. The PDRC coating also ensures average 4.6 ℃ (622 W/m^2^ solar radiation, 1.0 m/s wind speed, and 59.5% humidity), 5.0 ℃ (580 W/m^2^ solar radiation, 0.75 m/s wind speed, and 50.6% humidity), 5.6 ℃ (637 W/m^2^ solar radiation, 0.84 m/s wind speed, and 46.7% humidity), 6.6 ℃ (320 W/m^2^ solar radiation, 0.61 m/s wind speed, and 71.6% humidity), and 4.7 ℃ (714 W/m^2^ solar radiation, 1.2 m/s wind speed, and 48.7% humidity) sub-ambient *ΔT*, respectively (Figs. S35 and S36, Table S6). These results confirm the robust and desired cooling performance of the PDRC coating, showing its all-weather applicability. Notably, during the outdoor experiments, a control setup without samples was designed to determine the reliability of the temperature measurement method. As shown in Fig. S33, the average 0.06 ℃ temperature difference (absolute deviation: 0.37 ℃) between the control sample and the ambient is totally negligible.

**Note S15 Cooling Energy-Saving Simulations of the PDRC Coating as Building Envelopes**

The cooling energy saving simulations for the PDRC coating were conducted using the open-source software OpenStudio (version 3.7.0). The simplified midrise apartment building model was constructed by SketchUp (version Pro 2022) with 36 m^2^ total floor area, 18 m^2^ roof area, and 118 m^2^ total external wall surface area. The windows occupy 7% of the total external wall surface area (Fig. S41). Through comparative simulations against baseline roof and wall materials, we evaluated the energy-saving potential of the colored hybrid PDRC coating in the building prototype. The internal boundary conditions were set at 25 °C (room temperature), while the external boundary conditions were derived from climate data downloaded from the EnergyPlus website in simulations [S40-S42].

Typically, a typical single-family residence in Nanjing consumes approximately 3600 kWh of electric energy annually for normal household appliance operation [S43]. Taking the climate (95 days of hot weather per year) of Nanjing as an example, the use time of air conditioning system is ~7.5 h/day, the electric energy consumption is ~0.5 KWH per hour [S44, S45], and 2 air conditioning systems work for cooling. In this case, we have roughly estimated the electric energy saving of ~712.5 kWh per year of our colored PDRC coating. Therefore, the application of the colored PDRC coating on the exterior walls and roof of the typical single-family building can achieve ~20% annual electric energy saving.

**Note S16 Application Exploration for Passive Cooling Effect of the Hierarchical PDRC Coating**

Additionally, the potential of the hierarchical PDRC coating has been explored from the visual angle of application. The use of fabric substrates endows the coating with flexibility. The application of wearable products to realize passive cooling for human body was researched based on the excellent flexibility and thermal management performance. As the thermal image in Fig. S42 shows, the hierarchical PDRC coating effectively reduced the temperature (more than 5 ℃) of the covered area of the arm, which is better than white cloth (about 3 ℃). However, the PDRC coating significantly reduced the air permeability, decreasing the gas flux from 4971.41 m^3^/m^2^·h·kPa for the untreated white cloth to 25.97 m^3^/m^2^·h·kPa, which substantially limits its practicality for real-world applications. Moreover, the outstanding cooling performance of our PDRC coating could be applied to vehicles (Fig. S43). We tested the passive cooling performance of the hierarchical coating with two car models covered by our PDRC coating and nothing. And a white car model was employed to exclude the effect of solar heating of black objects on the evaluation of passive cooling capacity. During the outdoor experiment, the ambient temperature was obtained from weather station. And the inner temperature of car model was tracked using the multi-channel temperature recorder, while the thermal image was monitored by a thermal camera. In another experiment, we have conducted an outdoor experiment to evaluate the PDRC potential in shielded application. A 2-mm-thick PMMA plate without thermoregulatory effect serves as the substrate and control group. The ambient temperature and solar intensity data distribution were recorded by the pint-sized automatic weather station, and the sample temperatures were collected using the multi-channel temperature recorder. The similar temperature curves between ambient and control sample reveal the reliability and accuracy of the test data. Obviously, the PDRC coating cools the air by an average sub-ambient *ΔT* of 4.6 ℃, which is even superior to the commercial light-blocking fabric (average ~1.4 ℃ sub-ambient *ΔT*) (Fig. S44). This colored PDRC coating via the effective reflection and strong thermal emission successfully takes account of both cooling and coloring, which represents a major step forward in the field of PDRC designs and offers new insight into functional and scalable applications for architectural cooling and energy saving.

**Note S17 Economic Analysis for** **PDRC Design**

A techno-economic analysis (TEA) model was employed to roughly estimate the cost of PDRC coating [S46]. Here, the total cost of the PDRC design could be considered as the sum of raw material cost and manufacturing costs, which could be calculated by the following formula:

$$Total cost=Raw material cost+Manufacturing cost (S14)$$

$Raw material cost=\sum_{i=1}^{i=n} U.P.\left( i \right)\times R_{i}$ (S15)

$$\mathrm{Manufacturin}g \mathrm{cost}=Manufacturing time\times Unit labor cost+Equipment cost (S16)$$

Where the U. P. (i) is the unit price of component i, and R_i_ is the mass ratio of each component. In our case, a simple brush could be used for the large-scale construction of PDRC coating, such the equipment cost is ignored. And the total cost of our PDRC coating is roughly estimated to be ~64 CNY/m^2^. The costs of raw materials were calculated according to commercial price (China's leading online shopping site), and unit labor cost was defined as ~20 CNY/m^2^ and ~20 CNY/h.

**Supplementary Tables and Figures**


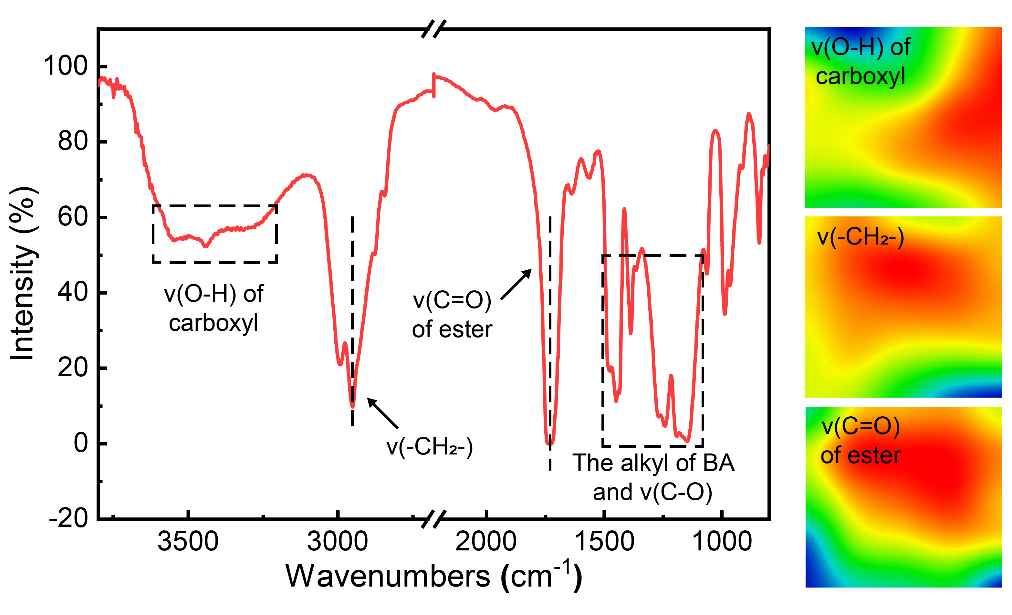


**Fig. S1** IR spectrum of resultant P(MMA-BA-MAA) particles and micro-IR images of corresponding characteristic peaks


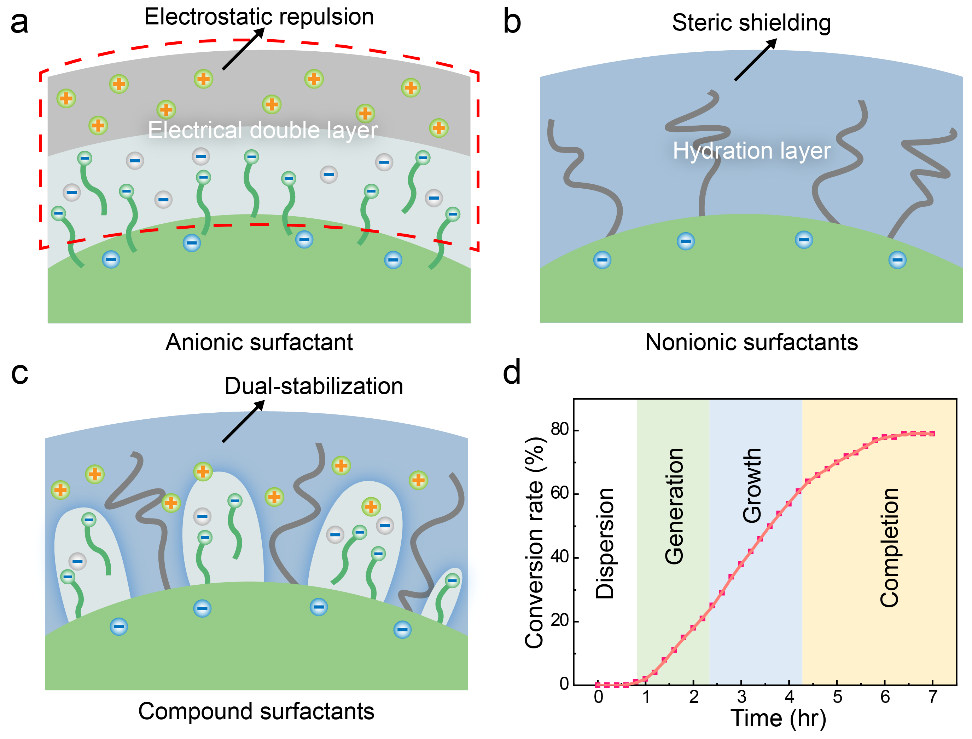


**Fig. S2** Schematics of the stabilization mechanism of latex polymerization assisted by **a** anionic, **b** nonionic and **c** compound surfactants. Ionic surfactant could form electric double layer, while non-ionic surfactant could create hydration layer to maintain particle stability. The synergistic action of ionic and nonionic surfactants ensures the formation of high SC monodispersed latexes. **d** Monomer conversion-time curve of the whole latex polymerization process. The whole reaction process can be divided into four stages of dispersion, generation, growth, and completion, which fully accord with the kinetic characteristics of latex polymerization.


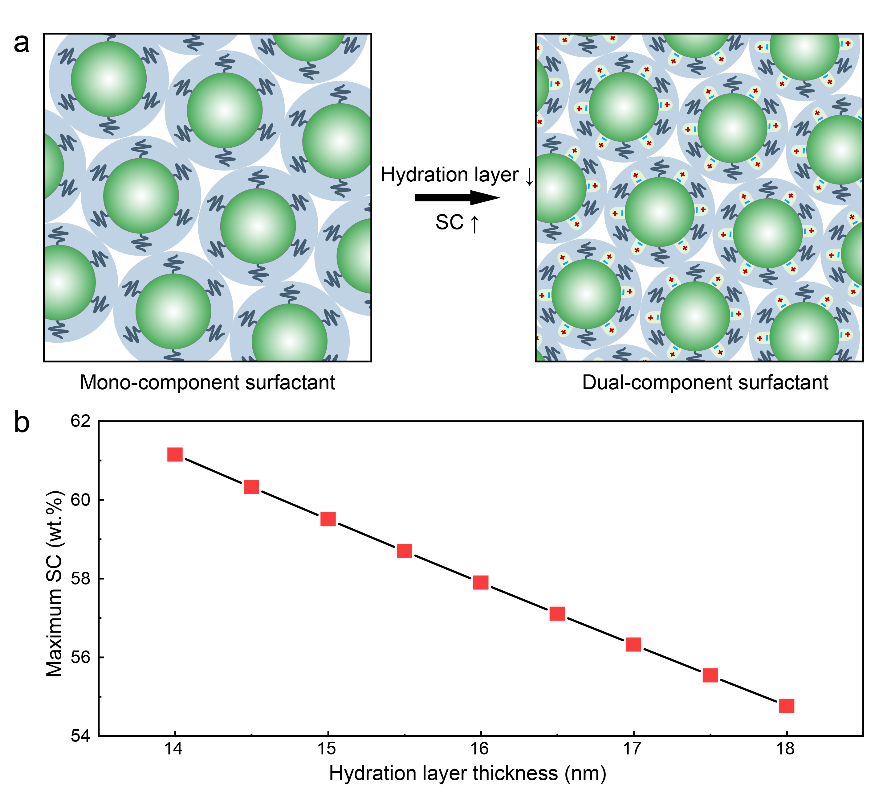


**Fig. S3** **a** Schematic illustration of the hydration layer thickness regulation mechanism mediated by the two-component surfactant system, aiming to enhance the solid content in monodispersed latex. **b** Maximum theoretical solid content of the synthesized monodispersed colloidal latex curves with the hydration layer thickness of colloidal particles. The colloidal particles with hydrodynamic diameter of 250 nm serve as the basis for the calculation.


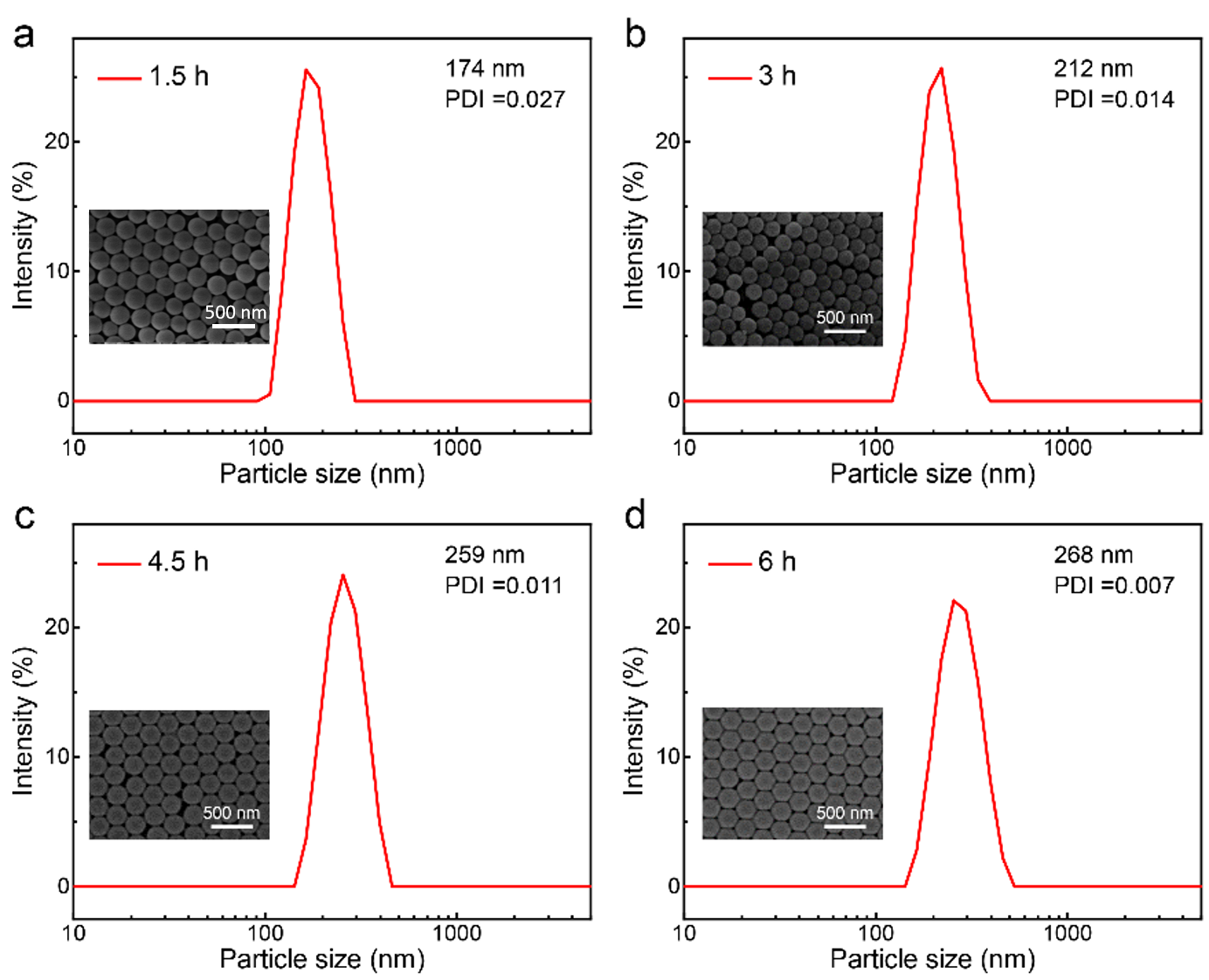


**Fig. S4** Particle size distribution (PSD) and SEM images of the high SC monodispersed P(MMA-BA-MAA) latex (particle size: 268 nm) at different stages of the latex polymerization process: **a** 1.5 h, **b** 3 h, **c** 4.5 h, and **d** 6 h


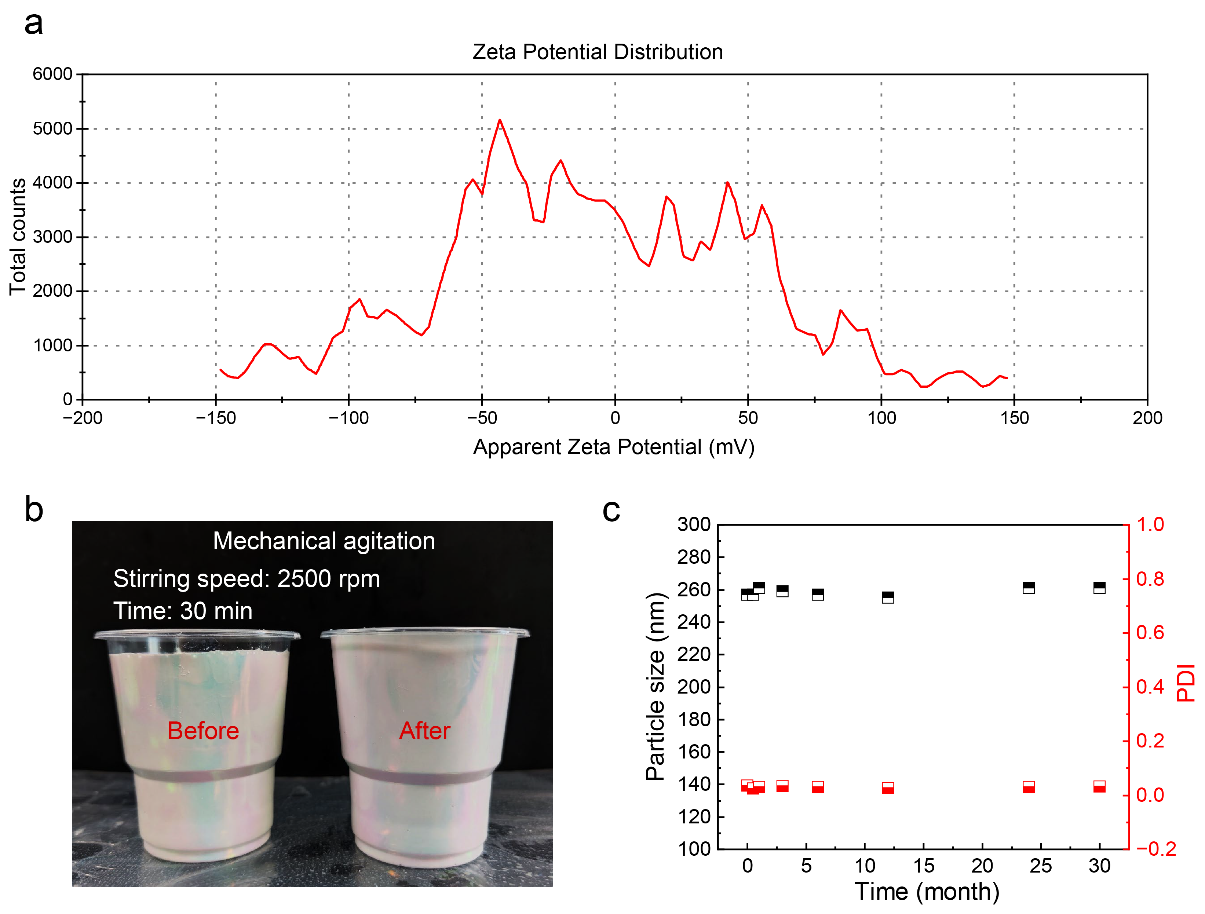


**Fig. S5** Stability characterizations of the high SC monodispersed P(MMA-BA-MAA) latex. **a** Zeta potential measurement of the high SC monodispersed P(MMA-BA-MAA) latex. **b** Photographs of the high SC monodispersed P(MMA-BA-MAA) latex before and after high-speed agitation (2500 rpm) for 30 min. Continuously high-speed agitation did not affect the stability of the latex, and the latex remains uniformly dispersed with iridescent. **c** Particle size and PDI characteristics of the high SC monodispersed P(MMA-BA-MAA) latex at different storage times. There are no significant changes in particle size and PDI of the latex after stored for 2.5 years.


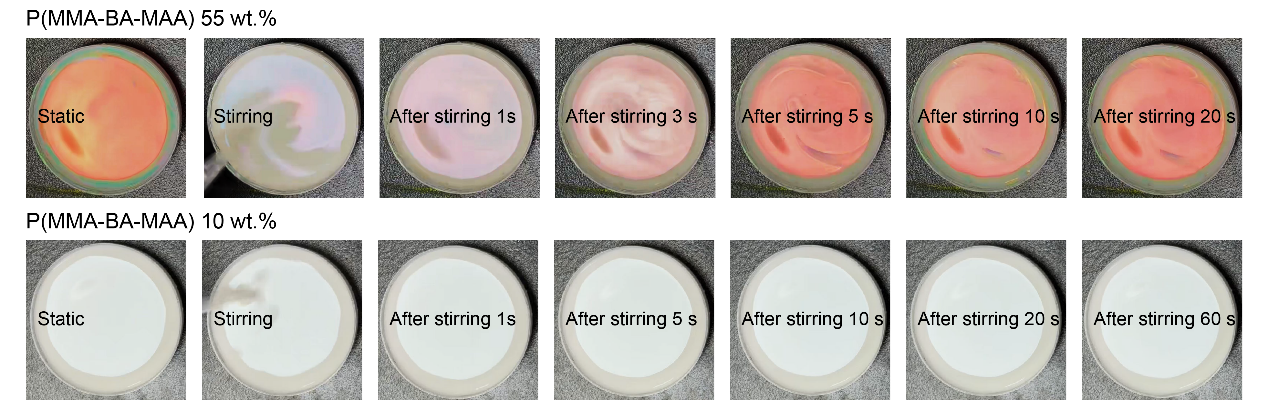


**Fig. S6** Optical images of the formation of colloidal skin during the evaporation-induced self-assembly


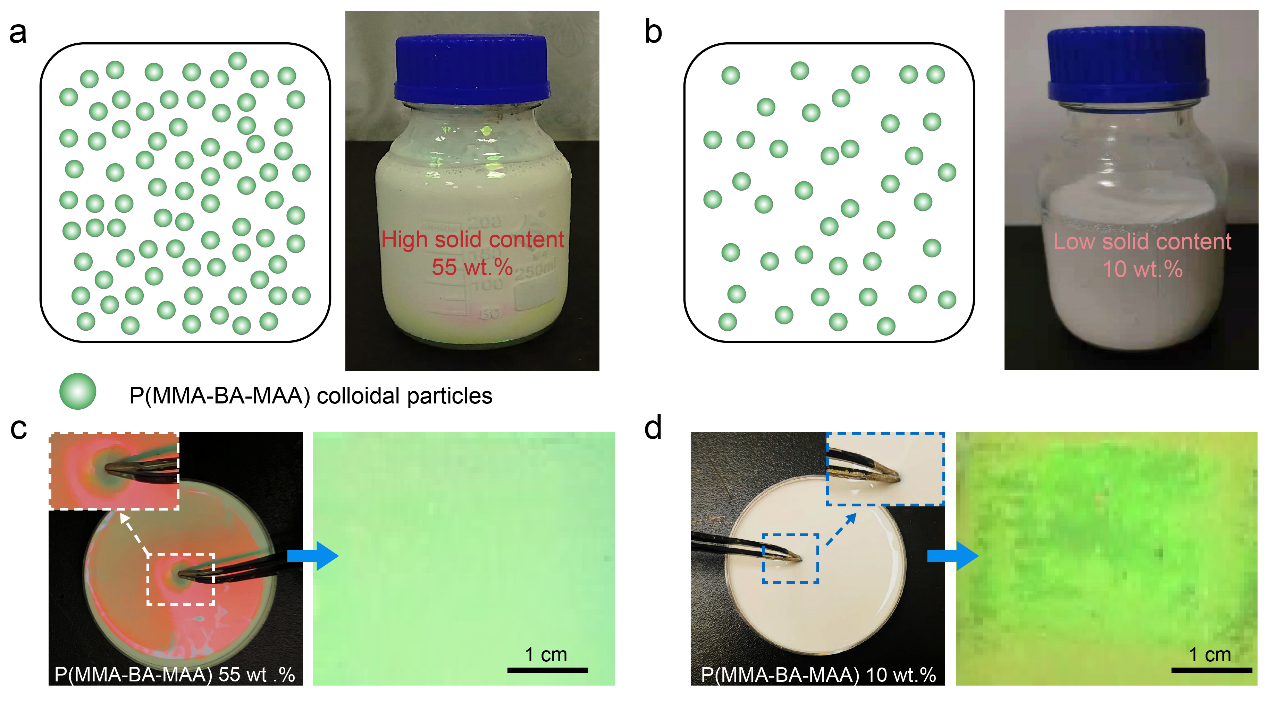


**Fig. S7** Comparison of assembly performance of 10 wt.% latex and 55 wt.% latex. Schematics and photographs of monodispersed P(MMA-BA-MAA) latexes synthesized via **a** our latex polymerization strategy and **b** conventional latex polymerization. Optical photographs (incident light angle: normal direction; illumination types: fluorescent lamp; shooting angle: normal direction) of undisturbed latexes and assembled CPC films of **c** as-synthesized high SC monodispersed P(MMA-BA-MAA) latex (55 wt.%) and **d** traditional low SC monodispersed P(MMA-BA-MAA) latex (10 wt.%). Iridescent “colloid skin” of high SC latex enhances the construction of high-quality CPC film with bright and uniform structural color.


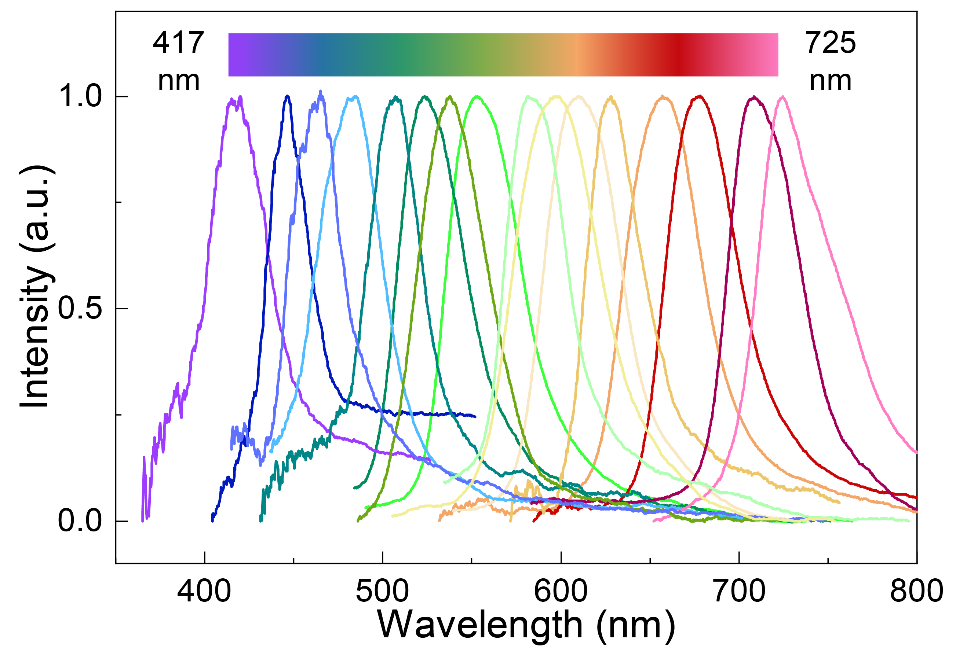


**Fig. S8** Reflection spectrum of photonic metamaterial obtained from high SC P(MMA-BA-MAA) monodispersed latexes covering the entire visible spectrum


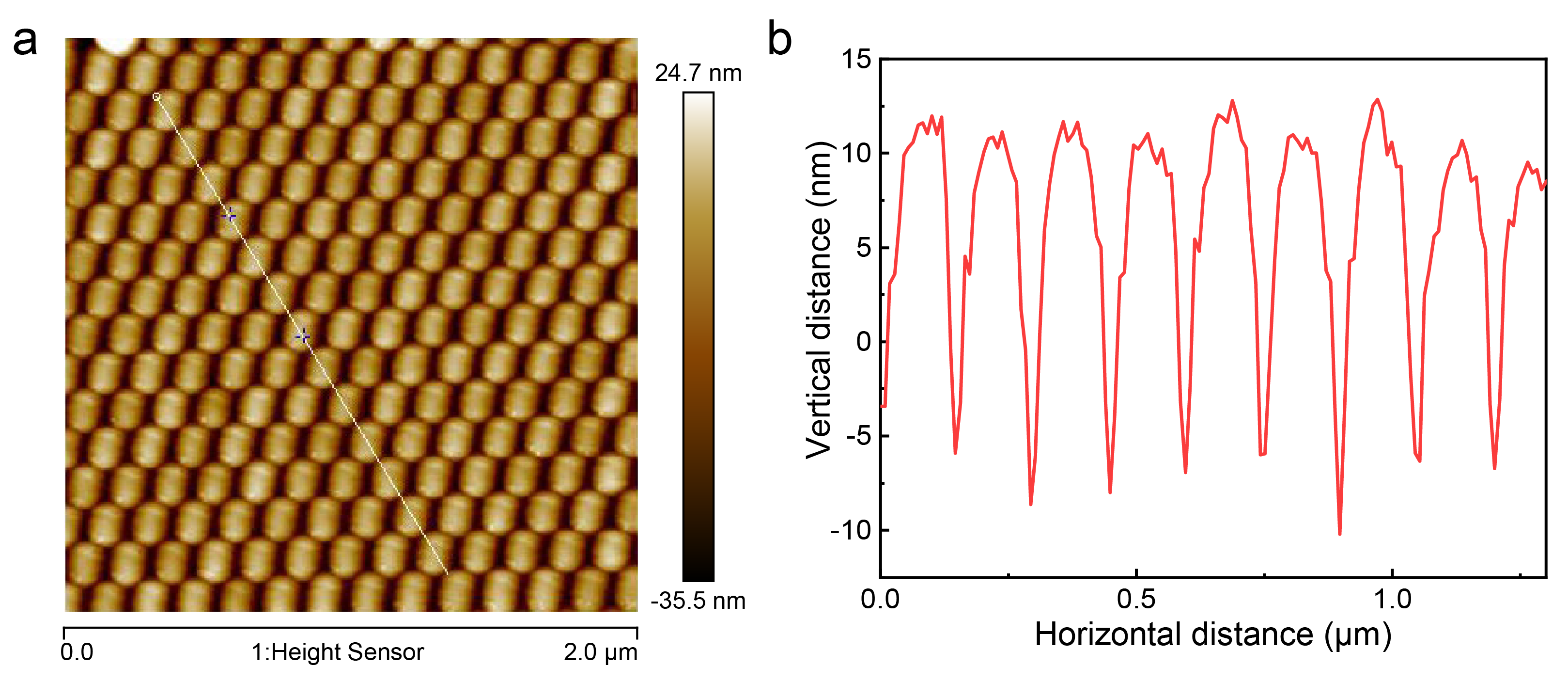


**Fig. S9** Typical 2D atomic force microscopy (AFM) diagram and longitudinal height distribution of the surface of photonic metamaterial constructed by high SC monodispersed P(MMA-BA-MAA) latex


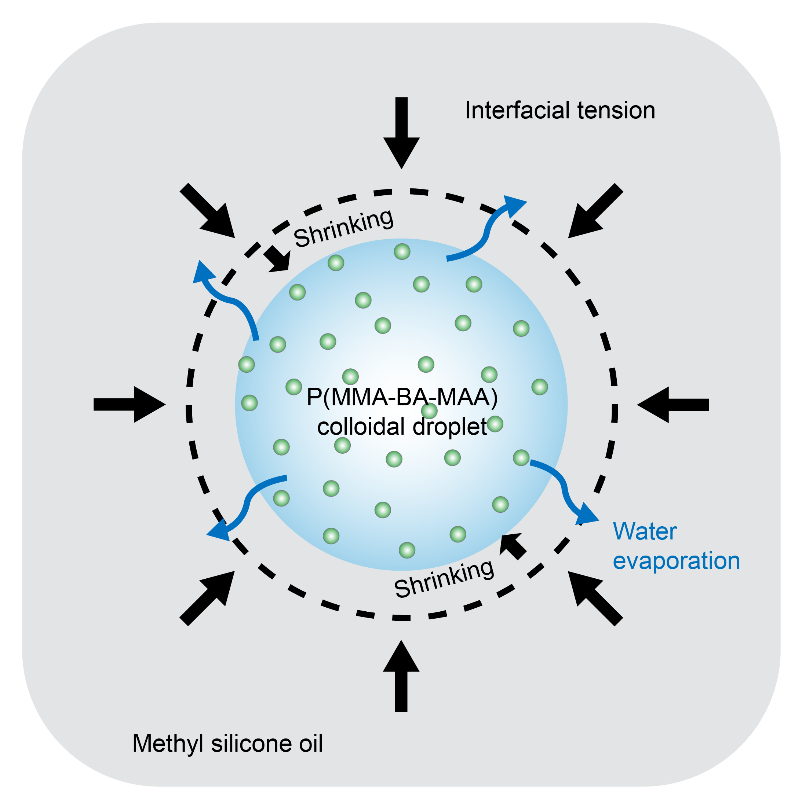


**Fig. S10** Schematic drawing of the levitating droplet of P(MMA-BA-MAA) colloidal latex in methyl silicone oil


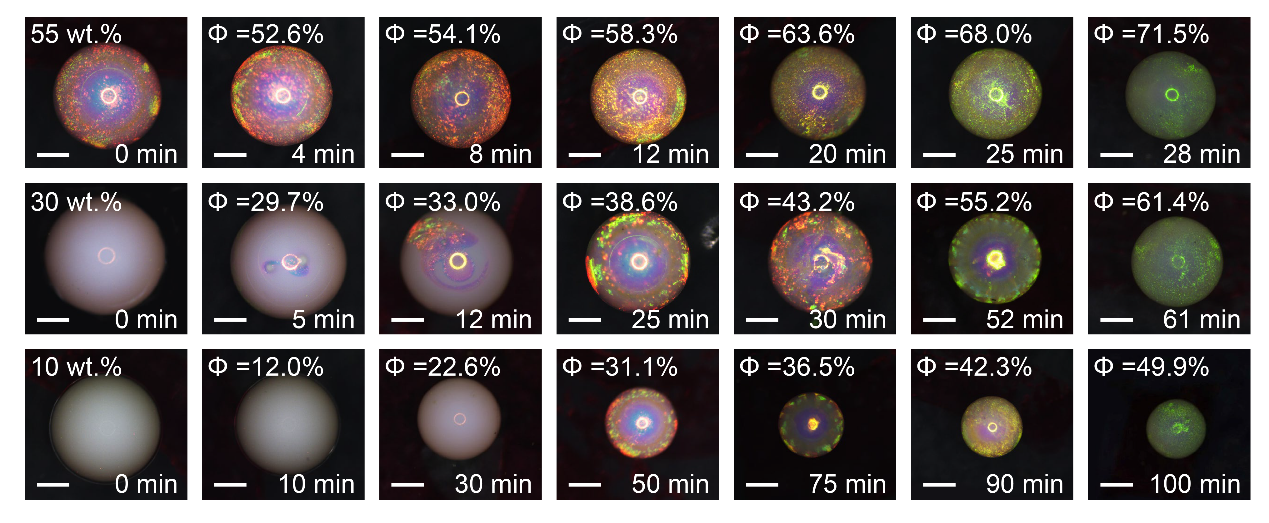


**Fig. S11** Evaporation-induced self-assembly of individual levitating droplets and high-crystallinity photonic metamaterial formation. Temporal evolution of individual levitating droplets with different initial particle concentration 55 wt.% (top row), 30 wt.% (middle row), and 10 wt.% (bottom row) upon solvent evaporation. The 55 wt.% monodispersed latex is outperformed than that of 10 wt.% and 30 wt.% monodispersed latex in assembly efficiency and crystallinity, resulting in high-quality CPC. Scale bar: 200 µm


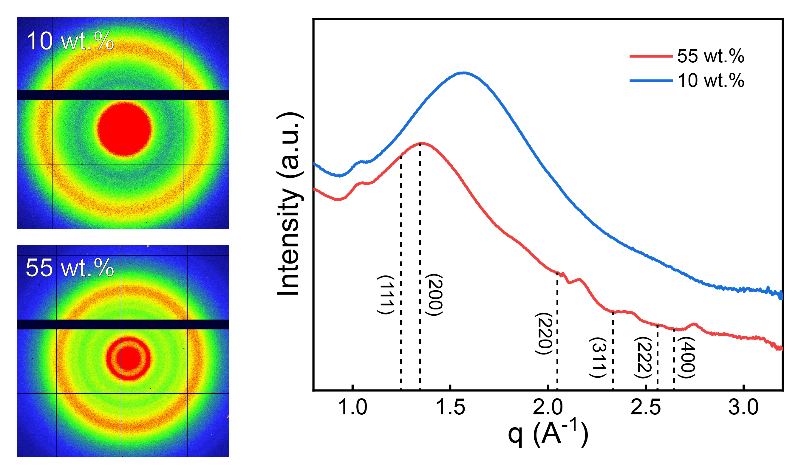


**Fig. S12** 1D SAXS intensity profiles and 2D patterns of CPCs with 55 wt.% and 10 wt.% initial particle concentrations, revealing lattice structure


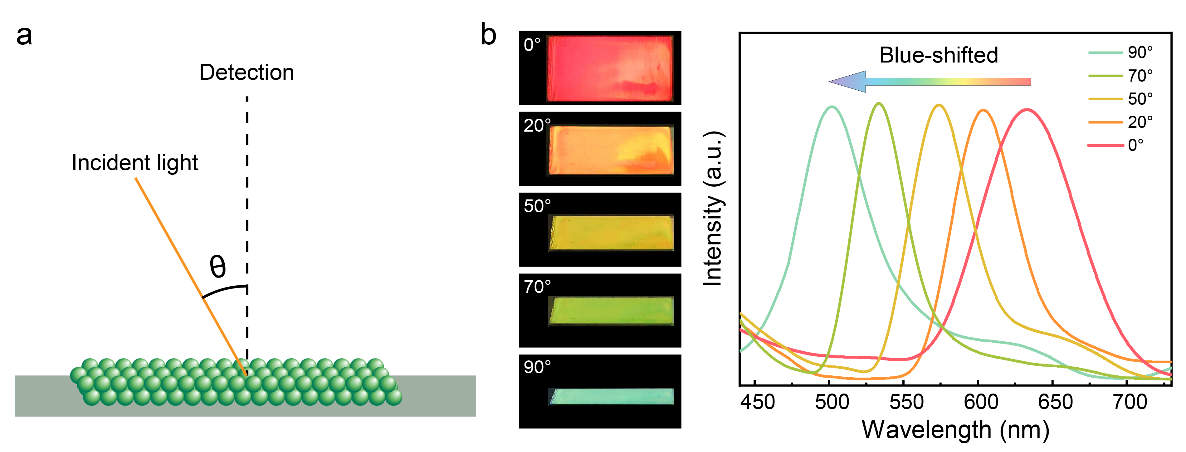


**Fig. S13 a** Schematic illustration of the angle of incidence light (*θ*). **b** Dependence of reflected wavelength on incidence angle. Left is the corresponding optical photograph


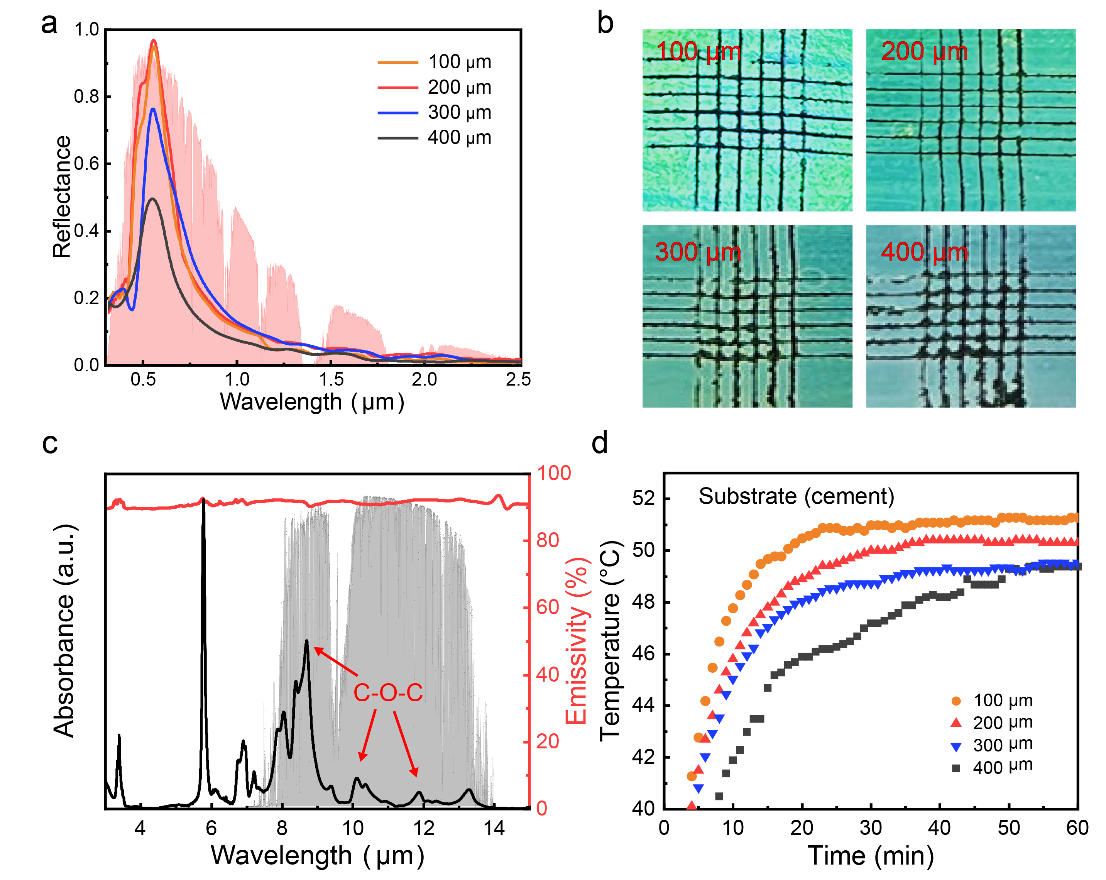


**Fig. S14** Passive cooling performance for the high-crystallinity photonic metamaterial. **a** Reflectivity of the individual CPCs as a function of the coating thickness. **b** Optical images of CPC coating with varying thickness (100-400 μm) after the adhesion test. **c** Absorbance and emissivity spectra of the high-crystallinity CPCs. **d** Temperature measurements of the high-crystallinity CPC samples with different thicknesses under 1000 W/m^2^ simulated solar radiation


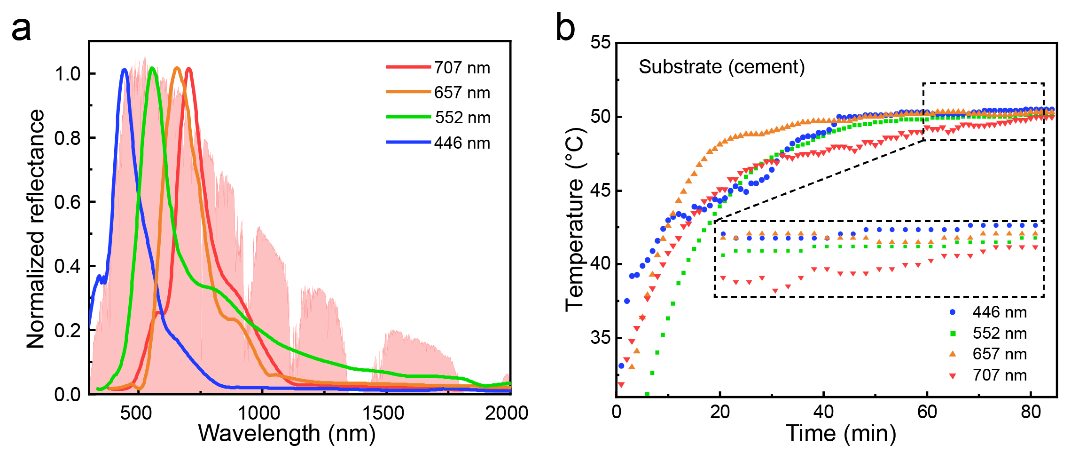


**Fig. S15** **a** Normalized reflection spectrum for the high-crystallinity CPCs with different structural colors. **b** Temperature measurements of the high-crystallinity CPC samples with different structural colors under 1000 W/m^2^ simulated solar radiation


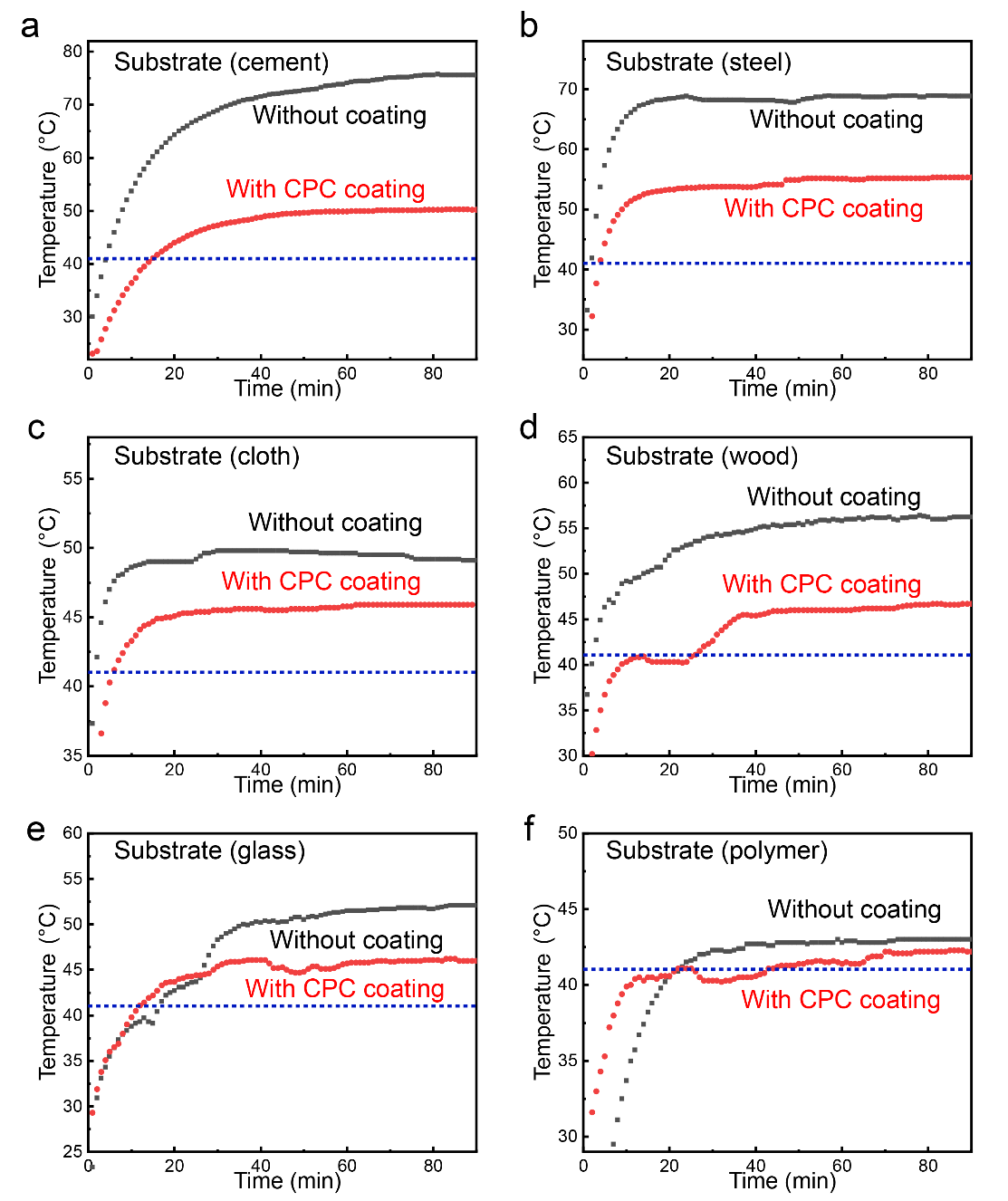


**Fig. S16** Temperature tracking for **a** cement, **b** steel, **c** cloth, **d** wood, **e** glass, and **f** PMMA polymer substrate and the corresponding substrate with high-crystallinity CPC coating under 1000 W/m^2^ simulated solar radiation. Blue dotted line indicates the temperature of control sample (41 ℃)


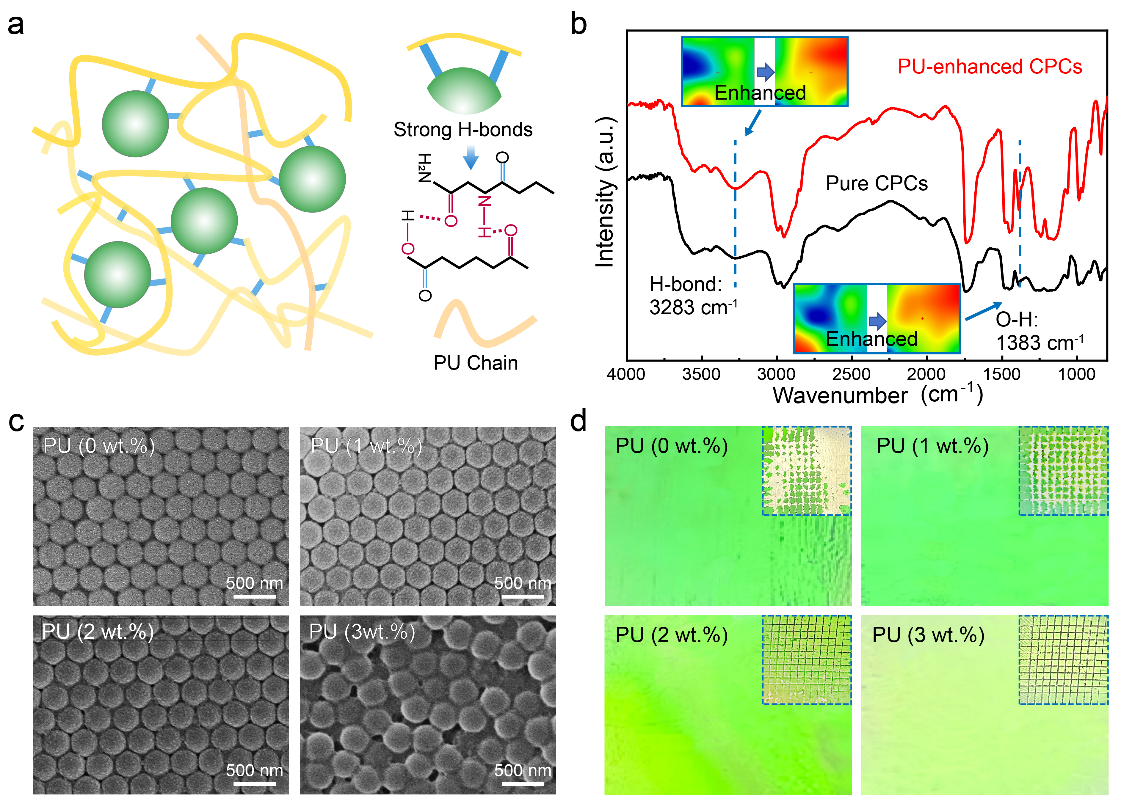


**Fig. S17** Robust CPC coating design. **a** Illustration of hydrogen bond enhancing mechanism of PU for improving mechanical stability of CPC coating. **b** FT-IR spectra of pure CPCs and PU-enhanced CPCs, and the corresponding micro-IR images of characteristic peaks. **c** SEM images of CPC coating doped with different amounts of PU. **d** Optical photographs of CPC coating doped with different amounts of PU. Inset: results of cross-cut adhesion test


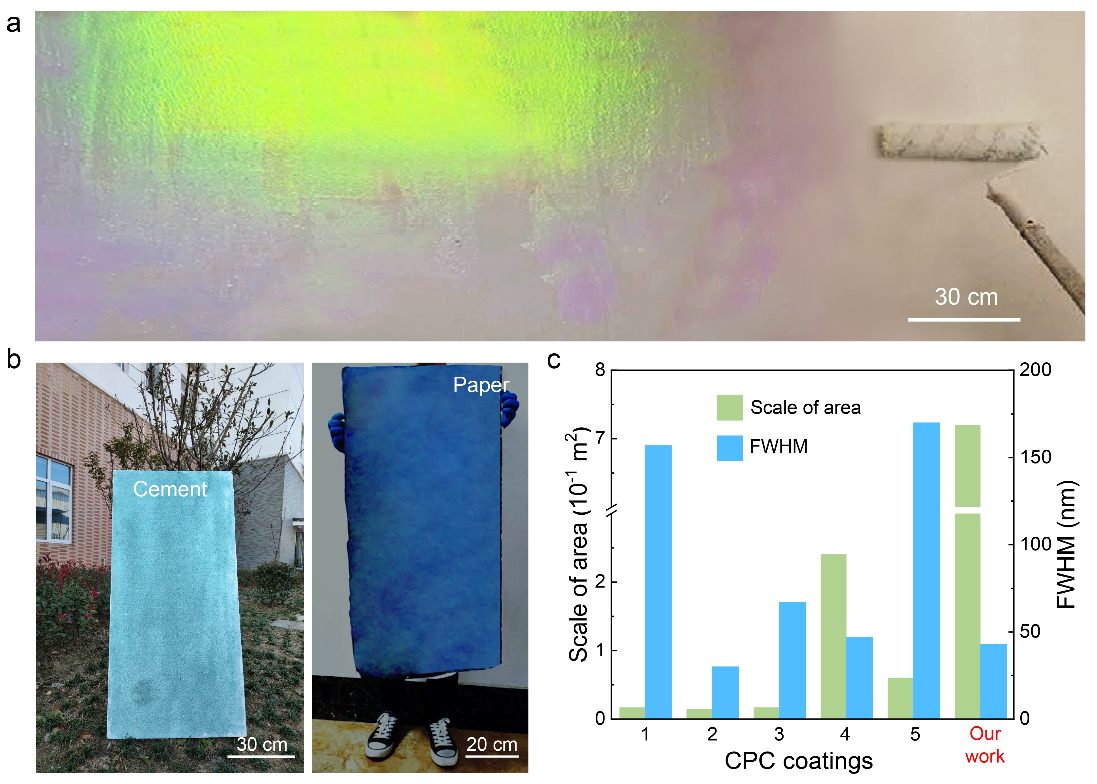


**Fig. S18 a** Photograph showing (incident light angle: normal direction; illumination types: fluorescent lamp; shooting angle: normal direction) the colored PDRC coating on building external wall produced via a roll coating at a speed of ~0.2 m^2^/min. **b** Large-scale CPC coating can be roller-coated on building cement (60 cm by 120 cm), and spray-coated on paper (50 cm by 150 cm). **c** 55 wt.% SC CPC coating compared with other large-scale CPC coatings, highlighting the unique benefit of the high SC CPC coating of being simultaneously in scale and optical properties (details in Table S5).


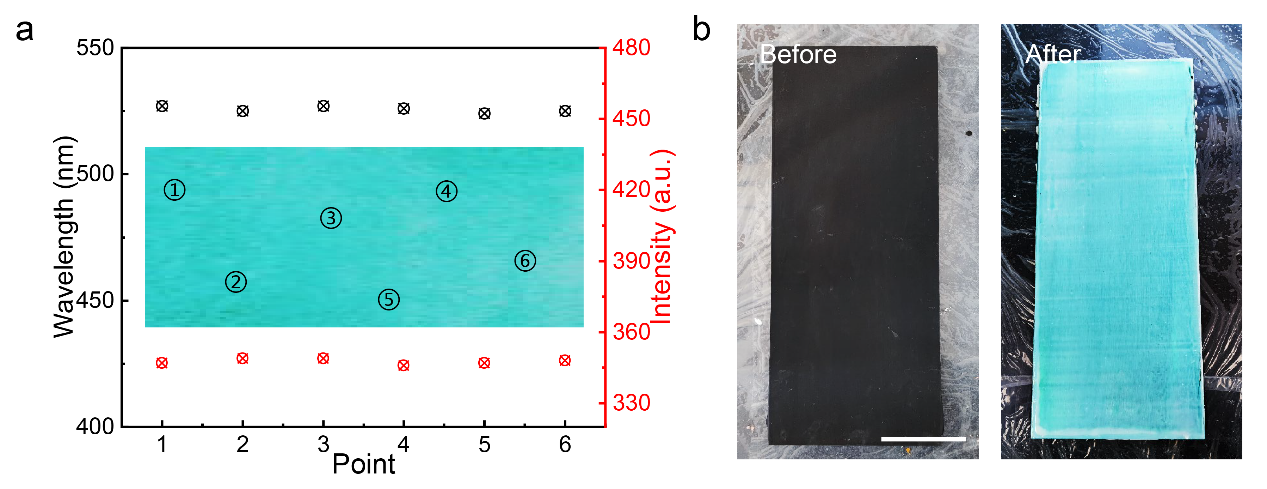


**Fig. S19 a** Optical property characterization of the large-scale CPC coating at different positions. Scale bar: 10 cm. **b** Photographs of fibred cement plate before and after coating with CPCs. Scale bar: 10 cm


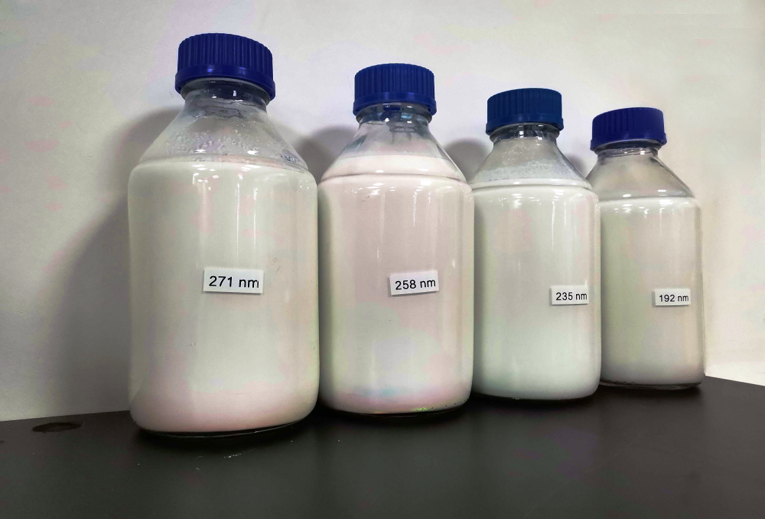


**Fig. S20** Photo showing the 1-L-volume 55 wt.% SC monodispersed P(MMA-BA-MAA) latex that produced in an amplified reaction.


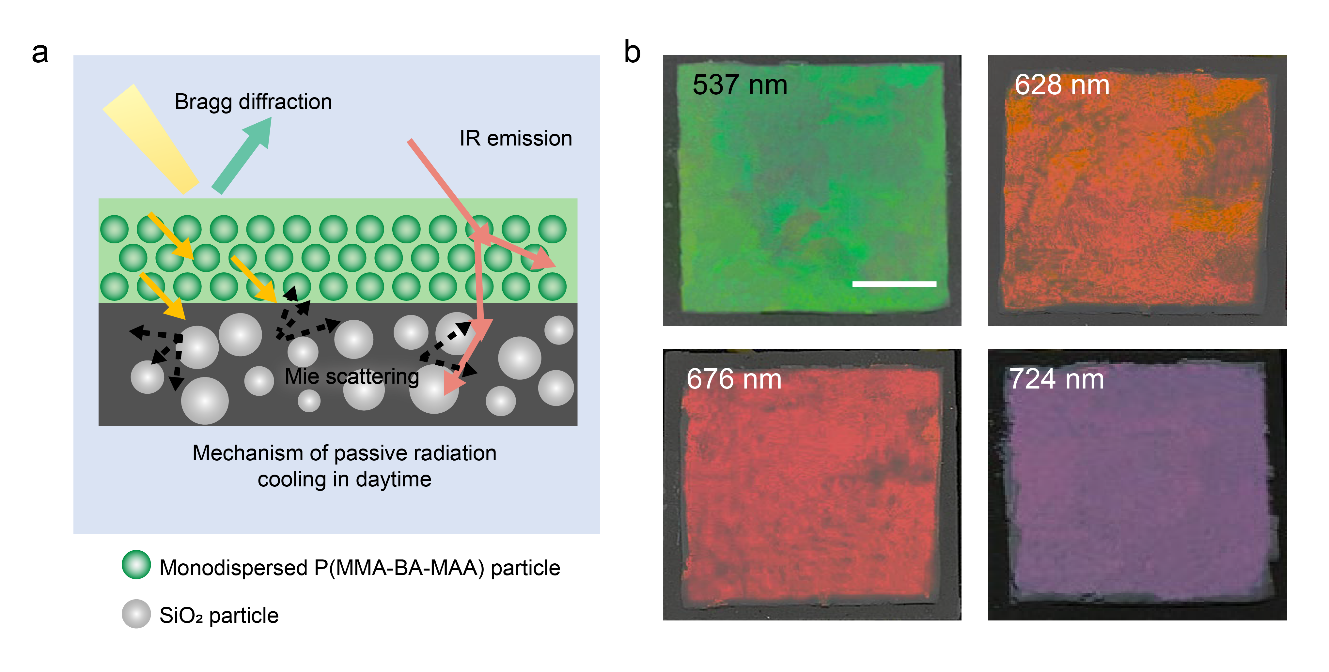


**Fig. S21 a** Schematic of the hierarchical PDRC coating, where the coating reflects the incident solar irradiance and is highly infrared emissive. **b** Photographs of hierarchical PDRC coating with green, orange, red, and infrared structural colors constructed from CPC upper layers with reflection peaks of 537 nm, 628 nm, 676 nm, and 724 nm, the bottom layer was controlled as glass-polymer coating containing 43 wt.% SiO_2_ particles. Scale bar: 3 cm


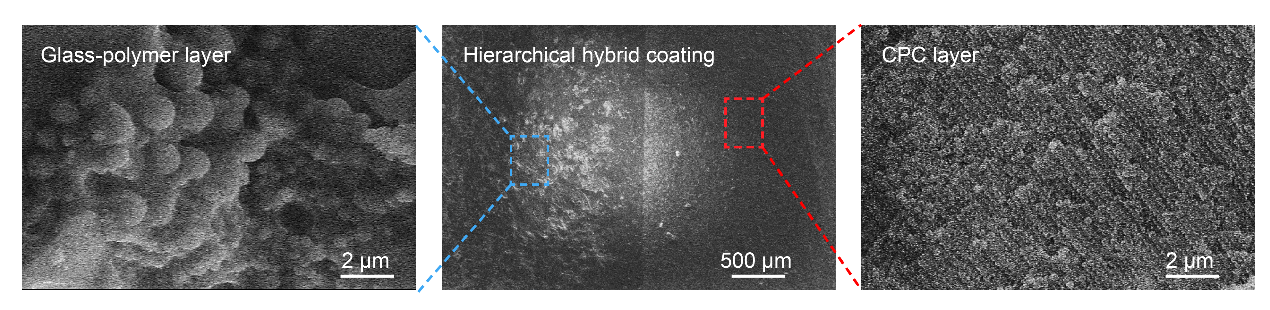


**Fig. S22** SEM micrographs of the hierarchical PDRC coating. The PDRC coating with a CPC upper layer directly derived from high SC latex (right) and a glass-polymer bottom layer (left). SiO_2_ particles with different sizes randomly distributed into PU as the bottom layer, providing enhanced Mie scattering and IR emission. And long-range ordered lattice structure throughout the upper layer to achieve selective solar reflection.


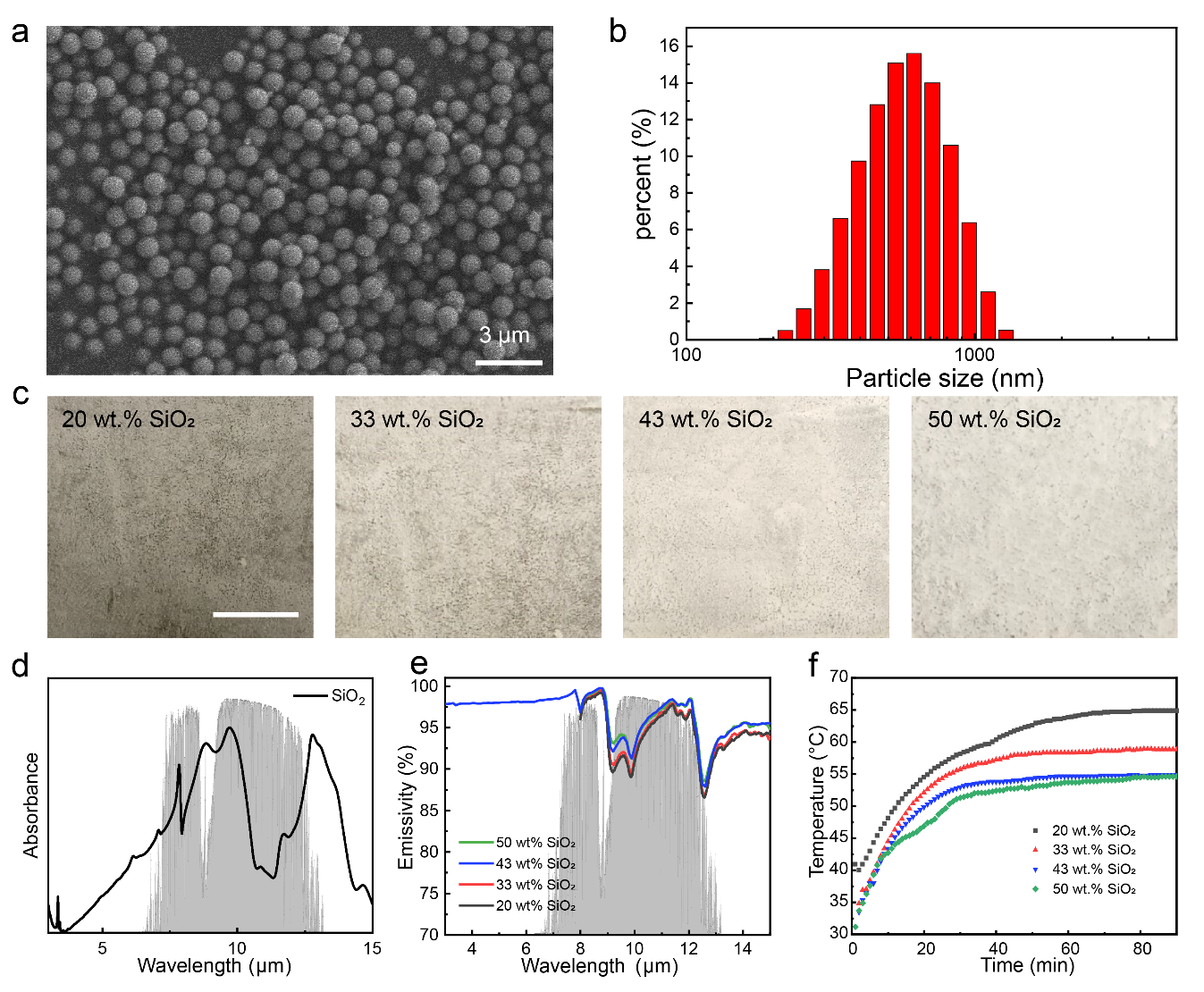


**Fig. S23 a** SEM image and **b** size distribution of SiO_2_ nanoparticles with particle sizes ranging from 200 to 1200 nm. The SiO_2_ nanoparticles could achieve broadband emissivity by accessing the high-order Frohlich resonances, and produce the scattering peaks covered the Vis-NIR band using the collective effect of multiple Mie resonances. **c** Photographs of glass-polymer coating, in which SiO_2_ particles with concentrations of 20 wt.%, 33 wt.%, 43 wt.%, and 50 wt.%, respectively. Scale bar: 3 cm. **d** Absorbance spectrum of SiO_2_ particles measured with FT-IR spectroscopy. **e** Measured emissivity spectra of the glass-polymer coating as a function of the SiO_2_ particles concentration. **f** Surface temperature measurements of the glass-polymer coating samples with different SiO_2_ particle concentrations (substrate: cement)


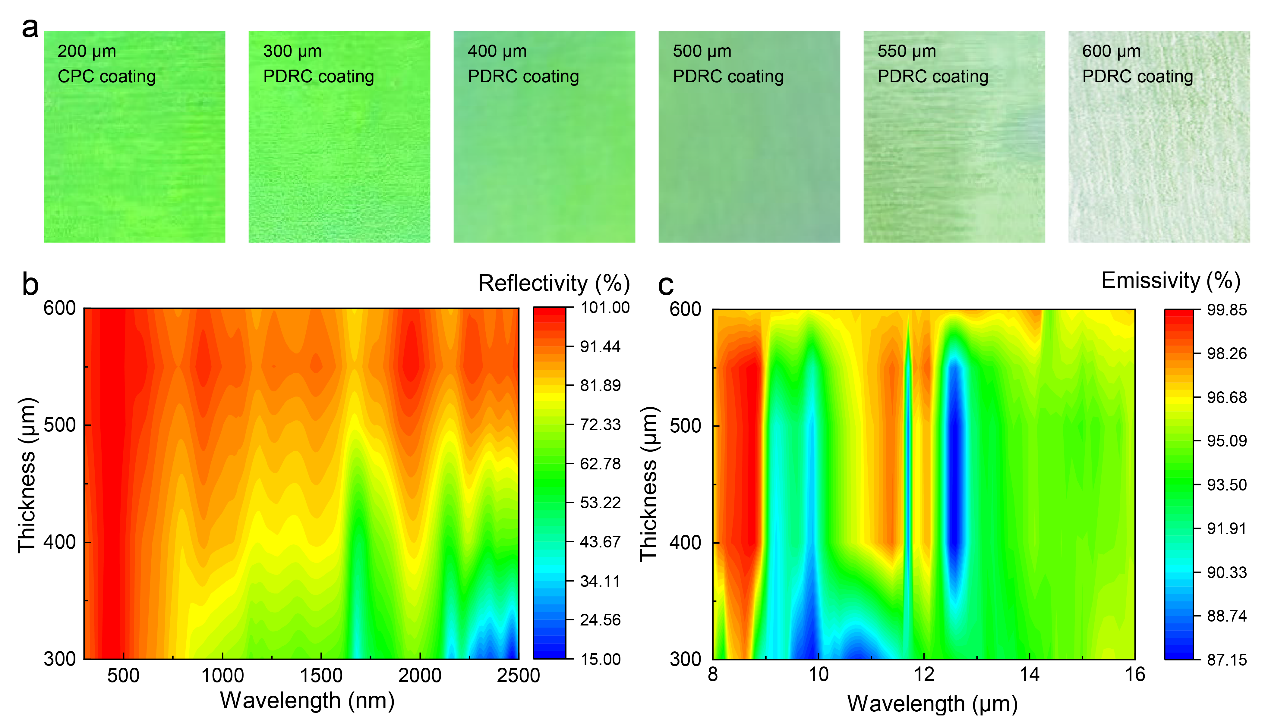


**Fig. S24 a** Optical images of pure CPC coating and colored PDRC coatings with different thicknesses. **b** Solar reflectance and **c** IR emission spectra of the hierarchical PDRC coating as a function of effective thickness


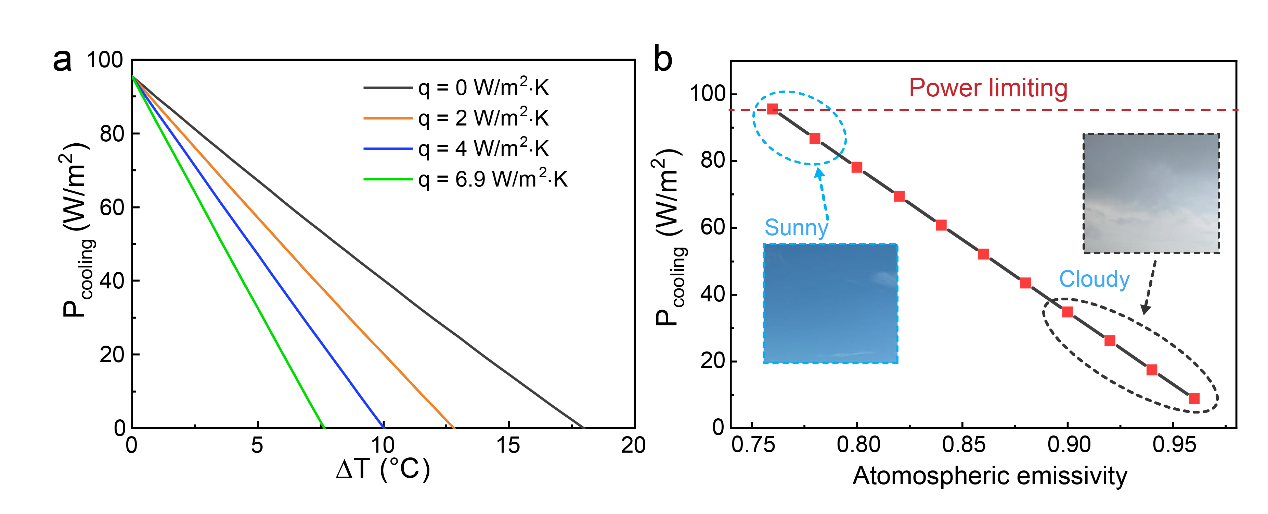


**Fig. S25 a** Calculated theoretical cooling power of the PDRC coating (0.76 atmospheric emissivity) at different *q* values, *T_atm_* = 25 ℃. **b** Theoretical cooling power of the colored PDRC coating as function of atmospheric emissivity, *T_atm_* = *Ts* = 25 ℃


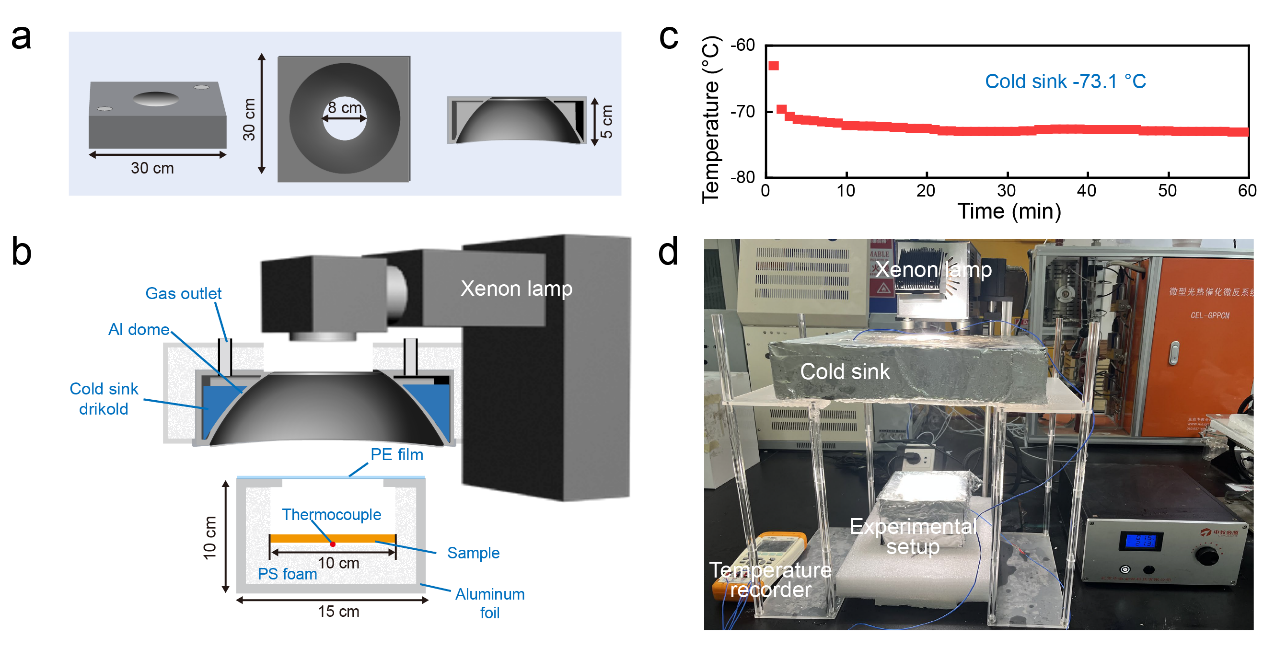


**Fig. S26** Indoor radiative cooling characterization using drikold as the cold sink. **a** Structural diagram of aluminum dome. **b** Schematic illustration of the indoor thermal measurement system. A drikold-cooled aluminum dome acts as the cold outer space, while a solar simulator provides the solar radiation for the samples. **c** Temperature tracking of the cold sink. **d** Photograph of the setup for characterizing indoor PDRC


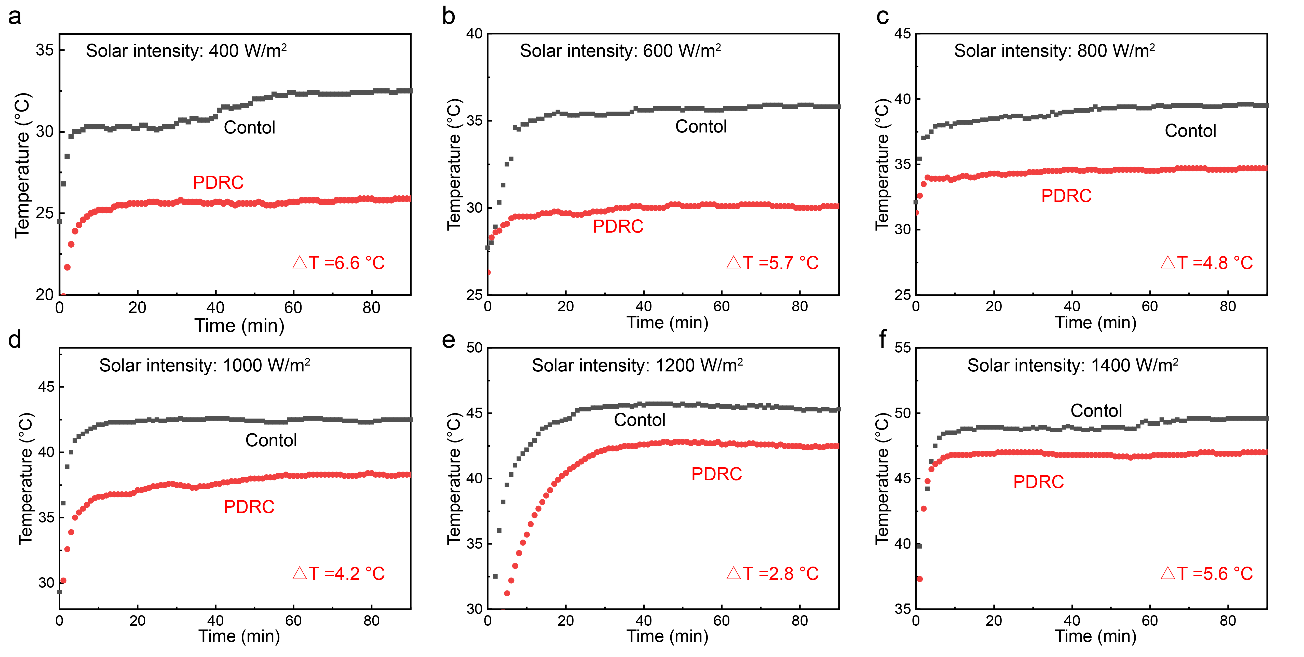


**Fig. S27** First group of temperature tracking experiments for PDRC coating and control sample under **a** 400 W/m^2^, **b** 600 W/m^2^, **c** 800 W/m^2^, **d** 1000 W/m^2^, **e** 1200 W/m^2^, and **f** 1400 W/m^2^ simulated solar radiation

**
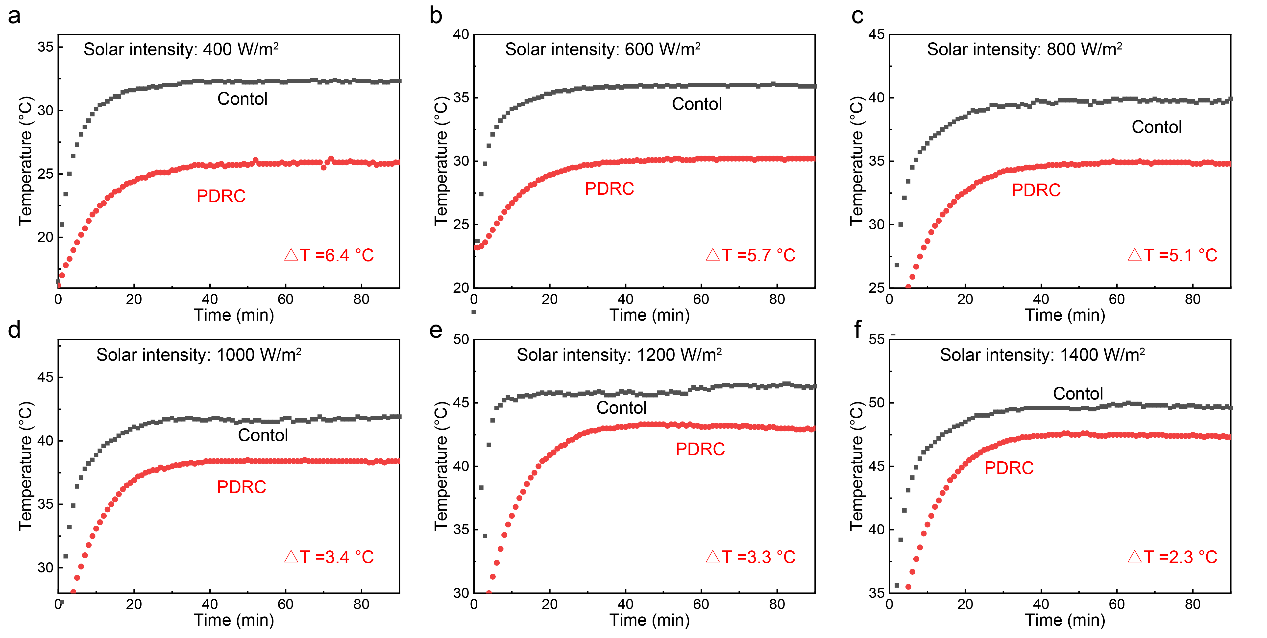
**

**Fig. S28** Second group of temperature tracking experiments for PDRC coating and control sample under **a** 400 W/m^2^, **b** 600 W/m^2^, **c** 800 W/m^2^, **d** 1000 W/m^2^, **e** 1200 W/m^2^, and **f** 1400 W/m^2^ simulated solar radiation

**
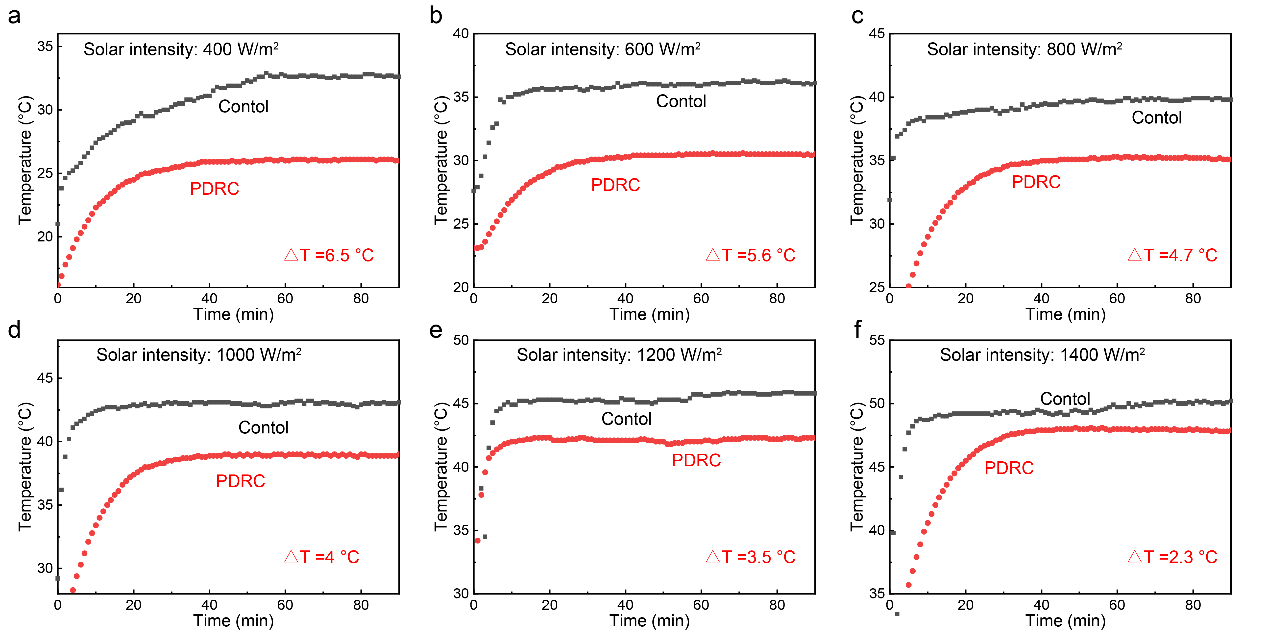
**

**Fig. S29** Third group of temperature tracking experiments for PDRC coating and control sample under **a** 400 W/m^2^, **b** 600 W/m^2^, **c** 800 W/m^2^, **d** 1000 W/m^2^, **e** 1200 W/m^2^, and **f** 1400 W/m^2^ simulated solar radiation


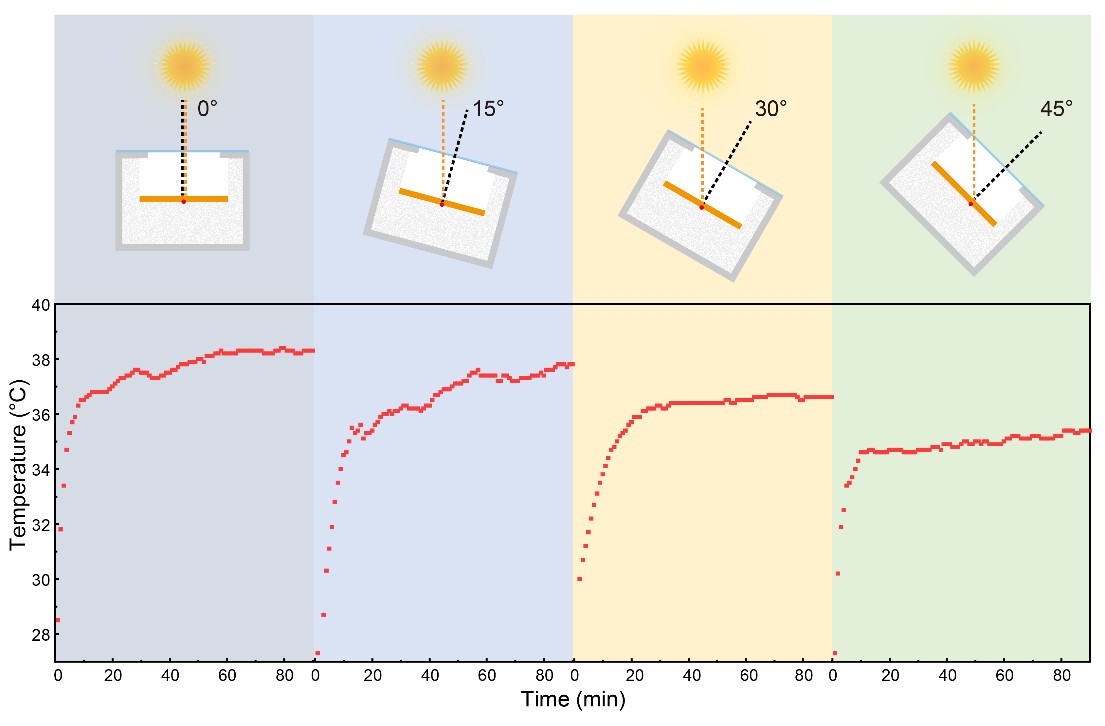


**Fig. S30** Cooling performance evaluation of the PDRC coating under different solar illumination angles

**
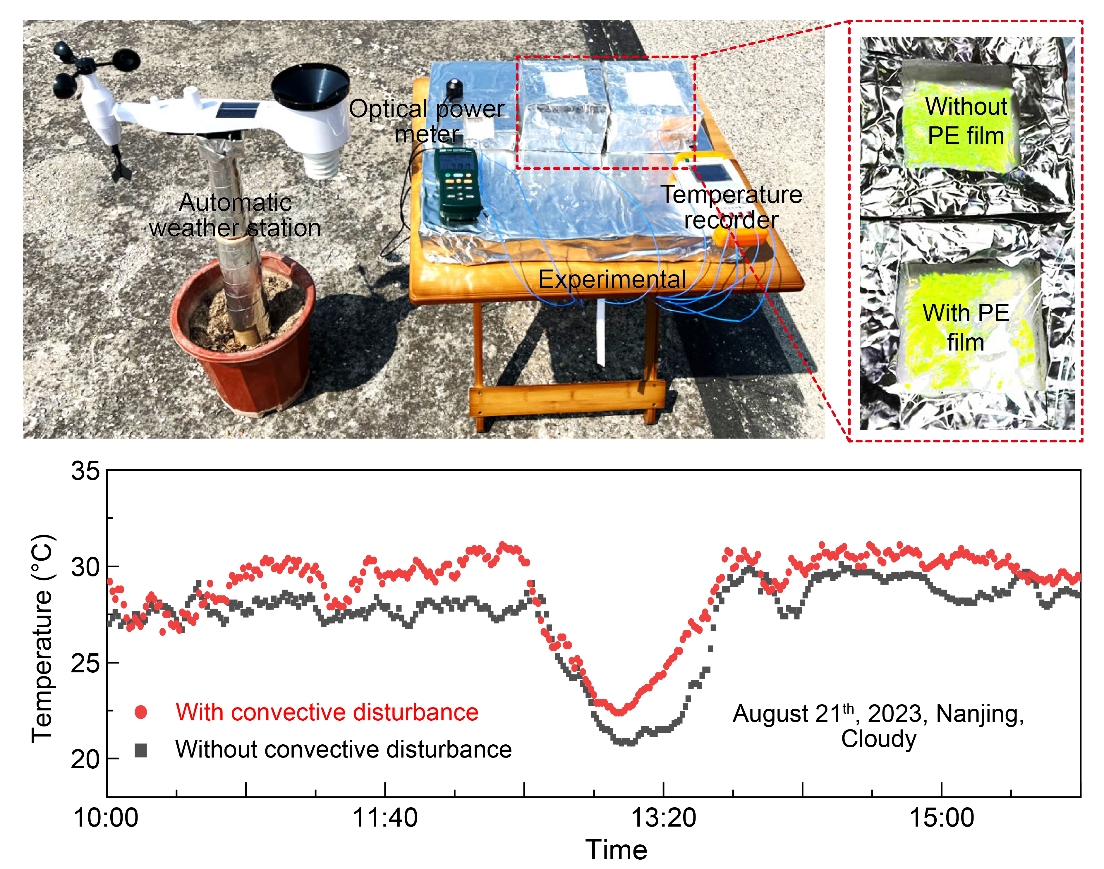
**

**Fig. S31** Photograph of the setup for the outdoor measurements of PDRC performance in Nanjing, China (32^o^6’N, 118^o^40’E). Control experiment without PE shielding was designed to evaluate the utility performance of the PDRC coating in application


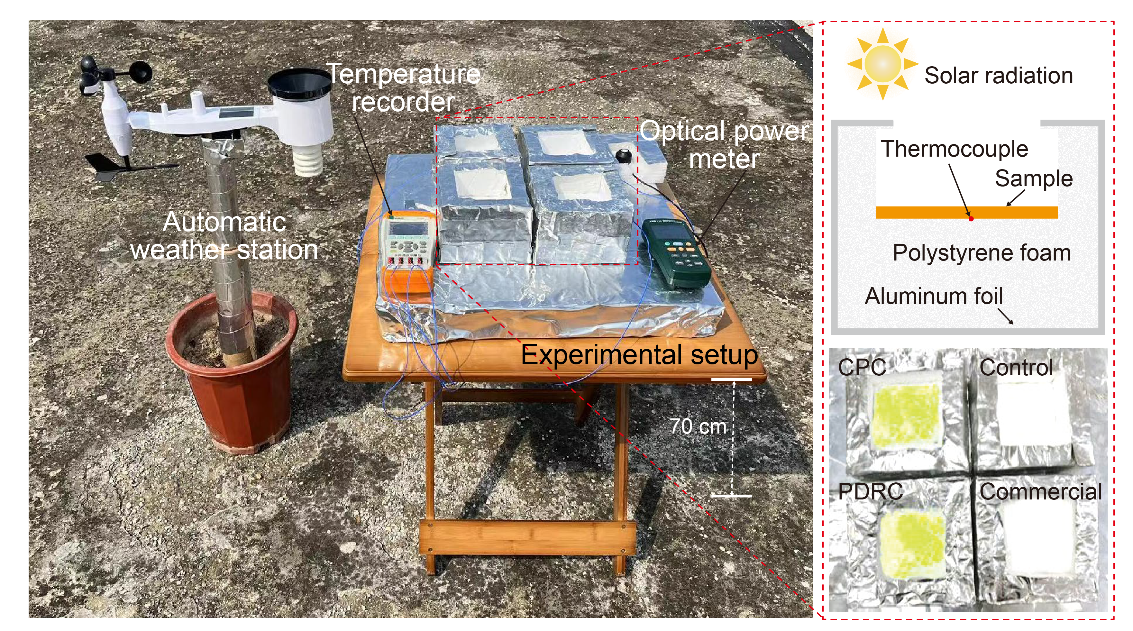


**Fig. S32** Digital Photograph of the setup for the outdoor measurements in Nanjing, China (32^o^6’N, 118^o^40’E) of PDRC performance. Cooling performance of the PDRC coating was compared with CPC coating and the commercial cooling coating


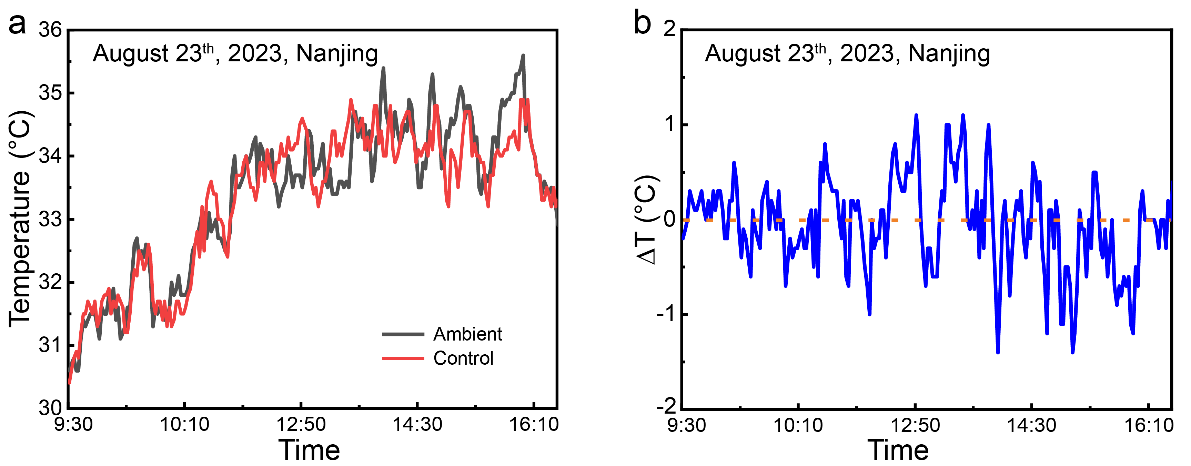


**Fig. S33 a** Temperatures of ambient and control setup in outdoor experiment in Nanjing, China (32^o^6’N, 118^o^40’E). **b** Temperature difference between ambient and control setup


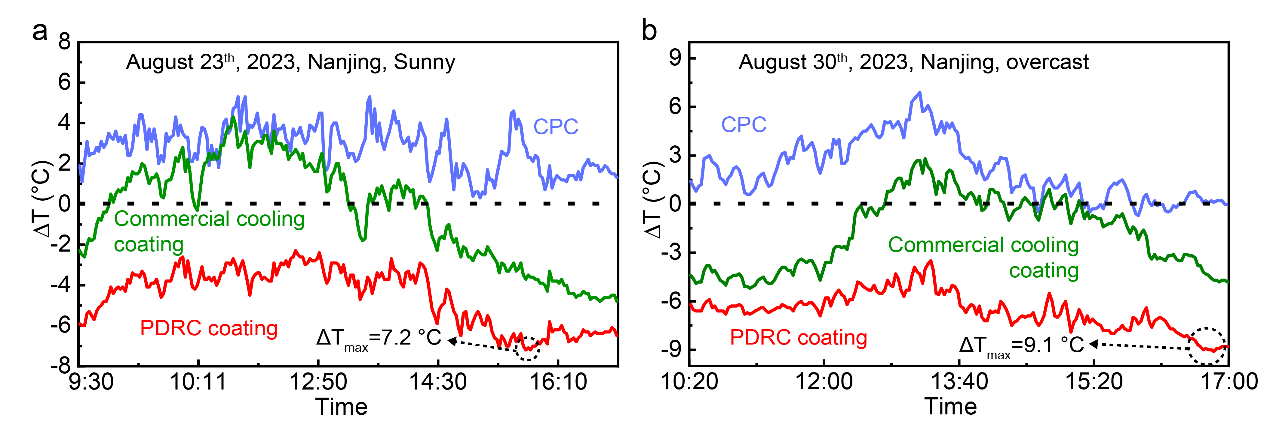


**Fig. S34** Evaluation of cooling performance of commercial cooling coating, CPC coating, and the hierarchical PDRC coating under direct sunlight **a** in sunny (August 23^th^, 2023) and **b** in overcast (August 30^th^, 2023)


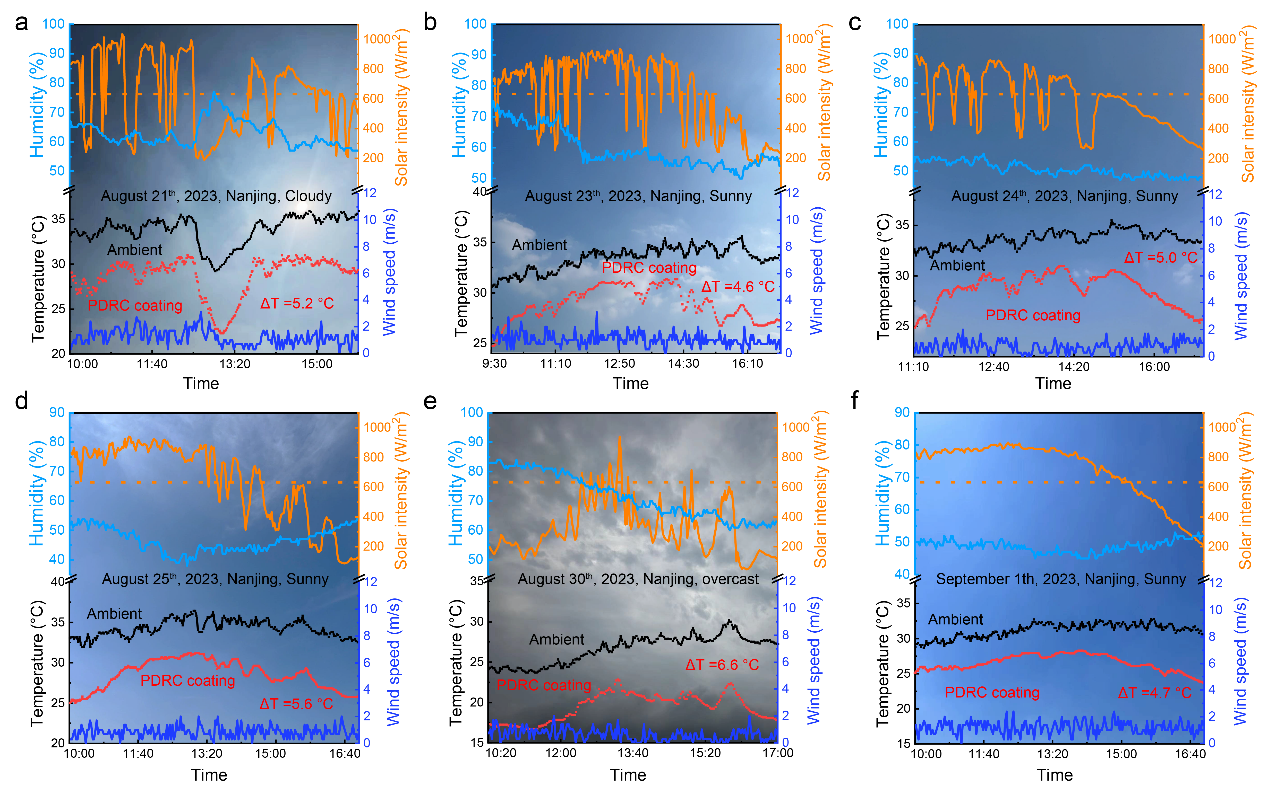


**Fig. S35** Outdoor experiments for the characterization of cooling performance of the PDRC coating. Sub-ambient cooling was achieved in Nanjing in **a** August 21^th^, **b** August 23^th^, **c** August 24^th^, **d** August 25^th^, **e** August 30^th^, and **f** September 1^th^. Orange dotted line indicates the average *I_solar_*


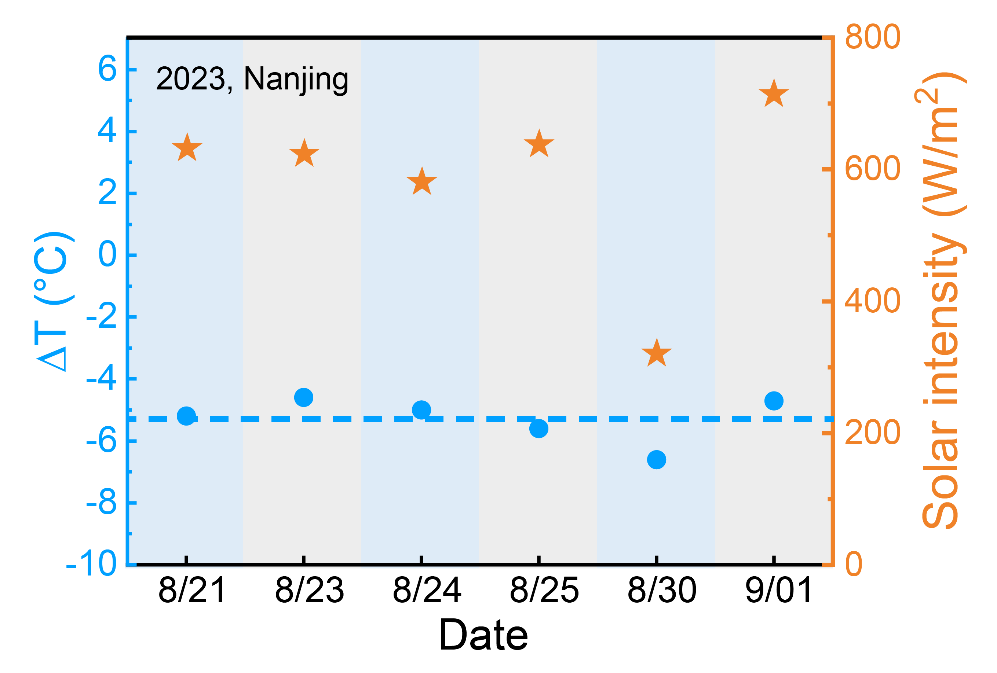


**Fig. S36** Average sub-ambient *ΔT* among all outdoor experiments. And average solar intensity for reference


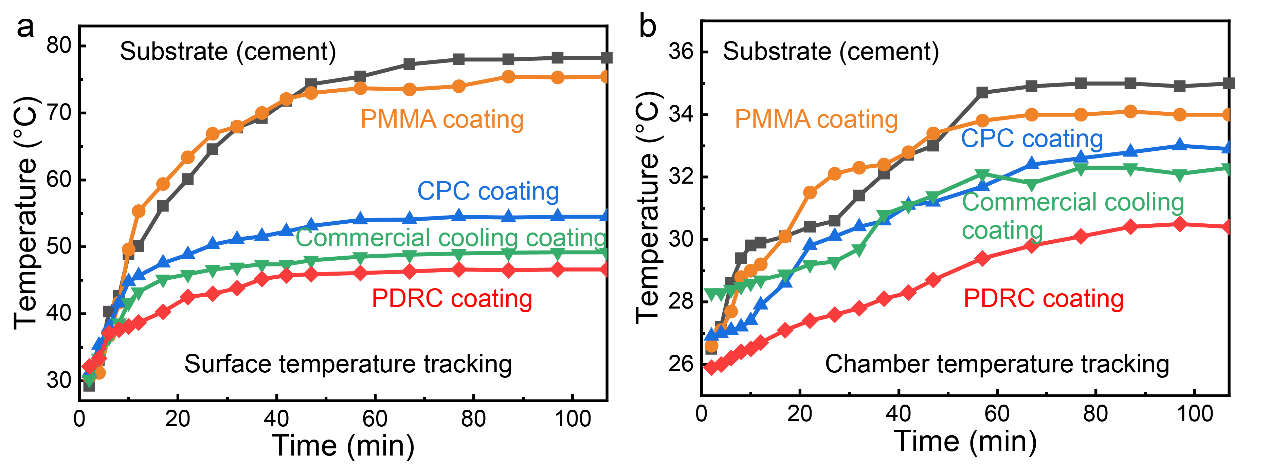


**Fig. S37** Characterization of cooling performance of the hierarchical PDRC coating as building envelopes. **a** Surface temperature tracking and **b** chamber temperature tracking for the building cement covered by different coatings under 1000 W/m^2^ simulated solar radiation


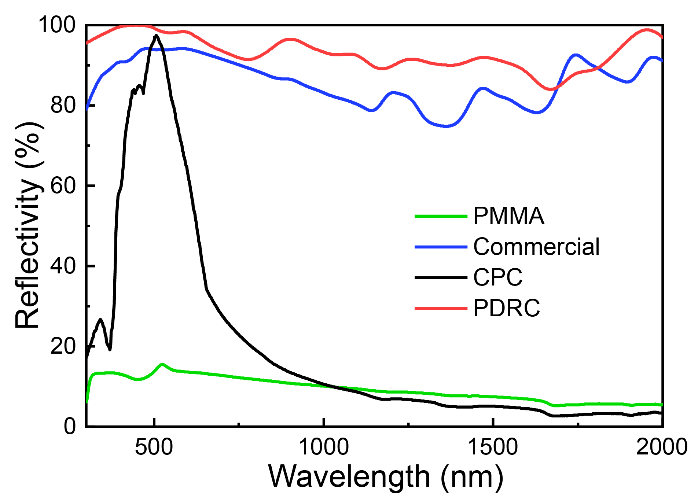


**Fig. S38** Reflectivity spectra of PMMA covering (green curve), commercial cooling coating (blue curve), CPC (black curve), and hierarchical PDRC coating (red curve) from 300 nm to 2000 nm


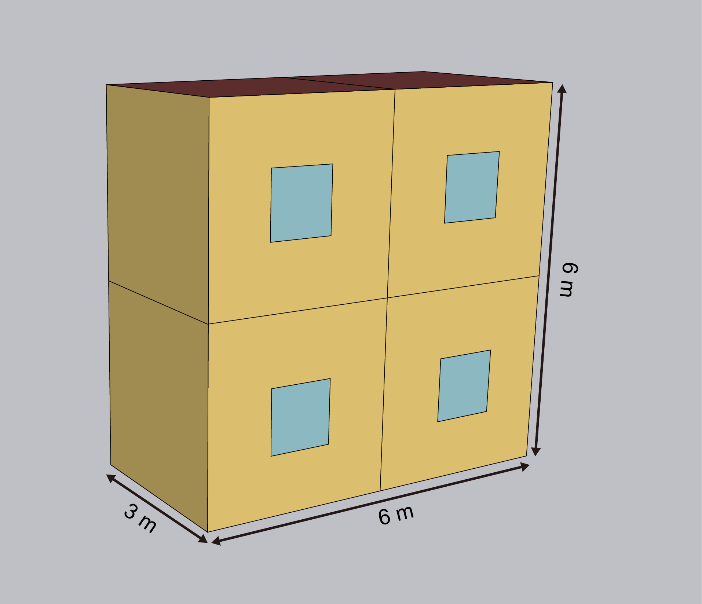


**Fig. S39** Simplified midrise apartment building model for OpenStudio simulations


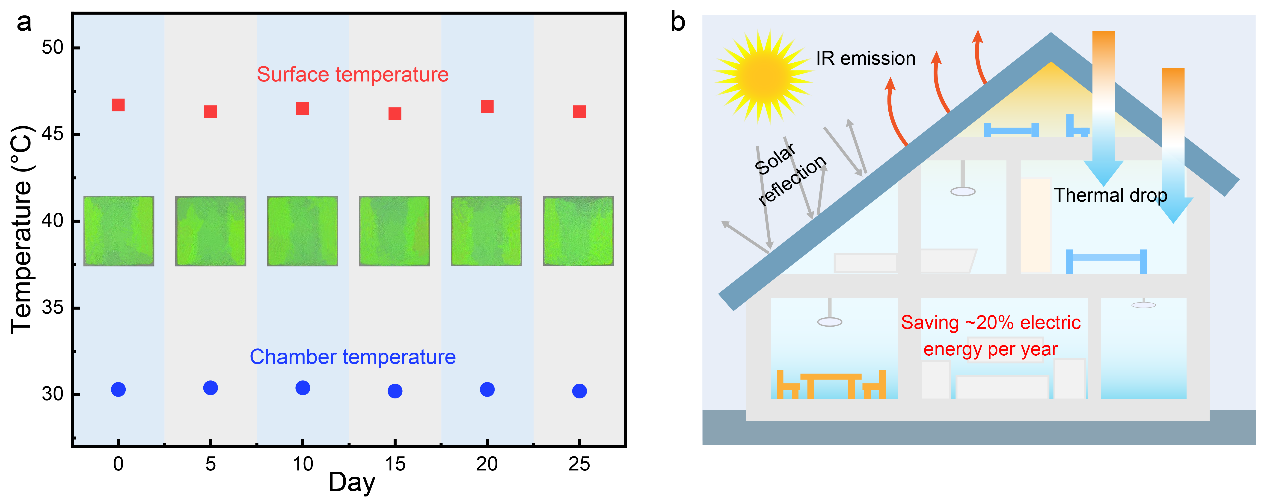


**Fig. S40 a** Relationship between temperature characterization of building envelope samples and days (25 days). Inset: physical maps of the building envelope sample with hierarchical PDRC coating (size: 100 cm^2^) for 25 days. **b** Estimated cooling electrical energy savings percentage per year of the hierarchical PDRC coating used as building envelopes for a typical single-family housing

**
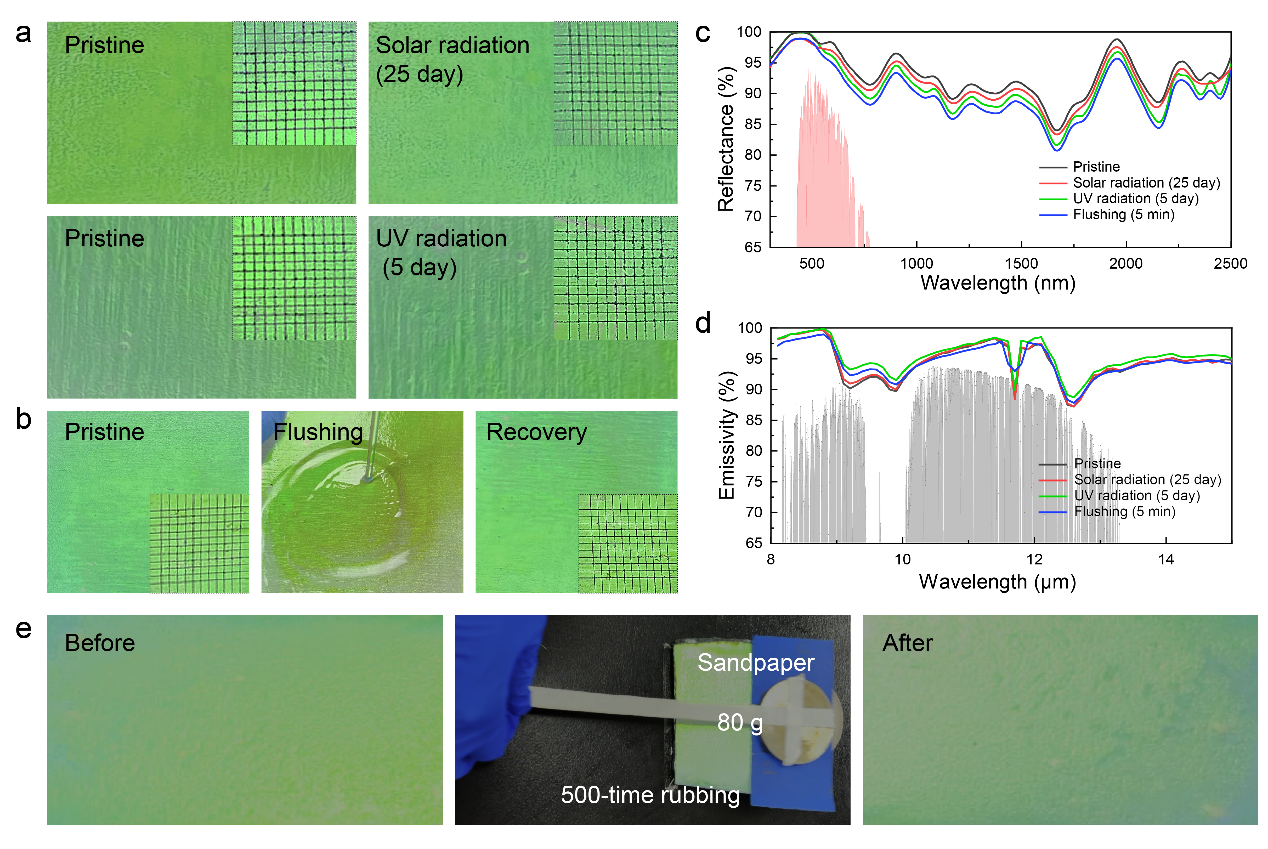
**

**Fig. S41** **a** Optical photographs of the pristine colored PDRC coating and the coating irradiated by simulated solar (1000 W/m^2^) and UV light. Inset: cross-cut adhesion test of coating. **b** Photographs of the colored PDRC coating suffered from water flushing. **c** Reflectivity, and **d** emissivity of the pristine colored PDRC coating and the coating radiated by simulated solar (1000 W/m^2^), UV light, and water flushing. **e** Wear fastness of the colored PDRC coating


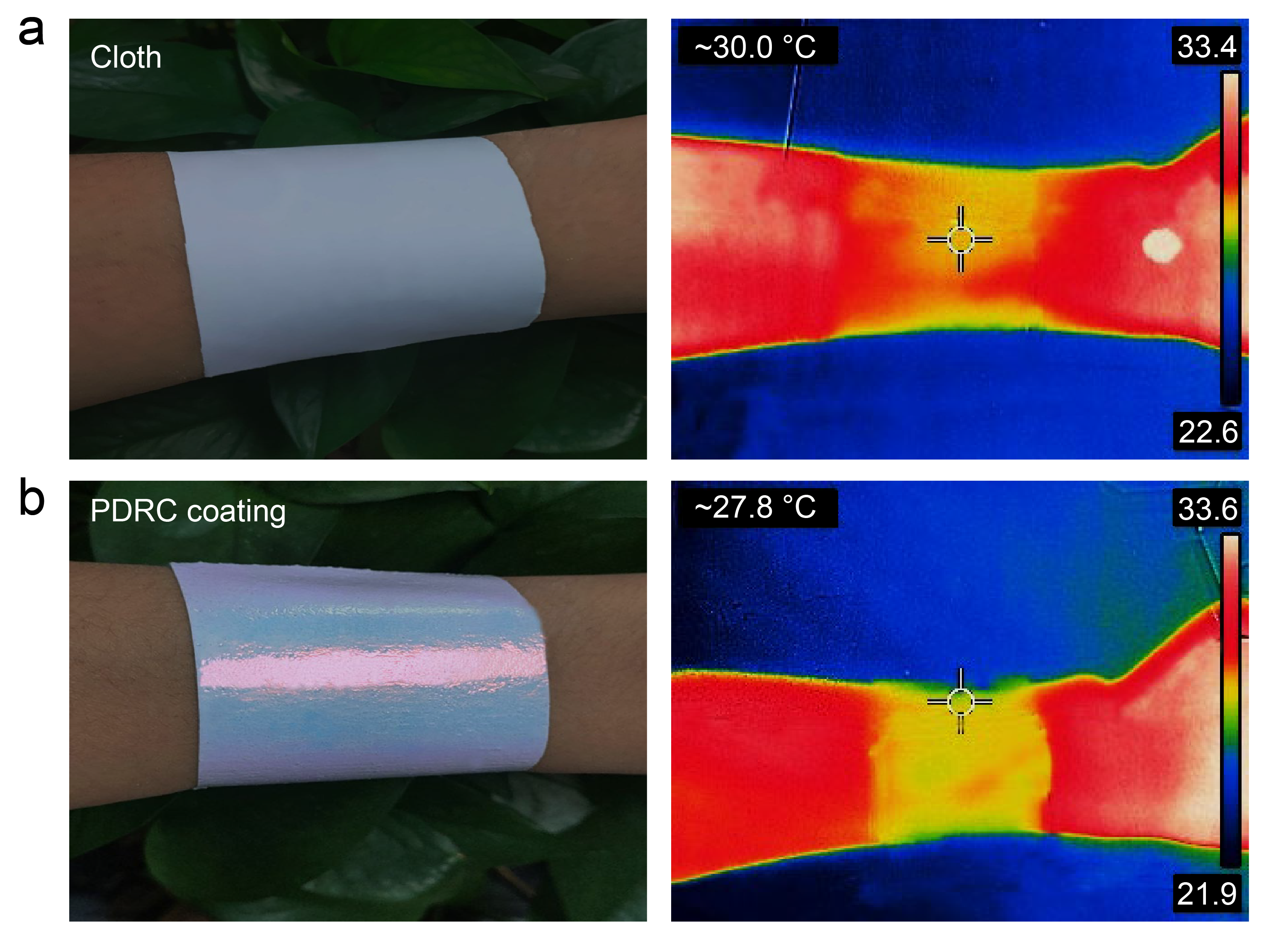


**Fig. S42** Hierarchical PDRC coating applied to the human body for cooling


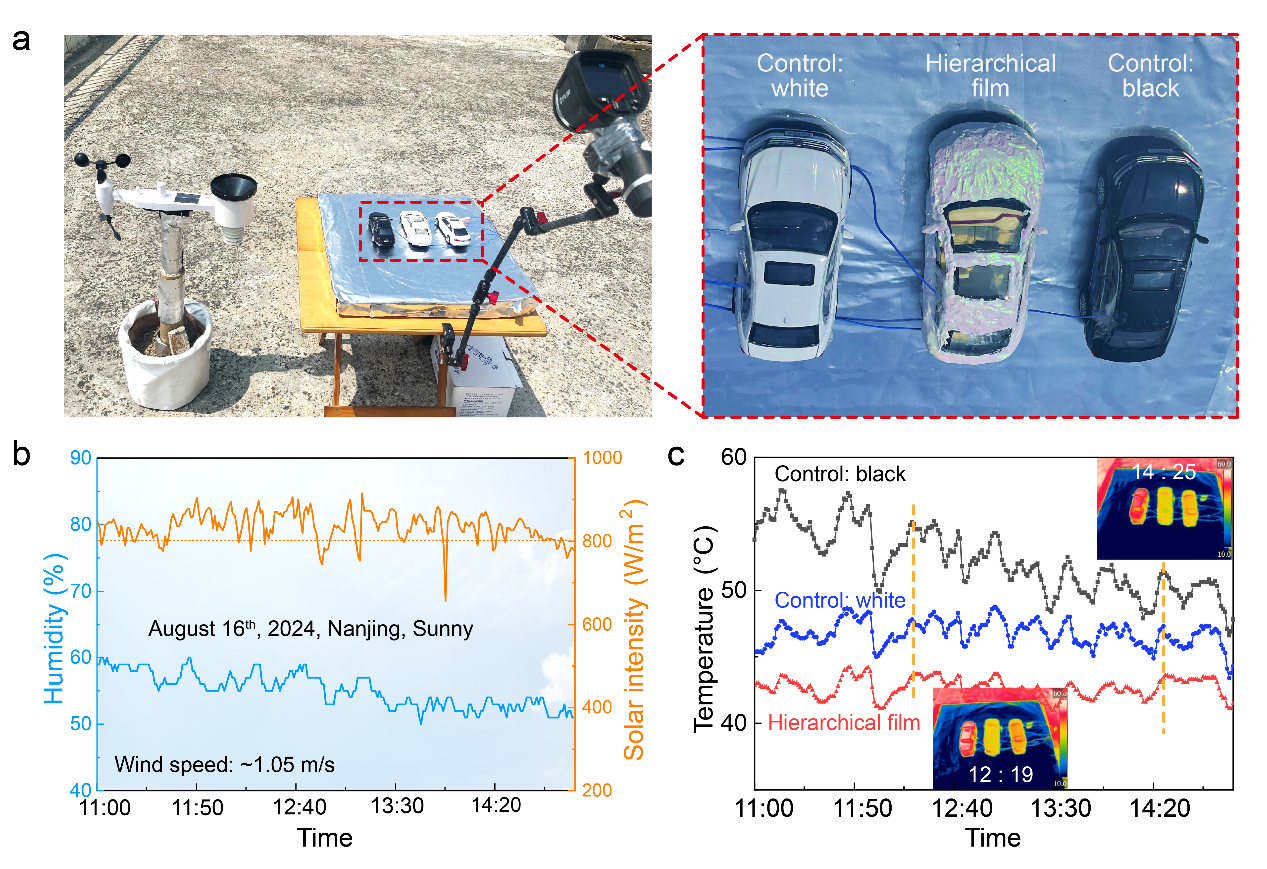


**Fig. S43 a** Digital photographs of the identical car models in outdoor experiments. **b** Weather parameters in Nanjing, China (32^o^6’N, 118^o^40’E) in August 16^th^ 2024. **c** Temperature test curves of the identical black car models exposed to the sun (black curve). Temperature data of the white car model (blue curve). And temperature tracking for the black car model coated by the hierarchical PDRC coating (red curve)


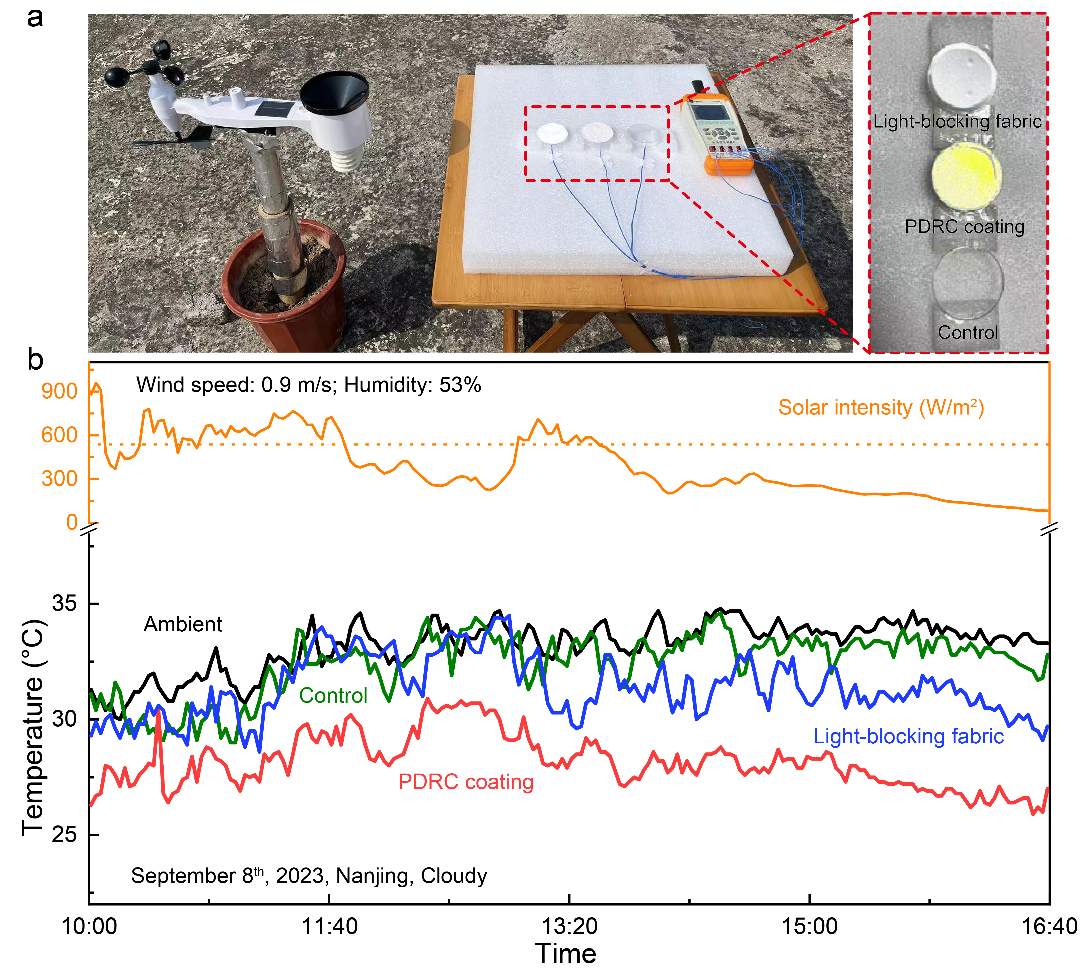


**Fig. S44 a** Photograph of the setup to evaluate the PDRC potential in shielded application. **b** Temperature tracking of control, light-blocking fabric, and PDRC coating samples in Nanjing, China (32^o^6’N, 118^o^40’E, September 8^th^, 2023). The ambient parameters, including ambient temperature, wind speed, humidity, and solar intensity were collected by a pint-sized automatic weather station

**Table S1** Synthesis of P(MMA-BA-MAA) colloidal latex using mono-component surfactant (SDS)

| Sample No. | SDS (g) | Monomer (g) | Particle size (nm) | PDI | SC (wt.%) |
| --- | --- | --- | --- | --- | --- |
| S-1-1 | 1.8 | 60 | 210 | 0.027 | 21.4 |
| S-1-2 | 2.2 | 60 | 187 | 0.022 | 23.8 |
| S-1-3 | 2.5 | 60 | 164 | 0.017 | 24.4 |
| S-1-4 | 1.8 | 90 | 260 | 0.062 | 28.5 |
| S-1-5 | 2.2 | 90 | 234 | 0.053 | 31.7 |
| S-1-6 | 2.5 | 90 | 217 | 0.044 | 30.5 |
| S-1-7 | 2.5 | 120 | 263 | 0.092 | 39.2 |
| S-1-8 | 2.5 | 150 | \ | \ | \ |
| S-1-9 | 3.2 | 150 | 335 | 0.173 | 44.9 |
| S-1-10 | 3.6 | 150 | 313 | 0.117 | 43.5 |

MMA: BA: MAA = 8:2:1 (mass ratio), H_2_O = 130 g, KPS = 0.5 g, NaHCO_3_ = 0.5 g, reaction temperature: 85 ℃, reaction time: 7 h.

**Table S2** Synthesis of P(MMA-BA-MAA) colloidal latex using dual-component surfactants (SDS and CO897)

| Sample No. | SDS (g) | CO897  (g) | Monomer (g) | Particle size (nm) | PDI | SC (wt.%) |
| --- | --- | --- | --- | --- | --- | --- |
| S-2-1 | 0.2 | 0.6 | 70 | 174 | 0.014 | 31 |
| S-2-2 | 0.2 | 0.6 | 90 | 196 | 0.054 | 36 |
| S-2-3 | 0.35 | 1.15 | 90 | 168 | 0.013 | 41 |
| S-2-4 | 0.35 | 1.15 | 130 | 245 | 0.016 | 45 |
| S-2-5 | 0.35 | 1.15 | 150 | 278 | 0.045 | 49 |
| S-2-6 | 0.45 | 1.35 | 170 | 291 | 0.033 | 52 |
| S-2-7 | 0.45 | 1.35 | 190 | 374 | 0.143 | 61 |
| S-2-8 | 0.5 | 1.5 | 190 | 343 | 0.082 | 63 |
| S-2-9 | 0.5 | 1.5 | 210 | \ | \ | \ |

MMA: BA: MAA = 8:2:1 (mass ratio), H_2_O = 130 g, KPS = 0.5 g, NaHCO_3_ = 0.5 g, reaction temperature: 85 ℃, reaction time: 7 h.

**Table S3** Effect of monomer amount on high SC P(MMA-BA-MAA) colloidal latex via the latex polymerization method

| Sample No. | SDS (g) | CO897  (g) | Monomer (g) | Particle size (nm) | PDI | SC (wt.%) |
| --- | --- | --- | --- | --- | --- | --- |
| S-3-1 | 0.45 | 1.35 | 70 | 174 | 0.007 | 31 |
| S-3-2 | 0.45 | 1.35 | 90 | 196 | 0.004 | 36 |
| S-3-3 | 0.45 | 1.35 | 110 | 220 | 0.013 | 41 |
| S-3-4 | 0.45 | 1.35 | 130 | 245 | 0.016 | 45 |
| S-3-5 | 0.45 | 1.35 | 150 | 268 | 0.025 | 49 |
| S-3-6 | 0.45 | 1.35 | 170 | 291 | 0.033 | 52 |
| S-3-7 | 0.45 | 1.35 | 180 | 324 | 0.047 | 55 |
| S-3-8 | 0.45 | 1.35 | 190 | 374 | 0.143 | 63 |
| S-3-9 | 0.45 | 1.35 | 200 | \ | \ | \ |

MMA: BA: MAA = 8:2:1 (mass ratio), H_2_O = 130 g, KPS = 0.5 g, NaHCO_3_ = 0.5 g, reaction temperature: 85 ℃, reaction time: 7 h.

**Table S4** Effect of surfactant amount on high SC P(MMA-BA-MAA) colloidal latex via the developed latex polymerization method

| Sample No. | Surfactant  (g) | Monomer (g) | Particle size (nm) | PDI | SC (wt.%) |
| --- | --- | --- | --- | --- | --- |
| S-4-1 | 0.8 | 170 | \ | \ | \ |
| S-4-2 | 1.0 | 170 | \ | \ | \ |
| S-4-3 | 1.2 | 170 | 376 | 0.172 | 45 |
| S-4-4 | 1.5 | 170 | 321 | 0.074 | 48 |
| S-4-5 | 1.8 | 170 | 291 | 0.033 | 52 |
| S-4-6 | 2.1 | 170 | 269 | 0.028 | 53 |
| S-4-7 | 2.4 | 170 | 245 | 0.017 | 51 |
| S-4-8 | 2.7 | 170 | 223 | 0.016 | 53 |
| S-4-9 | 3.0 | 170 | 202 | 0.014 | 52 |

MMA: BA: MAA = 8:2:1 (mass ratio), CO897: SDS = 3:1 (mass ratio), H_2_O = 130 g, KPS = 0.5g, NaHCO_3_ = 0.5g, reaction temperature: 85 ℃, reaction time: 7 h.

**Table S5** Summary of scale and optical property of representative large-scale CPC coatings

| Related work | FWHM  (nm) | Scale of area  (m^2^) |
| --- | --- | --- |
| Our work | 43 | 0.72 |
| 1 [S47] | 157 | 0.017 |
| 2 [S48] | 33 | 0.015 |
| 3 [S49] | 67 | 0.018 |
| 4 [S50] | 48 | 0.24 |
| 5 [S51] | 170 | 0.6 |

**Table S6** Outdoor experiments under typical summer weathers in Nanjing, China (32^o^6’N, 118^o^40’E)

| Date | Solar intensity  (W/m^2^) | Wind speed  (m/s) | Humidity  (%) | Average sub-ambient *ΔT* (℃) | Maximum sub-ambient *ΔT*  (℃) |
| --- | --- | --- | --- | --- | --- |
| Aug. 21^th^ | 632 | 1.21 | 63 | 5.2 | 7.9 |
| Aug. 23^th^ | 622 | 1.00 | 59.5 | 4.6 | 7.2 |
| Aug. 24^th^ | 580 | 0.75 | 50.6 | 5.0 | 8.1 |
| Aug. 25^th^ | 637 | 0.84 | 46.7 | 5.6 | 8.0 |
| Aug. 30^th^ | 320 | 0.61 | 71.6 | 6.6 | 9.1 |
| Sep. 1^th^ | 714 | 1.20 | 48.7 | 4.7 | 7.8 |

**Table S7** Economic analysis for representative large-scale PDRC designs

| Related work | Raw material cost (CNY/m^2^) | Manufacturing costs  (CNY/m^2^) | Total cost (CNY/m^2^) |
| --- | --- | --- | --- |
| 1 (Our work) | ~44 | ~20 | ~64 |
| 2 [S32] | ~55 | ~20 | ~75 |
| 3 [S52] | ~35 | ~20 | ~55 |
| 4 [S53] | ~670 | ~60 | ~730 |
| 5 [S54] | ~61 | ~20 | ~81 |
| 6 [S27] | ~450 | ~20 | ~470 |
| 7 [S55] | ~115 | ~50 | ~165 |
| 8 [S25] | ~335 | ~20 | ~355 |

**Table S8** Summary of representative large-scale PDRC designs

| Related work | $P_{cool}\left( T_{s} \right)$ (W/m^2^) | Main  Materials | Scale of area (m^2^) | Substrate | Method | Sub-ambient Δ*T* (℃) | Total cost (CNY/m^2^) |
| --- | --- | --- | --- | --- | --- | --- | --- |
| 1 (Our work) | 95.5 (Theoretical) | CPC and SiO_2_ | Meter scale | Versatility | Paint-like applicability | ~5.3  (584 W/m^2^) | ~64 |
| 2 [S32] | 45.7  (Measured) | Styrene-acrylate emulsion and hollow glass microspheres | ~20 | Versatility | Paint-like applicability | ~3.5  (~996 W/m^2^) | ~75 |
| 3 [S52] | 64.5  (Theoretical) | TiO_2_ particles and glass microspheres | Meter scale | Versatility | Paint-like applicability | ~6  (~744 W/m^2^) | ~55 |
| 4 [S53] | 74.4  (Theoretical) | Structural polymer | ~0.06 | Self-supporting | Etching-Inverting | ~5.5  (~930 W/m^2^) | ~730 |
| 5 [S54] | 16 (Measured) | Wood | ~0.1 | Self-supporting | Delignification and densification | ~4  (middy) | ~81 |
| 6 [S27] | 96 (Measured) | Solvent and polymer | ~0.1 | Versatility | Phase  inversion | ~6  (~820 W/m^2^) | ~470 |
| 7 [S55] | ~50  (Theoretical) | TiO_2_ particles and polylactic acid | ~4.5 | Self-supporting | Melt-spinning and weaving | ~2  (~580 W/m^2^) | ~165 |
| 8 [S25] | 93 (Measured) | Glass-polymer | 5 m/min | Silver film. | roll-to-roll manner | ~10 (night) | ~355 |

**Table S9** Summary of representative colored PDRC designs

| Related work | $P_{cool}\left( T_{s} \right)$ (W/m^2^) | Mechanism | Coloring  Materials | Scale of area (m^2^) | Method | Δ*T* (℃) |
| --- | --- | --- | --- | --- | --- | --- |
| 1 (Our work) | 95.5 (Theoretical) | Selective narrowband reflection | CPCs | Meter scale | Paint-like applicability | ~5.3  (584 W/m^2^) |
| 2 [56] | 69.2  (Theoretical) | Selectively absorbance | Photoluminescent CDs | Centimeter scale | Coaxial wet-spinning | ~5.1  (~923 W/m^2^) |
| 3 [57] | / | Selective reflection | ZnS@SiO_2_ colloidal crystals | Decimeter scale | Spray coating | 5-7  (800 W/m^2^, simulated skin) |
| 4 [38] | 74.4  (Measured) | Interferometric Retroreflection | PS microsphere monolayer | Centimeter scale | unidirectional rubbing | 4  (>1000 W/m^2^) |
| 5 [37] | / | Selective reflection | Cellulose nanocrystals | Meter scale | Roll-to-Roll Deposition | ~1.4  (~900 W/m^2^) |
| 6 [58] | / | Selectively absorbance | Thermochromic microcapsules | Meter scale | Asymmetric deposition | ~6  (~820 W/m^2^) |
| 7 [59] | / | Thin-film resonation | TiO_2_ and Si_3_N_4_ bilayer | Centimeter scale | Layer-by-layer steaking | ~3.9  (~800 W/m^2^) |

**Video S1** Tracking for colloidal skin formation

**Video S2** Construction of CPC coating with high efficiency on building cement

**Video S3** Water resistance test for the colored PDRC coating under flowing stream

**Video S4** Wear fastness of the colored PDRC coating

**Supplementary References**

1. P.A. Lovell, F.J. Schork, Fundamentals of emulsion polymerization. Biomacromolecules **21**(11), 4396–4441 (2020). <https://doi.org/10.1021/acs.biomac.0c00769>
2. H. Cui, G. Cao, S. Zhu, J. Mu, X. Chou, Study on the preparation and formation factors of frother emulsion. Colloids Surf. A Physicochem. Eng. Aspects **636**, 128155 (2022). <https://doi.org/10.1016/j.colsurfa.2021.128155>
3. T. Song, F. Gao, S. Guo, Y. Zhang, S. Li et al., A review of the role and mechanism of surfactants in the morphology control of metal nanoparticles. Nanoscale **13**(7), 3895–3910 (2021). <https://doi.org/10.1039/D0NR07339C>
4. A. Guyot, F. Chu, M. Schneider, C. Graillat, T.F. McKenna, High solid content latexes. Prog. Polym. Sci. **27**(8), 1573–1615 (2002). <https://doi.org/10.1016/S0079-6700(02)00014-X>
5. M. Han, H. Wu, E. Luijten, Electric double layer of anisotropic dielectric colloids under electric fields. Eur. Phys. J. Spec. Top. **225**(4), 685–698 (2016). <https://doi.org/10.1140/epjst/e2015-50316-9>
6. D. Yang, Y. Qin, S. Ye, J. Ge, Polymerization-induced colloidal assembly and photonic crystal multilayer for coding and decoding. Adv. Funct. Mater. **24**(6), 817–825 (2014). <https://doi.org/10.1002/adfm.201301590>
7. J. Zhang, Z. Zhu, Z. Yu, L. Ling, C.-F. Wang et al., Large-scale colloidal films with robust structural colors. Mater. Horiz. **6**(1), 90–96 (2019). <https://doi.org/10.1039/c8mh00248g>
8. Y. Suh, Q. Pham, B. Shao, Y. Won, The control of colloidal grain boundaries through evaporative vertical self-assembly. Small **15**(12), e1804523 (2019). <https://doi.org/10.1002/smll.201804523>
9. J. Zhou, J. Yang, Z. Gu, G. Zhang, Y. Wei et al., Controllable fabrication of noniridescent microshaped photonic crystal assemblies by dynamic three-phase contact line behaviors on superhydrophobic substrates. ACS Appl. Mater. Interfaces **7**(40), 22644–22651 (2015). <https://doi.org/10.1021/acsami.5b07443>
10. Z. Cai, Z. Li, S. Ravaine, M. He, Y. Song et al., From colloidal particles to photonic crystals: advances in self-assembly and their emerging applications. Chem. Soc. Rev. **50**(10), 5898–5951 (2021). <https://doi.org/10.1039/d0cs00706d>
11. H. Hwang, Y.C. Cho, S. Lee, T.M. Choi, S.-H. Kim et al., Real-time monitoring of colloidal crystallization in electrostatically-levitated drops. Small **16**(11), e1907478 (2020). <https://doi.org/10.1002/smll.201907478>
12. Z. Yu, C.-F. Wang, L. Ling, L. Chen, S. Chen, Triphase microfluidic-directed self-assembly: anisotropic colloidal photonic crystal supraparticles and multicolor patterns made easy. Angew. Chem. Int. Ed. **51**(10), 2375–2378 (2012). <https://doi.org/10.1002/anie.201107126>
13. J. Wang, U. Sultan, E.S.A. Goerlitzer, C.F. Mbah, M. Engel et al., Structural color of colloidal clusters as a tool to investigate structure and dynamics. Adv. Funct. Mater. **30**(26), 1907730 (2020). <https://doi.org/10.1002/adfm.201907730>
14. S.-H. Kim, J.-G. Park, T.M. Choi, V.N. Manoharan, D.A. Weitz, Osmotic-pressure-controlled concentration of colloidal particles in thin-shelled capsules. Nat. Commun. **5**, 3068 (2014). <https://doi.org/10.1038/ncomms4068>
15. W. Hong, Z. Yuan, X. Chen, Structural color materials for optical anticounterfeiting. Small **16**(16), 1907626 (2020). <https://doi.org/10.1002/smll.201907626>
16. K. Keller, A.V. Yakovlev, E.V. Grachova, A.V. Vinogradov, Inkjet printing of multicolor daylight visible opal holography. Adv. Funct. Mater. **28**(21), 1706903 (2018). <https://doi.org/10.1002/adfm.201706903>
17. H.H. Kim, E. Im, S. Lee, Colloidal photonic assemblies for colorful radiative cooling. Langmuir **36**(23), 6589–6596 (2020). <https://doi.org/10.1021/acs.langmuir.0c00051>
18. L. Zhou, H. Song, J. Liang, M. Singer, M. Zhou et al., A polydimethylsiloxane-coated metal structure for all-day radiative cooling. Nat. Sustain. **2**(8), 718–724 (2019). <https://doi.org/10.1038/s41893-019-0348-5>
19. Z. Zhu, J. Zhang, C.-F. Wang, S. Chen, Construction of hydrogen-bond-assisted crack-free photonic crystal films and their performance on fluorescence enhancement effect. Macromol. Mater. Eng. **302**(6), 1700013 (2017). <https://doi.org/10.1002/mame.201700013>
20. B. Yi, H. Shen, Facile fabrication of crack-free photonic crystals with enhanced color contrast and low angle dependence. J. Mater. Chem. C **5**(32), 8194–8200 (2017). <https://doi.org/10.1039/C7TC01549F>
21. Y. Cui, F. Wang, J. Zhu, W. Wu, Y. Qin et al., Preparation of large-scale and angle-independent structural colors by additive of black polypyrrole. J. Colloid Interface Sci. **531**, 609–617 (2018). <https://doi.org/10.1016/j.jcis.2018.07.094>
22. I. Jurewicz, A.A.K. King, R. Shanker, M.J. Large, R.J. Smith et al., Mechanochromic and thermochromic sensors based on graphene infused polymer opals. Adv. Funct. Mater. **30**(31), 2002473 (2020). <https://doi.org/10.1002/adfm.202002473>
23. D.S. Rickerby, A review of the methods for the measurement of coating-substrate adhesion. Surf. Coat. Technol. **36**(1–2), 541–557 (1988). <https://doi.org/10.1016/0257-8972(88)90181-8>
24. A.-Q. Xie, H. Qiu, W. Jiang, Y. Wang, S. Niu et al., Recent advances in spectrally selective daytime radiative cooling materials. Nanomicro Lett. **17**(1), 264 (2025). <https://doi.org/10.1007/s40820-025-01771-8>
25. Y. Zhai, Y. Ma, S.N. David, D. Zhao, R. Lou et al., Scalable-manufactured randomized glass-polymer hybrid metamaterial for daytime radiative cooling. Science **355**(6329), 1062–1066 (2017). <https://doi.org/10.1126/science.aai7899>
26. Y. Zhao, R. Li, B. Wang, Y. Huang, P. Lyu et al., Scalable structural coloration of carbon nanotube fibers *via* a facile silica photonic crystal self-assembly strategy. ACS Nano **17**(3), 2893–2900 (2023). <https://doi.org/10.1021/acsnano.2c11296>
27. J. Mandal, Y. Fu, A.C. Overvig, M. Jia, K. Sun et al., Hierarchically porous polymer coatings for highly efficient passive daytime radiative cooling. Science **362**(6412), 315–319 (2018). <https://doi.org/10.1126/science.aat9513>
28. X. Yin, R. Yang, G. Tan, S. Fan, Terrestrial radiative cooling: Using the cold universe as a renewable and sustainable energy source. Science **370**(6518), 786–791 (2020). <https://doi.org/10.1126/science.abb0971>
29. Y. Peng, L. Fan, W. Jin, Y. Ye, Z. Huang et al., Coloured low-emissivity films for building envelopes for year-round energy savings. Nat. Sustain. **5**(4), 339–347 (2022). <https://doi.org/10.1038/s41893-021-00836-x>
30. Z. Cheng, Y. Shuai, D. Gong, F. Wang, H. Liang et al., Optical properties and cooling performance analyses of single-layer radiative cooling coating with mixture of TiO_2_ particles and SiO_2_ particles. Sci. China Technol. Sci. **64**(5), 1017–1029 (2021). <https://doi.org/10.1007/s11431-020-1586-9>
31. L. Du, Z. Zhou, J. Li, B. Hu, C. Wang et al., Highly efficient subambient all-day passive radiative cooling textiles with optically responsive MgO embedded in porous cellulose acetate polymer. Chem. Eng. J. **469**, 143765 (2023). <https://doi.org/10.1016/j.cej.2023.143765>
32. Y. Li, Y. Zhang, Z. Yang, X. Xue, Z. He et al., Waterborne coatings with sub-ambient cooling under direct sunlight–part I: Optical properties and cooling effect measurements. Sol. Energy Mater. Sol. Cells **217**, 110672 (2020). <https://doi.org/10.1016/j.solmat.2020.110672>
33. M.M. Hossain, M. Gu, Radiative cooling: principles, progress, and potentials. Adv. Sci. **3**(7), 1500360 (2016). <https://doi.org/10.1002/advs.201500360>
34. X. Lu, P. Xu, H. Wang, T. Yang, J. Hou, Cooling potential and applications prospects of passive radiative cooling in buildings: The current state-of-the-art. Renew. Sustain. Energy Rev. **65**, 1079–1097 (2016). <https://doi.org/10.1016/j.rser.2016.07.058>
35. B. Zhu, W. Li, Q. Zhang, D. Li, X. Liu et al., Subambient daytime radiative cooling textile based on nanoprocessed silk. Nat. Nanotechnol. **16**(12), 1342–1348 (2021). <https://doi.org/10.1038/s41565-021-00987-0>
36. P. Xu, B. Xiang, W. Zhong, Y. Wu, Y. Zhang et al., Biodegradable, scalable and flexible fiber membrane for green passive radiative cooling. Sol. Energy Mater. Sol. Cells **253**, 112209 (2023). <https://doi.org/10.1016/j.solmat.2023.112209>
37. W. Zhu, B. Droguet, Q. Shen, Y. Zhang, T.G. Parton et al., Structurally colored radiative cooling cellulosic films. Adv. Sci. **9**(26), 2202061 (2022). <https://doi.org/10.1002/advs.202202061>
38. S. Yu, Q. Zhang, Y. Wang, Y. Lv, R. Ma, Photonic-structure colored radiative coolers for daytime subambient cooling. Nano Lett. **22**(12), 4925–4932 (2022). <https://doi.org/10.1021/acs.nanolett.2c01570>
39. L. Zhou, X. Yin, Q. Gan, Best practices for radiative cooling. Nat. Sustain. **6**(9), 1030–1032 (2023). <https://doi.org/10.1038/s41893-023-01170-0>
40. X. Liu, P. Li, Y. Liu, C. Zhang, M. He et al., Hybrid passive cooling for power equipment enabled by metal-organic framework. Adv. Mater. **36**(45), e2409473 (2024). <https://doi.org/10.1002/adma.202409473>
41. W. Lin, X. Yao, N.M. Kumar, W.K. Lo, S.S. Chopra et al., Camel-fur-inspired graphite-based hygroscopic membrane for passive air cooling with ultrahigh cooling power. Adv. Energy Mater. **14**(16), 2303470 (2024). <https://doi.org/10.1002/aenm.202303470>
42. X. Zhao, T. Li, H. Xie, H. Liu, L. Wang et al., A solution-processed radiative cooling glass. Science **382**(6671), 684–691 (2023). <https://doi.org/10.1126/science.adi2224>
43. D. Yan, T. Hong, C. Li, Q. Zhang, J. An et al., A thorough assessment of China’s standard for energy consumption of buildings. Energy Build. **143**, 114–128 (2017). <https://doi.org/10.1016/j.enbuild.2017.03.019>
44. X. Yang, L.L.H. Peng, Y. Chen, L. Yao, Q. Wang, Air humidity characteristics of local climate zones: a three-year observational study in Nanjing. Build. Environ. **171**, 106661 (2020). <https://doi.org/10.1016/j.buildenv.2020.106661>
45. M. Wang, Q. Li, F. Wang, Z. Yuan, L. Wang et al., Residential indoor thermal environment investigation and analysis on energy saving of air conditioning in hot summer and warm winter zone in China. Urban Clim. **47**, 101369 (2023). <https://doi.org/10.1016/j.uclim.2022.101369>
46. Ł. Kampa, Ł. Sadowski, A. Królicka, The use of synthetic and natural fibers in epoxy coatings: a comparative mechanical and economic analysis. Int. J. Adhes. Adhes. **117**, 103017 (2022). <https://doi.org/10.1016/j.ijadhadh.2021.103017>
47. F. Meng, M.M. Umair, K. Iqbal, X. Jin, S. Zhang et al., Rapid fabrication of noniridescent structural color coatings with high color visibility, good structural stability, and self-healing properties. ACS Appl. Mater. Interfaces **11**(13), 13022–13028 (2019). <https://doi.org/10.1021/acsami.9b01522>
48. C. Wang, X. Lin, C.G. Schäfer, S. Hirsemann, J. Ge, Spray synthesis of photonic crystal based automotive coatings with bright and angular-dependent structural colors. Adv. Funct. Mater. **31**(9), 2008601 (2021). <https://doi.org/10.1002/adfm.202008601>
49. L. Bai, V.C. Mai, Y. Lim, S. Hou, H. Möhwald et al., Large-scale noniridescent structural color printing enabled by infiltration-driven nonequilibrium colloidal assembly. Adv. Mater. **30**(9), 1705667 (2018). <https://doi.org/10.1002/adma.201705667>
50. Y. He, L. Liu, Q. Fu, J. Ge, Precise assembly of highly crystalline colloidal photonic crystals inside the polyester yarns: a spray coating synthesis for breathable and durable fabrics with saturated structural colors. Adv. Funct. Mater. **32**(24), 2200330 (2022). <https://doi.org/10.1002/adfm.202200330>
51. W. Fan, J. Zeng, Q. Gan, D. Ji, H. Song et al., Iridescence-controlled and flexibly tunable retroreflective structural color film for smart displays. Sci. Adv. **5**(8), eaaw8755 (2019). <https://doi.org/10.1126/sciadv.aaw8755>
52. X. Xue, M. Qiu, Y. Li, Q.M. Zhang, S. Li et al., Creating an eco-friendly building coating with smart subambient radiative cooling. Adv. Mater. **32**(42), e1906751 (2020). <https://doi.org/10.1002/adma.201906751>
53. T. Wang, Y. Wu, L. Shi, X. Hu, M. Chen et al., A structural polymer for highly efficient all-day passive radiative cooling. Nat. Commun. **12**(1), 365 (2021). <https://doi.org/10.1038/s41467-020-20646-7>
54. T. Li, Y. Zhai, S. He, W. Gan, Z. Wei et al., A radiative cooling structural material. Science **364**(6442), 760–763 (2019). <https://doi.org/10.1126/science.aau9101>
55. S. Zeng, S. Pian, M. Su, Z. Wang, M. Wu et al., Hierarchical-morphology metafabric for scalable passive daytime radiative cooling. Science **373**(6555), 692–696 (2021). <https://doi.org/10.1126/science.abi5484>
56. J. Liu, B. Ji, Y. Zhong, L. Zhang, B. Wang et al., Photoluminescent core–shell structural porous fibers-based metafabric for colored daytime passive radiative cooling. ACS Nano **19**(10), 10263–10276 (2025). <https://doi.org/10.1021/acsnano.4c17745>
57. L. Du, R. Li, W. Chen, Colored textiles based on noniridescent structural color of ZnS@SiO_2_ colloidal crystals for daytime passive radiative cooling. Chem. Eng. J. **475**, 146431 (2023). <https://doi.org/10.1016/j.cej.2023.146431>
58. B.-Y. Liu, J. Wu, C.-H. Xue, Y. Zeng, J. Liang et al., Bioinspired superhydrophobic all-In-one coating for adaptive thermoregulation. Adv. Mater. **36**(31), e2400745 (2024). <https://doi.org/10.1002/adma.202400745>
59. G.J. Lee, Y.J. Kim, H.M. Kim, Y.J. Yoo, Y.M. Song, Colored, daytime radiative coolers with thin-film resonators for aesthetic purposes. Adv. Opt. Mater. **6**(22), 1800707 (2018). <https://doi.org/10.1002/adom.201800707>
